# Enhanced Chiral Recognition by Cyclodextrin Dimers

**DOI:** 10.3390/ijms12074637

**Published:** 2011-07-18

**Authors:** Jens Voskuhl, Kira Schaepe, Bart Jan Ravoo

**Affiliations:** Organic Chemistry Institute, Westfälische Wilhelms-Universität Münster, Correnstrasse 40, Münster 48149, Germany; E-Mails: jens.voskuhl@uni-muenster.de (J.V.); k_scha19@uni-muenster.de (K.S.)

**Keywords:** cyclodextrins, chiral recognition, host-guest complexes, isothermal titration calorimetry, multivalency

## Abstract

In this article we investigate the effect of multivalency in chiral recognition. To this end, we measured the host-guest interaction of a β-cyclodextrin dimer with divalent chiral guests. We report the synthesis of carbohydrate-based water soluble chiral guests functionalized with two borneol, menthol, or isopinocampheol units in either (+) or (−) configuration. We determined the interaction of these divalent guests with a β-cyclodextrin dimer using isothermal titration calorimetry. It was found that—in spite of a highly unfavorable conformation—the cyclodextrin dimer binds to guest dimers with an increased enantioselectivity, which clearly reflects the effect of multivalency.

## 1. Introduction

Cyclodextrins (CDs) are artificial chiral receptors that bind numerous hydrophobic chiral molecules and are well known as stationary phases in liquid chromatography and capillary electrophoresis [1]. Most research efforts to investigate the interaction of enantiomeric guests with the chiral cavity of CDs have focused on *monovalent* inclusion complexes with only a single interaction between a hydrophobic guest and a CD host. However, the binding constants of enantiomeric guest molecules usually show only marginal differences and in consequence a very modest degree of chiral recognition is observed [2–7]. The introduction of substituents such as nucleobases to the primary face of β-CD leads to an increase in chiral recognition of natural compounds such as borneol and camphor [8]. This effect is based on an additional stabilization induced by secondary interactions such as hydrogen bonding and electrostatics.

It is our hypothesis that *multivalent* complexes involving multiple host-guest interactions will not only result in an increased overall binding affinity (expressed as Δ*G* or *K*_a_) but also induce a significant increase in enantioselectivity (expressed as Δ Δ*G*). Multivalency plays a key role in cell-cell and cell-matrix recognition processes in biological systems [9]. In recent years, a variety of model systems to study multivalency were investigated. Such model systems include dendrimers [10], polymers [11], nanoparticles [12], vesicles [13,14] and self-assembled monolayers [15]. In the majority of cases, multivalent interactions are additive—not cooperative—and lead to higher overall binding affinities (*K*_a_), lower dissociation rates (*k*_d_), and higher selectivities.

As a proof-of-principle, we investigated the divalent interaction of dimers of chiral guests with β-CD dimers. CD dimers with various linkers are readily available and their interaction with monovalent as well as divalent guests has been investigated in detail, although mainly with achiral hydrophobic guests [16–27]. The interaction of CD dimers with divalent guests can either lead to divalent 2:2 macrocyclic complexes or extended supramolecular polymers, depending on the geometry and the concentration of host and guest [28–30]. In some cases it is possible to switch between monovalent, divalent and multivalent complexes by external stimulation [31,32].

In this contribution, we report the interaction of chiral divalent guest molecules based on borneol, isopinocampheol and menthol. We investigated whether a change from a monovalent (1:1) to a divalent (2:2) complexation leads to a significant increase in binding affinity and/or selectivity. To this end, water soluble dimers of borneol, isopinocampheol and menthol in (+) and (−) configuration as well as a β-CD dimer were synthesized. The thermodynamic parameters of the interaction between the dimers and β-CD and the β-CD dimer, respectively, were measured by isothermal titration calorimetry (ITC).

## 2. Results and Discussion

**Scheme 1:**
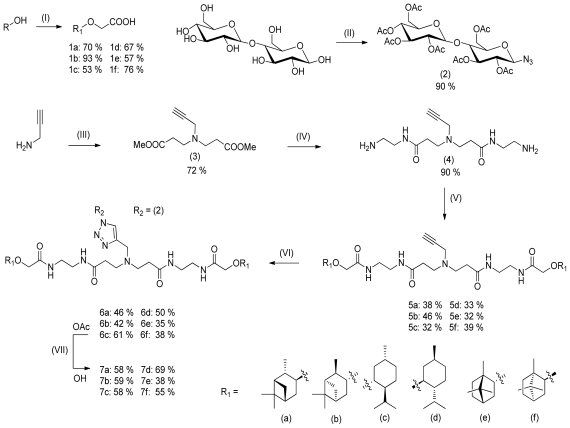
Synthesis of the chiral water soluble guest dimers **7a**–**f**. (I) BrCH_2_COOH, NaH, dioxane, 24 h; (II) a. I_2_, Ac_2_O, 10 min, b. TMSN_3_, SnCl_4_, CH_2_Cl_2_, 12 h; (III) CH_2_CHCOOCH_3_, 48 h; (IV) NH_2_C_2_H_4_NH_2_, 72 h; (V) EDCI, Oxyma pur ^©^, NMM, DMF, 18 h; (VI) CuSO_4_, sodium ascorbate, DMF, H_2_O, 18 h; (VII) NaOMe, MeOH, Dowex HWCR 20, 12 h.

To investigate the chiral recognition between guest and host dimers, a set of water soluble molecules based on menthol, borneol and isopinocampheol either in (+) and (−) configuration were synthesized. The water solubility was improved by conjugation of a maltose moiety to the spacer that links the two hydrophobic guests. The synthesis is outlined in Scheme 1. The secondary chiral alcohols (either in (+) or (−) configuration) were converted into the carboxylic acid derivatives **1a**–**1f** via *Williamson* ether synthesis by reaction with bromoacetic acid using sodium hydride as a base. Maltose was peracetylated and converted into azide **2**. Synthesis of the dimers started out from propargylamine which was converted into the corresponding dimethylester **3** via *Michael* addition. By reacting **3** with an excess of ethylenediamine, alkyne functionalized diamine **4** was obtained in quantitative yield. The hydrophobic guest units **1a**–**f** were coupled to **4** under peptide coupling conditions with EDCI and NMM in DMF to provide **5a**–**f**. Azide **2** was conjugated to **5a**–**f** by a Cu(I) catalyzed click reaction to give **6a**–**f**. Dimers **7a**–**f** were obtained by deprotection of the acetyl functions using *Zemplèn* conditions. Experimental details and analytical data are provided as supporting information. A selection of ^1^H and ^13^C NMR spectra is also provided. The NMR and MS data are consistent with the molecular structures.

**Scheme 2:**
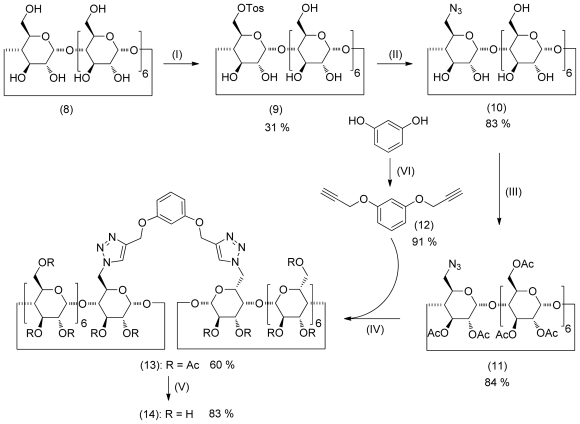
Synthesis of the cyclodextrin dimer **14**. (I) TosCl, NaOH, H_2_O, 18 h; (II) NaN_3_, DMF, 60 °C, 18 h; (III) Ac_2_O, CH_2_Cl_2_, 12 h; (IV) CuSO_4_, sodium ascorbate, DMF, H_2_O, 18 h; (V) NaOMe, MeOH, Dowex HWCR 20, 12 h; (VI) BrCH_2_CCH, K_2_CO_3_, acetone, 18 h.

The dimeric CD host was synthesized according to a known literature procedure [33]. However, in contrast to the literature procedure, the peracetylated monoazide of β-CD was used. The synthesis is outlined in Scheme 2. The β-CD was converted to the monoazide **10** by reaction of monotosylate **9** with sodium azide. After peracetylation of **10,** the protected monazide CD **11** was obtained in good yield. After Cu(I) catalyzed click reaction of CD **11** with dialkyne linker **12** followed by deprotection of the acetyl functions under *Zemplèn* conditions, the CD dimer **14** was obtained in good yields. Experimental details and analytical data are provided as supporting information. The NMR and MS data are consistent with the molecular structures.

**Figure 1:**
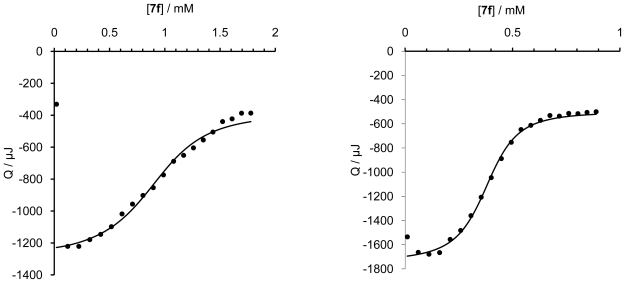
Isothermal titration calorimetry (ITC) measurements of (*left*) **7f** with β-cyclodextrin ([**7f**] = 5 mM, [β-CD] = 1 mM) and (*right*) **7f** with dimer **14** ([**7f**] = 5 mM, [**14**] = 0.5 mM). Titrations were performed in water at 25 °C with 20 injections with a volume of 10 μL.

The chiral recognition of the guest molecules **7a**–**f** with β-CD as well as with CD dimer **14** as host molecules was investigated using ITC. In all ITC experiments, guests **7a**–**f** were titrated into host **14** (or β-CD) in water at 25 °C. Deionized water (rather than buffer solutions) was used for all ITC experiments, since both hosts and guests are non-ionic and buffers might complicate the ITC analysis due to salt effects. In order to compare the ITC data for the monovalent interaction with β-CD and the divalent interaction with dimer **14**, the concentration of guests and hosts were set as follows: [**1a**–**f**] = 5 mM, [β-CD] = 1 mM, [**14**] = 0.5 mM. Two representative titrations are shown in Figure 1. Additional titrations are provided in the supporting information. The ITC data are summarized in Table 1.

It was observed that the monovalent interaction of **7a**–**f** with β-CD gave weak to moderate binding constants with only weak preferences for the (+) or (−) divalent guest molecules (*K*_a_ = 0.83–2.00 × 10^4^ M^−1^ and Δ Δ*G* = 0.5–1.4 kJ mol^−1^). Host-guest inclusion is both enthalpically and entropically favourable. These findings are in good agreement with the data for the 1:1 interaction of the free secondary alcohols with β-CD [5].

**Figure 2:**
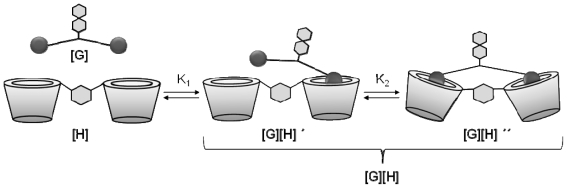
Stepwise binding modes for the interaction of a dimeric guest with a dimeric host molecule.

The interaction of **7a**–**f** with CD dimer **14** gave significantly higher binding constants (*K*_a_ = 3.14–11.21 × 10^4^ M^−1^). These binding constants are diagnostic of a divalent interaction. However, as will be shown in the following discussion, the divalent interaction is much less efficient than anticipated on the basis of the design of guest dimers **7a**–**f** and host dimer **14**. The divalent binding process can be dissected into an intermolecular binding event followed by an intramolecular binding event, which can be quantified in terms of an intrinsic binding constant *K*_i_ and an effective concentration *C*_eff_ [25]. The stepwise model for divalent interaction is illustrated in Figure 2.

The first binding step can be interpreted as an intermolecular binding event with binding constant *K*_1_, which is directly correlated to the intrinsic binding constant *K*_i_. The factor 4 in Equation 1 is based on the four degenerate binding modes for the first complexation step.

(1) $$K_{1}=4K_{\text{i}}$$

The second binding step with binding constant *K*_2_ is an intramolecular process which can be expressed by the intrinsic binding constant *K*_i_ and the effective concentration *C*_eff_. *C*_eff_ describes the concentration of host available to the second guest unit after the first guest unit is bound to the dimer. This expression is used to differ between inter- and intramolecular complexation. The factor ½ in Equation 2 is due to the fact that the concentration of free host halves after the first complexation step.

(2) $$K_{2}=1/2\;C_{\text{eff}}\;K_{\text{i}}$$

Since the overall binding constant *K*_a_ is the product of *K*_1_ and *K*_2_, it is possible to calculate C_eff_ from *K*_a_ and *K*_i_.

(3) $$K_{\text{a}}=K_{1}\times K_{2}=2C_{\text{eff}}\;{\left(K_{\text{i}}\right)}^{2}$$

(4) $$C_{\text{eff}}=\frac{K\text{a}}{{\left(K_{\text{i}}\right)}^{2}}\times \frac{1}{2}$$

Alternatively, a theoretical approximation of *C*_eff_ can be obtained from Equation 5.

(5) $$C_{\text{eff}}=\frac{1}{N_{\text{AV}}}\left(\frac{3}{\left(4\pi r_{0}{}^{3}\right)}\right)$$

Equation 5 describes the possibility of two linked chain ends to react in a defined volume. In Equation 5,*N*_Av_ is the Avogadro constant and r_0_ is the distance between the two guest moieties of **7a**–**f**. For these guest dimers *r*_0_ was estimated to be around 2 nm. According to Equation 5, a *C*_eff_ ≈ 50 mM would thus be expected for a divalent interaction of guests **7a**–**f** with host **14**.

**Figure 3:**
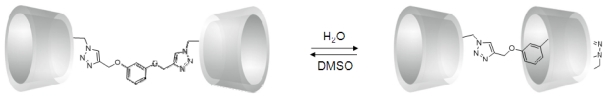
Schematic representation of the rotaxane-like self-complexation via a single glucose rotation of CD dimer **14** [33].

However, the ITC data indicate an interaction that is far from optimal. *K*_a_ can be directly obtained from the ITC titration of **7a**–**f** with **14**, while *K*_i_ can be obtained from the titrations with β-CD. Equation 4 then provides the values for *C*_eff_. It can be seen from Table 1 that the observed *C*_eff_ is in the range 0.126–0.268 mM which is two orders of magnitude lower than the value calculated from Equation 5! In other words, the interaction of **7a**–**f** is only partially divalent, and a substantial fraction of the divalent guest is bound in a monovalent mode only. An explanation for this unexpected finding was reported by *Monflier* and coworkers during the course of our investigation [34]. This group showed that a single glucose unit of the β-CD in dimer **14** forms a rotational isomer which leads to a pseudorotaxane-like structure (Figure 3). This preferred conformation leads to an effective blocking of one cavity, which strongly diminishes the binding behavior towards the chiral dimers **7a**–**f**. Indeed, as shown in Table 1, the binding stoichiometry of **14** and **7a**–**f** is not 1:1 as expected but significantly lower, indicating that not all CD cavities are available for complexation. This behavior is in good agreement with the ^1^H-NMR spectra of **14** which shows doubled signals for the aromatic protons [33]. The triazole protons give three particularly well-separated signals: two for the unsymmetric pseudorotaxane like structure and one for the free symmetric CD dimer. In the study by *Monflier* and coworkers, the amount of the symmetric species in aqueous media was determined to be as little as 20%. It was also shown that a monovalent interaction with adamantyl carboxylate does not lead to a reversible glucose rotation. Interestingly, the stoichiometry obtained by ITC is in the range between 0.7 and 0.8 indicating around 50% free dimer in solution. Also the observed binding constants are clearly indicative of a partial divalent interaction. On the basis of these observations, one can conclude that the rotation of the glucose unit is at least partially reversible and is strongly dependent on the guest and its binding affinity towards β-CDs. It can be assumed that upon divalent binding of **7a–f** the asymmetric pseudorotaxane structure is partially isomerized to the symmetric inclusion complex. Also, the entropy of complexation points to a significant degree of reorganization upon divalent binding. It is evident from Table 1 that, in comparison to the monovalent interaction with β-CD, the divalent interaction with CD dimer **14** is entropically disfavoured.

**Figure 4:**
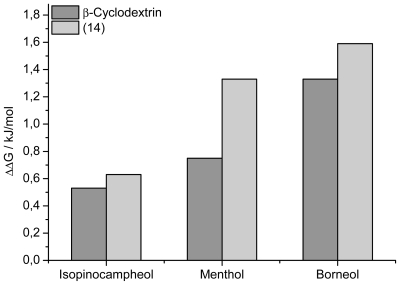
Comparison of the ΔΔ*G* values of enantiomeric pairs of guests for the complexation with with β-cyclodextrin and with cyclodextrin dimer **14** obtained by isothermal titration calorimetry.

In spite of the unfortunate design of dimer **14**, the ITC data consistently reveal a modest yet significant increase in chiral recognition. To evaluate the chiral recognition the ΔΔ*G* for interaction of the chiral guest pairs with β-CD and dimer **14** were plotted in a column diagram (Figure 4). The selectivity increases from ΔΔ*G* = 0.5–1.4 kJ mol^−1^ for the monovalent interaction to ΔΔ*G* = 0.6–1.6 kJ mol^−1^ for the interaction with the dimer. Hence, a modest degree of amplification of chiral recognition is obtained even if not all CD cavities are available for complexation. This result suggests that chiral recognition can be enhanced by multivalent interaction.

## 3. Conclusions

A new class of water soluble chiral guest dimers based on the (+) and (−) enantiomers of borneol, menthol and isopinocampheol was synthesized. In addition, a CD dimer was prepared. The formation of divalent inclusion complexes between the guest dimers and the CD dimer was measured by using ITC. The data obtained were compared with the monovalent interaction of the guest dimers with unmodified β-CD. It was found that the dimer largely isomerizes into a pseudorotaxane that preferentially binds in a monovalent rather than a divalent fashion. Nevertheless, it was shown that chiral recognition is amplified even if a substantial fraction of the cavities of the CD dimer are not available for complexation. In retrospect, it is obvious that chiral recognition can be significantly improved by a better choice of the CD dimer, for example by using a hydrophilic linker. Furthermore, it is anticipated that higher order multivalent interactions (e.g., of trimers and polymers) will show much higher enantioselectivities.

## Supplementary Materials



## Figures and Tables

**Table 1:** Overview of the thermodynamic parameters obtained by ITC.

Nr.	Guest (G)		Host (H)	Δ*H* (kJ mol^−1^)	Δ*G* (kJ mol^−1^)	Δ*S* (JK^−1^mol^−1^)	*K*_a_ (M^−1^) ×10^4^	*n* (H/G)	*C*_eff_ (mM)
**A**	**7a**	(+)Iso_2_	β −	−13.16	−24.01	36.70	1.68	1.06	
**B**	**7a**	(+)Iso_2_	**14**	−45.56	−27.69	−27.69	7.14	0.77	0.126
**C**	**7b**	(−)Iso_2_	β −	−14.90	−23.48	28.81	1.31	0.97	
**D**	**7b**	(−)Iso_2_	**14**	−34.50	−28.32	−28.32	9.22	0.80	0.268

**E**	**7c**	(−)Men_2_	β −	−14.22	−22.31	27.24	0.83	0.92	
**F**	**7c**	(−)Men_2_	**14**	−42.63	−25.65	−57.00	3.14	0.69	0.228
**G**	**7d**	(+)Men_2_	β −	−9.18	−23.06	46.57	1.10	1.03	
**H**	**7d**	(+)Men_2_	**14**	−25.04	−26.98	6.51	5.35	0.81	0.220

**I**	**7e**	(−)Bor_2_	β −	−18.89	−23.21	14.66	1.28	0.99	
**J**	**7e**	(−)Bor_2_	**14**	−41.58	−27.22	−48.19	5.90	0.77	0.168
**K**	**7f**	(+)Bor_2_	β −	−19.02	−24.54	18.51	2.00	0.93	
**L**	**7f**	(+)Bor_2_	**14**	−40.67	−28.81	−39.80	11.21	0.80	0.140

## References

[1] Bressolle F, Autran M, Pham TN, Vallon JJ (1996). Cyclodextrins and enantioselective separations of drugs by liquid chromatography and capillary electrophoresis: Basic principles and new developments. J. Chromatogr. B.

[2] Rekharsky M, Inoue Y (2000). Chiral Recognition Thermodynamics of β-Cyclodextrin: The Thermodynamic Origin of Enantioselectivity and the Enthalpy-Entropy Compensation Effect. J. Am. Chem. Soc.

[3] Schwarz-Barac S, Ritter H, Schollmeyer D (2003). Cyclodextrins in polymer synthesis: Enantiodiscrimination in free-radical polymerization of cyclodextrin-complexed racemic N-Methacryloyl-D, L-phenylalanine methyl ester. Macromol. Rapid Commun.

[4] D’Anna F, Riela S, Gruttadauria M, Lo Meo P, Noto R (2005). A spectrofluorimetric study of binary fluorophore-cyclodextrin complexes used as chiral selectors. Tetrahedron.

[5] Chen Y, Li F, Liu BW, Jiang BP, Zhang HY, Wang L, Liu Y (2010). Thermodynamic Origin of Selective Binding of β-Cyclodextrin Derivatives with Chiral Chromophoric Substituents toward Steroids. J. Phys. Chem. B.

[6] Gingter S, Bezdushna E, Ritter H (2010). Chiral Recognition of Poly(N-isopropylacrylamide-*co*-(D or L)-*N*-tryptophan-acrylamide) with Methylated β-Cyclodextrin. Macromolecules.

[7] Rekharsky M, Inoue Y (1998). Complexation Thermodynamics of Cyclodextrins. Chem. Rev.

[8] Liu Y, Zhang Q, Chen Y (2007). Spectrophotometric and Calorimetric Titration Studies on Molecular Recognition of Camphor and Borneol by Nucleobase-Modified β-Cyclodextrins. J. Phys. Chem. B.

[9] Mammen M, Choi SK, Whitesides GM (1998). Polyvalent interactions in biological systems: Implications for design and use of multivalent ligands and inhibitors. Angew. Chem. Int. Ed.

[10] Kim Y, Hechler B, Klutz AM, Gachet C, Jacobson KA (2008). Toward Multivalent Signaling across G Protein-Coupled Receptors from Poly(amidoamine) Dendrimers. Bioconjugate Chem.

[11] Liu Z, Deshazer H, Rice AJ, Chen K, Zhou C, Kallenbach NR (2006). Multivalent Antimicrobial Peptides from a Reactive Polymer Scaffold. J. Med. Chem.

[12] Bowman MC, Ballard TE, Ackerson CJ, Feldheim DL, Margolis DM, Melander C (2008). Inhibition of HIV Fusion with Multivalent Gold Nanoparticles. J. Am. Chem. Soc.

[13] DeFrees SA, Phillips L, Guo L, Zalipsky S (1996). Sialyl Lewis x Liposomes as a multivalent Ligand and Inhibitor of E-Selectin Mediated Cellular Adhesion. J. Am. Chem. Soc.

[14] Voskuhl J, Stuart MCA, Ravoo BJ (2010). Sugar-Decorated Sugar Vesicles: Lectin-Carbohydrate Recognition at the Surface of Cyclodextrin Vesicles. Chem. Eur. J.

[15] Crespo-Biel O, Lim CW, Ravoo BJ, Reinhoudt DN, Huskens J (2006). Expression of a supramolecular complex at a multivalent interface. J. Am. Chem. Soc.

[16] Harada A, Furue M, Nozakura SI (1980). Cooperative Binding by Cyclodextrin Dimers. Polym. J.

[17] Zhang B, Breslow R (1993). Enthalpic domination of the chelate effect in cyclodextrin dimers. J. Am. Chem. Soc.

[18] Jiang T, Sukumaran DK, Soni SD, Lawrence DS (1994). The Synthesis and Characterization of a Pyridine-Linked Cyclodextrin Dimer. J. Org. Chem.

[19] Breslow R, Zhang B (1996). Cholesterol Recognition and Binding by Cyclodextrin Dimers. J. Am. Chem. Soc.

[20] Haskard CA, Easton CJ, May BL, Lincoln SF (1996). Cooperative Binding of 6-(*p*-Toluidinyl) naphthalene-2-sulfonate by β-Cyclodextrin Dimers. J. Phys. Chem. B.

[21] Zhang B, Breslow R (1997). Ester Hydrolysis by a Catalytic Cyclodextrin Dimer Enzyme Mimic with a Metallobipyridyl Linking Group. J. Am. Chem. Soc.

[22] French RR, Holzer P, Leuenberger MG, Woggon WDA (2000). Supramolecular Enzyme Mimic That Catalyzes the 15,15′ Double Bond Scission of *β*,*β*-Carotene. Angew. Chem. Int. Ed.

[23] De Jong MR, Engbersen JFJ, Huskens J, Reinhoudt DN (2000). Cyclodextrin dimers as receptor molecules for steroid sensors. Chem. Eur. J.

[24] Edwards WB, Reichert DE, d’Avignon DA, Welch MJ (2001). β-Cyclodextrin dimers as potential tumor pretargeting agents. Chem Commun.

[25] Mulder A, Auletta T, Sartori A, Del Ciotto S, Casnati A, Ungaro R, Huskens J, Reinhoudt DN (2004). Divalent Binding of a Bis(adamantyl)-Functionalized Calix[4]arene to β-cyclodextrin-based Hosts: An Experimental and Theoretical Study on Multivalent Binding in Solution and at Self-Assembled Monolayers. J. Am. Chem. Soc.

[26] Liu Y, Ding F (2005). Molecular Recognition Thermodynamics of Bile Salts by β-Cyclodextrin Dimers: Factors Governing the Cooperative Binding of Cyclodextrin Dimers. J. Phys. Chem. B.

[27] Bea I, Kollman P (2006). Chelate Effect in Cyclodextrin Dimers: A Computational (MD, MM/PBSA, and MM/GBSA) Study. J. Org. Chem.

[28] Ohga K, Harada A (2005). Preparation of Supramolecular Polymers from a Cyclodextrin Dimer and Ditopic Guest Molecules: Control of Structure by Linker Flexibility. Macromolecules.

[29] Hasegawa Y, Harada A (2005). Supramolecular Polymers Formed from β-Cyclodextrins Dimer Linked by Poly (ethylene glycol) and Guest Dimers. Macromolecules.

[30] Leggio C, Tato J (2007). Study on the Structure of Host-Guest Supramolecular Polymers. Macromolecules.

[31] Mulder A, Jukovic A, Lucas LN, van Esch J, Feringa BL, Huskens J, Reinhoudt DN (2002). A dithienylethene-tethered beta-cyclodextrin dimer as a photoswitchable host. Chem Commun.

[32] Kuad P, Harada A (2007). External Stimulus-Responsive Supramolecular Structures Formed by a Stilbene Cyclodextrin Dimer. J. Am. Chem. Soc.

[33] Mourer M, Hapiot F, Monflier E, Menuel S (2008). Click chemistry as an efficient tool to access β-cyclodextrin dimers. Tetrahedron.

[34] Menuel S, Azaroual N, Landy D, Six N, Hapiot F, Monflier E (2011). Unusual Inversion Phenomenon of β-Cyclodextrin Dimers in Water. Chem. Eur. J.

