# Supplementary material for: Enhanced Chiral Recognition by Cyclodextrin Dimers
Source: Int J Mol Sci. 2011 Jul 18;12(7):4637–46. doi: 10.3390/ijms12074637 (PMC3155374; doi:10.3390/ijms12074637)
Supplement: Supplementary file 1 [file ijms-12-04637-s001.pdf]

Supporting Information

Jens Voskuhl, Kira Schaepe and Bart Jan Ravoo *

Organic Chemistry Institute, Westfälische Wilhelms-Universität Münster, Correnstrasse 40, Münster 48149, Germany; E-Mail: jens.voskuhl@uni-muenster.de

* Author to whom correspondence should be addressed; E-Mail: b.j.ravoo@uni-muenster.de; Tel.: +49-251-83-33287; Fax: +49-251-83-36557.

Received: 16 June 2011; in revised form: 7 July 2011 / Accepted: 8 July 2011 / Published: 18 July 2011

Abstract: In this article we investigate the effect of multivalency in chiral recognition. To this end, we measured the host-guest interaction of a β-cyclodextrin dimer with divalent chiral guests. We report the synthesis of carbohydrate-based water soluble chiral guests functionalized with two borneol, menthol, or isopinocampheol units in either (+) or (–) configuration. We determined the interaction of these divalent guests with a β-cyclodextrin dimer using isothermal titration calorimetry. It was found that—in spite of a highly unfavorable conformation—the cyclodextrin dimer binds to guest dimers with an increased enantioselectivity, which clearly reflects the effect of multivalency.

Keywords: cyclodextrins; chiral recognition; host-guest complexes; isothermal titration calorimetry; multivalency

1. General Procedures

Chemicals were purchased from Sigma Aldrich or from Acros Organics and used without further purification,Methanol and dimethylformamide (DMF) were dried by storage over molecular sieves (3 Å) for more than 3 days. Reactions were monitored by thin-layer chromatography (TLC), which was performed on 0.2 mm Merck precoated silica gel 60 F254 aluminum sheets. Spots were visualized by treatment with basic $KMnO_4$ solution. Column chromatography was carried out on silica gel 60 (0.063-0.2 mm, Merck). NMR spectra were recorded on Bruker spectrometers (AV300, AV400). Chemical shifts are given in units of parts per million (ppm) and expressed relative to the signals of deuterated solvents. Coupling constants (*J*) are reported in Hertz (Hz). Mass spectra were recorded with a MicroToF spectrometer (Bruker).

Isothermal titration calorimetry (ITC) was performed by using a Nano-Isothermal Titration calorimeter III (model CSC 5300; Calorimetry Sciences Corporation, London, Utah, USA). ITC measurements were performed in milli-Q water using a guest-host ration of 10 :1. 20 injections with a volume of 10 µl were performed.

1.1. General Procedure 1—Williamson ether Synthesis (GP1) [1]

The corresponding secondary alcohol was dissolved in dioxane under an atmosphere of argon. After that, NaH was added and the reaction mixture was heated to 100 °C for 10 minutes, followed by dropwise addition of chloroacetic acid in dioxane. The mixture was stirred for 12 h under reflux and finally treated with toluene and water. The organic layer was extracted three times with water and the combined aqueous layers were collected. After acidifying the aqueous layer with HCl to pH 2 the layer was extracted with toluene three times. The organic layer was dried over $MgSO_4$ followed by removal of the solvents *in vacuo*.

1.2. General Procedure 2—Peptide Coupling of Carboxylic Acid Derivatives to 4 (GP2)

To the diamine **4** was added the chiral carboxylic acid, EDCI and HOBt in 10 mL of dry DMF. After stirring for 10 minutes at room temperature NMM was added. After stirring the reaction mixture for 48 h, the solvent was removed *in vacuo* and the residue was dissolved in 20 mL of chloroform followed by washing with brine and saturated $NaHCO_3$-solution. After drying the organic layer over $MgSO_4$ and evaporating the solvent in vacuo the residue was applied to column chromatography ($CHCl_3$/ MeOH/ NH_3OH 9:1:0.1) and the desired product was obtained.

1.3. General Procedure 3—Cu(I) Catalyzed Click Reaction (GP3)

To the corresponding chiral dimer was added **2** in *t*-BuOH together with a catalytical amount of $CuSO_4$ and Na-ascorbate in distilled water. After 24 h stirring at room temperature the reaction mixture was diluted with water and extracted three times with 10 mL of CH_2Cl_2. The organic layers were collected and dried over $MgSO_4$. After removal of the solvent *in vacuo*, the crude residue was purified via column chromatography on SiO_2, using $CHCl_3$/MeOH 9:1 as eluent.

1.4. General Procedure 4—Deacetylation (GP4)

The corresponding chiral dimer with protected maltose unit was dissolved in 3 mL of methanol. After addition of a catalytic amount of NaOMe (10 mg) and stirring for 2 h at room temperature the solution was neutralized using ion exchange resin (Dowex HCR 20). The mixture was stirred for 20 minutes and after filtration the solvent was evaporated and the resulting solid was dried in vacuo.

2. Synthesis

2.1. Dimethyl 3,3'-(Prop-2-yne-1-ylazenediyl)dipropanoate (3) [2]

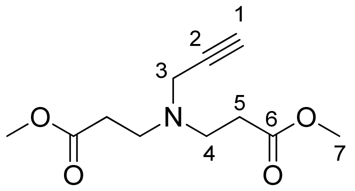

To an ice cooled and stirred solution of 20 mL acrylic acid methylester in 20 mL of MeOH was added propargylamine (2.00 g, 36 mmol) in 5 mL of MeOH. The resulting clear solution was stirred

for 3 days at room temperature. After that time the solvent was evaporated and the resulting residue was purified via column chromatography (EtOAc/pentane 1:1, Rf = 0.55).

Molecular formula: $C_{11}H_{17}NO_4$ (colorless oil).

Yield: 5.22 g (23 mmol, 64%).

^{1}H-NMR (300 MHz, $CDCl_3$, 298 K): δ = 2.20 (t, J = 2.4 Hz, 1H, 1-H), 2.47 (t, J = 7.1 Hz, 4H, 4-H), 2.84 (t, J = 7.1 Hz, 4H, 5-H), 3.43 (d, J = 2.4 Hz, 2H, 3-H), 3.67 (s, 6H, 7-H) ppm.

^{13}C-NMR (75 MHz, $CDCl_3$, 298 K): δ = 32.85 (CH_2, 5-C), 41.89 (CH_2, 3-C), 48.92 (CH_2, 4-C), 51.60 (CH_3, 7-C), 73.34 (CH, 1-C), 77.93 (C_q, 2-C), 171.11 (C_q, 6-C) ppm.

IR (neat) ν [cm^{-1}]: = 660 (m), 808 (w), 843 (w), 900 (w), 995 (w), 1045 (m), 1126 (m), 1172 (s), 1196 (s), 1259 (m), 1332 (w), 1361 (w), 1437 (m), 1732 (s), 2846 (w), 2954 (w), 3274 (br).

HRMS-ESI (m/z): Calculated for $[C_{11}H_{17}NO_4Na]^+$: 250.1050, found: 250.1050.

2.2. 3,3'-(prop-2-yn-1-ylazanediyl)bis(N-(2-aminoethyl)propanamide) (4) [2]

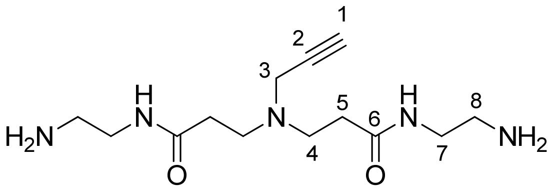

To a stirred and ice cooled solution of 35 mL ethylenediamine was added **3** (610 mg, 2.68 mmol, 1.0 eq.) in 2 mL of MeOH over a period of 10 minutes. The resulting mixture was stirred for 5 days at room temperature. After removal of all solvents the product was obtained and was used without further purification.

Molecular formula: $C_{13}H_{25}N_5O_2$ (yellow oil).

Yield: 682 mg (2.41 mmol, 90 %).

^{1}H-NMR (300 MHz, CD_3OD, 298 K): δ = 2.37 (t, J = 6.7 Hz, 4H, 5-H), 2.71 (t, J = 6.3 Hz, 5H, 1,7-H), 2.81 (t, J = 6.8 Hz, 4H, 4-H), 3.24 (t, J = 6.3 Hz, 4H, 8-H), 3.45 (s, 2H, 3-H) ppm.

^{13}C-NMR (75 MHz, CD_3OD, 298 K): δ = 35.73 (CH_2, 5-C), 42.85, 43.03 ($2CH_2$, 7,8-C), 43.82 (CH_2, 3-C), 51.46 (CH_2, 4-C), 75.91 (CH, 1-C), 79.53 (C_q, 2-C), 175.84 (C_q, 6-C) ppm.

IR (neat) ν [cm^{-1}]: = 598 (w), 660 (w), 900 (w), 1029 (w), 1131 (m), 1193 (w), 1267 (w), 1332 (w), 1360 (w), 1434 (w), 1459 (w), 1546 (s), 1639 (s), 2856 (br), 2929 (br), 3067 (br), 3285 (br).

HRMS-ESI (m/z): Calculated for $[C_{13}H_{25}N_5O_2H]^+$: 284.2081, found: 284.2081.

2.3. β-D-Glucopyranose-4-O-(2,3,4,6-tetra-O-acetyl-α-D-glucopyranosyl)-1,2,3,6-tetraacetate [3]

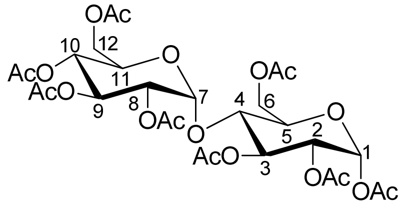

Maltose (3.01 g, 8.79 mmol) and I_2 (170 mg, cat.) were poured into a round bottom flask and were suspended with 50 mL of Ac_2O. After 50 minutes a concentrated mixture of sodium-thiosulfate in water to the brown solution was added. After extraction of the aqueous layer with CH_2Cl_2 (3 x 20 mL) and drying over $MgSO_4$ the product was obtained.

Molecular formula: $C_{28}H_{38}O_{19}$ (colorless solid).

Yield: 5.99 g (8.75 mmol, 99%).

^{1}H-NMR (300 MHz, $CDCl_3$, 298 K): δ = 1.97, 1.99, 2.00, 2.01, 2.05, 2.08, 2.12, 2.20 (8 s, 24H, OAc), 3.89-3.95 (m, 1H, 11-H), 3.99-4.13 (m, 3H, 4,5,6-H), 4.17-4.26 (m, 2H, 7-H,6-H\`), 4.44 (dd, 1H, $J$ = 2.2 Hz, $J$ = 12.4 Hz, 12-H), 4.85 (dd, 1H, $J$ = 4.0 Hz, $J$ = 10.6 Hz, 12-H\`), 4.94 (dd, 1H, J = 3.7 Hz, J = 10.2 Hz, 2-H), 5.06 (t, 1H, J = 9.9 Hz, 9/10-H), 5.35 (d, 1H, J = 10.5 Hz, 7-H), 5.41 (t, 1H, J = 4.0 Hz, 3-H), 5.49 (dd, 1H, J = 4.4 Hz, J = 4.5 Hz, 8-H), 6.22 (d, 1H, J = 3.7 Hz, 1-H) ppm.

^{13}C-NMR (75 MHz, $CDCl_3$, 298 K): δ = 20.53-22.16 (8 CH_3, OAc), 61.40 (CH_2, 6-C), 62.48 (CH_2, 12-C), 67.90-72.94 (8 CH, 2,3,4,5,8,9,10,11-C), 91.22 (CH, 7-C), 95.68 (CH,1-C), 166.39-170.55 (8C_q, OAc) ppm.

IR (neat) ν [cm^{-1}]: = 627 (w), 741 (w), 751 (w), 768 (w), 792 (w), 901 (m), 948 (m), 1061 (s), 1231 (s), 1408 (m), 1472 (w), 1601 (w), 1755 (s), 2347 (m), 2363 (m).

HRMS-ESI (m/z): Calculated for $[C_{28}H_{38}O_{19}Na]^+$: 701.1900, found: 701.1898.

Specific rotation: $[\alpha]^{20}_D$ = + 56.8° (c = 1.1, $CHCl_3$).

Melting point: 85 °C (CH_2Cl_2).

2.4. β-D-Glucopyranosyl-azide-4-O-(2,3,4,6-teta-O-acetyl-β-D-glucopyranosyl)-2,3,6-tiacetate (2) [4]

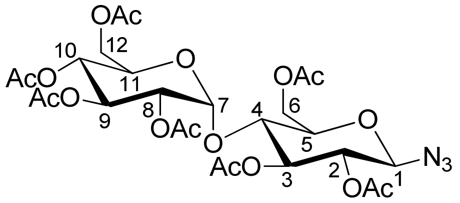

To the peracetylated maltose (3.00 g, 4.42 mmol) dissolved in 20 mL of CH_2Cl_2 was added under an atmosphere of argon $SiMe_3N_3$ (0.85 mL, 9.0 mmol) and $SnCl_4$ (0.4 mL, 3.0 mmol). The resulting mixture was stirred for 18 h at room temperature. After that time the mixture was diluted with saturated $NaHCO_3$ solution and was extracted 3 times with 20 mL of CH_2Cl_2. The combined organic layers were dried over $MgSO_4$ and the solvent was removed *in vacuo*.

Molecular formula: $C_{26}H_{35}N_3O_{17}$ (white solid).

Yield: 2.62 g, (3.96 mmol, 90 %).

^{1}H-NMR (300 MHz, $CDCl_3$, 298 K): δ = 1.99, 2.00, 2.01, 2.03, 2.04, 2.09,2.14 (8 s, 24H, OAc), 3.74-3.80, 3.92-4.06 (m, 4H, 4,5,11,12-H), 4.20-4.26 (m, 2H, 6,12-H\`), 4.50 (dd, J = 12.2 Hz, J = 2.2 Hz, 6-H), 4.71 (t, 1H, J = 8.7 Hz, 3-H), 4.77 (d, 1H, J = 8.8 Hz, 1-H), 4.84 (dd, J = 10.5 Hz, J = 4.0 Hz, 8-H), 5.04 (t, 1H, J = 9.9 Hz, 30-H), 5.25 (t, 1H, J = 8.9 Hz, 2-H), 5.34 (t, 1H, J = 10.0 Hz, 9-H), 5.40 (d, 1H, J = 4.0 Hz, 7-H) ppm.

^{13}C-NMR (75 MHz, $CDCl_3$, 298 K): δ = 20.88, 20.90, 20.92, 21.01, 21.11, 21.17 (7 CH_3, OAc), 62.82, 61.75 (2 CH_2, 6,12-C), 68.23, 68.93, 69.55, 70.28, 71.78, 72.61, 74.53, 75.39 (8 CH_2, 2,3,4,5,8,9,10,11-C), 89.77 (CH, 1-C), 96.00 (CH, 7-C), 169.74, 169.82, 170.26, 170.43, 170.74, 170.84 (7 C_q, OAc) ppm.

IR (neat) ν [cm^{-1}]: = 687 (w), 798 (m), 897 (w), 938 (w), 1026 (s), 1216 (s), 1368 (m), 1434 (w), 1742 (s), 2120 (m), 2341 (w), 2360 (w), 2960 (br).

HRMS-ESI (m/z): Calculated for $[C_{26}H_{35}N_3O_{17}Na]^+$: 684.1859, found: 684.1856.

Specific rotation: $[\alpha]^{20}_D$ = + 41.1° (c = 1.0, MeOH).

Melting point: 79 °C (CH_2Cl_2).

2.5. 1,3-bis(prop-2-yn-1-yloxy)benzene (12) [5]

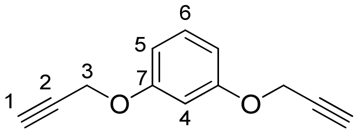

To a stirred solution of resorcinol (1.0 g, 9.1 mmol) in 20 mL of dry DMF was added K_2CO_3 (4.1 g, 30 mmol). After 30 minutes propargylbromide (2.32 g, 24.4 mmol) was added dropwise to the suspension and the mixture was stirred for additional 7 h at room temperature. After that time the mixture was washed with distilled water and extacted with $CHCl_3$ (3 x 20 mL). The combined organic layers were dried over $MgSO_4$ and evaporated *in vacuo*. The residue was purified via column chromatography (EtOAc / pentane, 1:5, Rf = 0.4).

Molecular formula: $C_{12}H_{10}O_2$ (colorless solid).

Yield: 1.54 g, (8.00 mmol, 91 %).

^{1}H-NMR (300 MHz, $CDCl_3$, 298 K): δ = 2.29 (t, J = 2.4 Hz, 2H, 1-H), 4.44 (d, J = 2.4 Hz, 4H, 2-H), 6.30-6.48 (m, 3H, 4,5-H), 6.88-7.02 (m, 1H, 6-H) ppm.

^{13}C-NMR (75 MHz, $CDCl_3$, 298 K): δ = 55.85 (CH_2, 3-C), 75.62 (CH, 1-C), 78.42 (C_q, 2-C), 102.40 (CH, 4-C), 107.86 (CH, 5-C), 129.97 (CH, 6-C), 158.71 (C_q,7-C) ppm.

IR (neat) ν [cm^{-1}]: = 627 (s), 680 (s), 749 (s), 936 (m), 1046 (s), 1141 (s), 1186 (w), 1246 (w), 1291 (m), 1327 (w), 1360 (w), 1456 (w), 1492 (m), 1587 (m), 1614 (m), 3552 (s), 3286 (m).

HRMS-ESI (m/z): Calculated for $[C_{12}H_{10}O_2Na]^+$: 209.0578, found: 209.0611.

2.6. Mono-6-deoxy-6-(p-tolylsulfonyl)-β-cyclodextrin (9) [6]

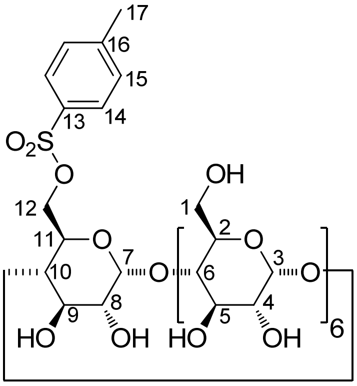

To a stirred and ice - cooled solution of β-cyclodextrin (10 g, 8.8 mmol) and NaOH (5 g, 12.5 mmol) in 300 mL of water was added *p*-toluenesulfonylchloride (4 g, 13.4 mmol). The reaction mixture was stirred for 2 h at 0 °C. After that time another portion of p-Toluolsulfonylchloride (6 g, 20 mmol) was added to the solution. The mixture was stirred for additional 3 h at 0°C and followed by filtering. The obtained clear solution was acidified with conc. HCl to pH 6. The colorless precipitate was filtered off and recrystallized 3 times from water.

Molecular formula: $C_{49}H_{76}O_{37}S$ (white solid).

Yield: 3.71 g (2.93 mmol, 31 %).

^{1}H-NMR (DMSO-d_6, 298 K): δ = 2.49 (s, 3H, 17-H), 3.20-3.42 (m, 14H, 4,6,8,10-H), 3.50-3.80 (m, 28H, 1,2,5,9,11,12-H), 4.18-4.70(m, 7H, 5,9-H), 4.77-4.90 (m, 7H, 3,7-H), 5.67-5.98(m, 14H, 14-OH), 7.51 (d, 2H, 15-H), 7.80 (d, 2H, 14-H).

^{13}C-NMR (DMSO-d_6, 298 K): δ = 21.23 (CH_3, 17-C), 59.33 (CH_2, 12-C), 59.89 (CH2, 1-C), 72.25-72.95 (CH, 2,4,5,8,9,11-C), 81.21 (CH, 6,10-C), 101.45 (CH, 3,7-C).

IR (neat) ν [cm^{-1}]: = 668 (w), 706 (w), 755 (w), 817 (w), 835 (w), 937 (w), 968 (w), 1004 (s), 1023 (s), 1047 (s), 1079 (s), 1156 (m), 1178 (w), 1306 (w), 1361 (w), 1408 (w), 1644 (w), 2904 (br), 3260 (br).

HRMS-ESI (m/z): Calculated for $[C_{49}H_{76}O_{37}SNa]^+$, found: 1311.2511.

Specific rotation: $[\alpha]^{20}_D$ = + 117.5° (c = 1.1, DMSO).

Melting point: 161 °C (H_2O).

2.7. Mono-6-deoxy-6-azido-β-cyclodextrin (10) [7]

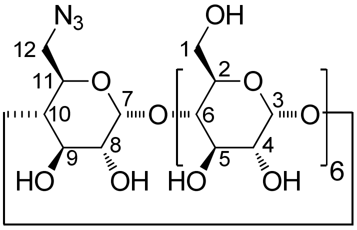

β-cyclodextrin monotosylate **(9)** (3.7 g, 2.9 mmol) was dissolved in 100 mL of water. After addition of sodium azide the reaction mixture was heated to reflux for 24 h. To the clear solution was then

added 50 mL of acetone and the flask was stored in a fridge at 4 °C over night. The obtained precipitate was filtered off, washed with cold acetone and dried *in vacuo*.

Molecular formula: $C_{42}H_{69}N_3O_{34}$ (white solid).

Yield: 2.7 g (2.4 mmol, 83 %).

^{1}H-NMR (300 MHz, DMSO-*d6,* 298K): δ = 3.25-3.53 (m, 14H, 4,6,8,10-H), 3.55-3.90 (m, 28H, 1,2,5,9,11,12-H), 4.50-4.69 (m, 7H, 5,9-H), 4.82-4.99 (m, 7H, 3,7-H), 5.68-5.97 (m, 14H,14-OH).

^{13}C NMR (75 MHz, DMSO-*d6*, 298K): δ = 51.15 (CH_2, 12-C), 59.99 (CH_2, 1-C), 70.27-73.13 (CH, 4,5,6,8,9,10-C), 81.61 (CH, 11-C) , 83.06 (CH, 2-C), 102.01 (CH, 3-C), 102.36 (CH, 7-C).

IR (neat) ν [cm^{-1}]: = 573 (w), 609 (w), 669 (w), 754 (w), 939 (w), 1003 (m), 1027 (s), 1080 (m), 1155 (m), 1298 (w), 1330 (w), 1367 (w), 2037 (w), 2101 (w), 2342 (m), 2361 (br).

HRMS-ESI (m/z): Calculated for $[C_{42}H_{69}N_3O_{34}N]^+$: 1182.3655, found: 1182.3682.

Specific rotation: $[\alpha]^{20}_{D}$ = + 123.7° (c = 1.45, DMSO).

Melting point: 251 °C (H_2O).

2.8. Icosa-O-acetyl-6-azido-β-cyclodextrin (11) [8]

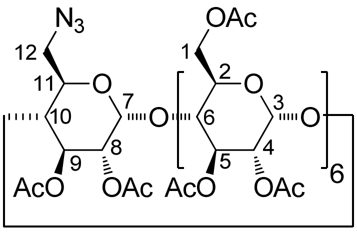

β-cyclodextrin monoazide **(10)** (2.2 g, 19 mmol) was dissolved in 45 mL of dry pyridine. To that solution was added 54 mL acetic anhydride. The solution was stirred for 18 h a room temperature and then quenched upon addition of methanol at 0 °C. All solvents were removed *in vacuo* and redissolved in 30 mL of CH_2Cl_2. The organic layer was extracted once with 20 mL of 1M HCl, saturated $NaHCO_3$ and saturated NaCl solution. The collected organic layers were dried over $MgSO_4$ and the solvent was removed *in vacuo*.

Molecular formula: $C_{82}H_{109}N_3O_{54}$ (white solid).

Yield: 3.2 g (16 mmol, 84 %).

^{1}H-NMR (300 MHz, $CDCl_3$, 298K): δ = 2.17-1.84 (m, 69H, OAc), 3.79-3.56 (m, 9H, 6,10,12-*H*), 4.30-3.95 (m, 7H, 2,11-*H*), 4.50 (dd, *J* = 19.1, 8.4 Hz, 12H, 1-*H*), 4.81-4.65 (m, 7H, 4,8-*H*), 4.91-5.10 (m, 7H, 3,7-*H*), 5.31-5.11 (m, 7H, 5,9-*H*) ppm.

^{13}C-NMR (75 MHz, $CDCl_3$, 298K): δ = 19.85 (CH3, OAc), 52.38 (CH2, 3,7-*C*), 61.37 (CH2, 12-*C*), 61.47 (CH, 2,11-*C*), 68.55 (CH, 4,8-*C*), 69.69 (CH, 5,9-*C*), 70.05 (CH, 6,10-*C*) 95.79 (CH2, 1-*H*), 169.45 (Cq, OAc), 168.41 (Cq, OAc) ppm.

IR (neat) ν [cm^{-1}]: = 603 (m), 654 (w), 704 (w), 797 (m), 900 (m), 958 (w), 1025 (s), 1209 (s), 1370 (m), 1433 (w), 1741 (s), 2104 (br), 2285 (w), 2349 (m), 2399 (w), 2965 (br).

HRMS-ESI (m/z): Calculated for $[C_{82}H_{109}N_3O_{54}Na_2]^{2+}$: 1022.7830, found: 1022.7807.

Specific rotation: + 143.65° (c = 0.95, MeOH).

Melting point: 158-160 °C (CH_2Cl_2).

2.9. Icosa-O-acetyl-6-triazol-β-cyclodextrindimer (13)

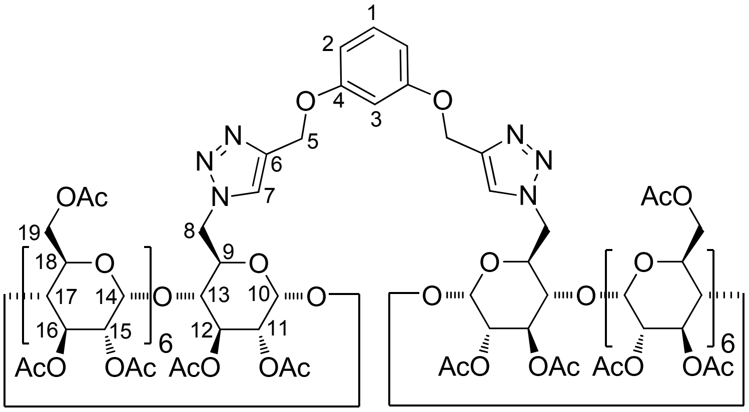

To a solution of the dialkyne **12** (23 mg, 0.12 mmol) in 10 mL of DMF, the protected β-cyclodextin monoazide **11** (500 mg, 0.25 mmol) was added. To this solution a freshly prepared mixture of $CuSO_4$ (10 mg, cat.) and Na-ascorbate (20 mg, cat.) in distilled water was added. The solution was stirred for 24 h at room temperature. Afterwards the solvent was removed *in vacuo* and the residue was redissolved in CH_2Cl_2. The organic layer was then extracted with distilled water (2 x 20 mL) and brine (2 x 20 mL). After drying the organic layer over MgSO4 and removal of the solvent *in vacuo* the crude product was purified via column chromatography (EtOAc/acetone 3:1, R_f = 0.38).

Molecular formula: $C_{175}H_{226}N_6O_{110}$ (white solid).

Yield: 0.36 g (0.08 mmol, 60 %).

^{1}H-NMR ($CDCl_3$, 300 MHz, 298 K): δ = 1.85 – 2.18 (m, 105H, 35-OAc), 3.42 – 3.66 (m, 16H, 8,10,13-H), 4.16 (m, 26H, 8,9,14,19-H), 4.50-4.77 (m, 26H, 11,15,19-H), 5.00-5.27 (m, 30H, 5,12,14,16-H), 5.58 (d, 2H, 10-H), 6.63 (dd, J = 2.2, 8.2 Hz, 2H, 2-H), 6.68 (t, J = 2.2 Hz, 1H, ,3-H), 7.19 (t, J = 8.2 Hz, 1-H), 7.72 (s, 2H, 7-H) ppm.

^{13}C-NMR ($CDCl_3$, 75 MHz, 298 K): δ = 20.62-20.80 (CH_3, OAc), 49.26 (CH2, 13,17-C), 61.96-62.95 (CH, 5,11,12,15,16-C), 70.02-71.26 (CH, 10,14-C), 96.40-96.80 (CH_2, 19-C), 102.21 (CH, 3-C), 107.47 (CH, 2-C), 125.81 (CH, 7-C), 129.96 (CH, 1-C), 143.66 (C_q, 4-C), 159.54 (C_q, 4-C), 169.41-170.49 (C_q, OAc) ppm.

IR (neat) ν [cm^{-1}]: = 536 (m), 622 (m), 715 (w), 781 (w), 952 (w), 1004 (s), 1033 (m), 1210 (s), 1390 (s), 1757 (s), 2105 (m), 2924 (w), 3319 (w).

HRMS-ESI (m/z): Calculated for $[C_{176}H_{228}N_6O_{110}Na_2]^{2+}$:2115.0637, found: 2115.0619

Specific rotation: $[\alpha]^{20}_D$ = + 94.4° (c = 0.58, H_2O).

Melting point: 149 °C (EtOAc).

2.10. Icosa-hydroxy-6-triazol-β-cyclodextrindimer (14) [9]

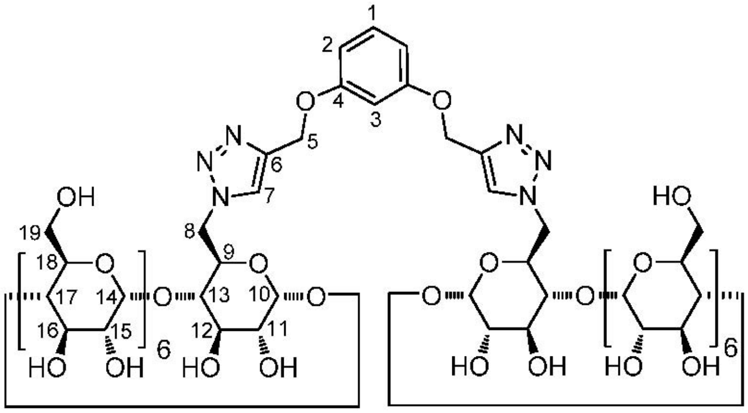

To a stirred solution of **(V-9)** (100 mg, 0.023 mmol) in 5 mL MeOH was added a catalytical amount of NaOMe (5 mg) in MeOH. The clear solution was stirred for 24 h followed by neutalisation with ion exchange resin (Dowex HCR 20). After filtration, the solvent was evaporated to dryness affording the desired compound **14**.

Molecular formula: $C_{96}H_{148}N_6O_{70}$ (colorless solid).

Yield: 44 mg (0.018 mmol, 78 %).

^{1}H-NMR ($CDCl_3$, 400 MHz, 298 K): δ = 3.03-3.29 (m, 1H, 9b-H), 3.39-3.74 (m, 28H, 12,13,16,17-H), 3.75-3.97 (m, 28 H, 10,11,14,15-H), 3.99-4.12 (m, 2H, 9,9a-H), 4.49-4.65 (m, 4H, 8,8a,8b-H), 4.89-5.17 (m, 28H, 19-H), 5.17-5.46 (m, 4H, 5,5a,5b-H), 6.19 (bs, 1H, 3a-H), 6.72-6.76 (m, 2H, 2a,2,3b-H), 6.89-6.92 (m, 1H, 2b-H), 7.18 (s, 1H, 7b-H), 7.34 (t, J = 8.3 Hz, 1H, 1-H), 7.86 (s, 1H, 7a), 8.11 (s, 1H, 7-H) ppm.

IR (neat) ν [cm^{-1}]: = 555 (m), 613 (m), 705 (w), 753 (w), 947 (w), 1000 (s), 1026 (s), 1102 (s), 1335 (w), 1410 (w), 1593 (m), 2924 (w), 3319 (w).

HRMS-ESI (m/z): Calculated for $[C_{96}H_{148}N_6O_{70}KH]^{2+}$:1272.8953, found: 1272.8953.

Specific rotation: $[\alpha]^{20}_D$ = + 149.7° (c = 0.155, H_2O).

Melting point: 252 °C decomp. (MeOH).

2.11. (+)-Isopinocampheyl-oxyacetic acid (1a)

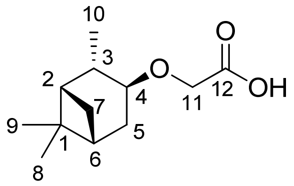

According to **GP1** (+)-isopinocampheol (2.00 g, 13.0 mmol, 1.0 eq.) was dissolved in 50 mL of dioxane and was treated with NaH (2.60 g, 65.0 mmol) and bromoacetic acid (3.61 g, 26.0 mmol, 2.0 eq.). After the described work up procedure the product was obtained.

Molecular formula: $C_{12}H_{20}O_3$ (yellow wax).

Yield: 2.57 g (12.10 mmol, 93%).

^{1}H-NMR (300 MHz, $CDCl_3$, 298 K): δ = 0.89 (s, 3H, 9-H), 1.05 (d, J = 9.8 Hz, 1H, 7-H), 1.14 (d, J = 7.4 Hz, 3H, 10-H), 1.21 (s, 3H, 8-H), 1.77-1.83 (m, 2H, 2,5-H), 1.92-1.98 (m, 1H, 6-H), 2.05-2.14 (m, 1H, 3-H), 2.30-2.47 (m, 2H, 5,7-H`), 3.74-3.80 (m, 1H, 4-H), 4.10 (d, $J$ = 16.7 Hz, 1H, 11-H), 4.20 (d, $J$ = 16.7 Hz, 1H, 11-H`), 10.15 (bs, 1H, OH).

^{13}C-NMR (75 MHz, $CDCl_3$, 298 K): δ = 21.49 (CH_3, 10-C), 24.09 (CH_3, 9-C), 27.73 (CH_3, 8-C), 33.69 (CH_2, 7-C), 35.45 (CH_2, 5-C), 38.68 (C_q, 1-C), 41.57 (CH, 6-C), 44.57 (CH, 3-C), 47.78 (CH, 2-C), 66.03 (CH_2, 11-C), 80.57 (CH, 4-C), 175.19 (C_q, 12-C).

IR (neat) ν [cm^{-1}]: = 613 (w), 646 (w), 682 (w), 779 (w), 812 (w), 858 (w), 885 (w), 920 (m), 970 (m), 1027 (w), 1056 (w), 1097 (s), 1155 (w), 1200 (s), 1262 (w), 1376 (w), 1431 (m), 1744 (s), 2915 (br).
HRMS-ESI (m/z): Calculated for $[C_{12}H_{19}O_3]^-$: 211.1329, found: 211.1351.

Specific rotation: $[\alpha]^{20}_{D}$ = + 61.2° (c = 0.92, $CHCl_3$).

2.12. (-)-Isopinocampheyl-oxy acetic acid (1b)

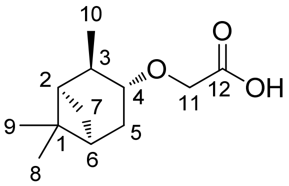

According to **GP1** (-)-isopinocampheol (2.00 g, 13.0 mmol, 1.0 eq.) was dissolved in 50 mL of dioxane and was treated with NaH (2.60 g, 65.0 mmol) and bromoacetic acid (3.61 g, 26.0 mmol, 2.0 eq.). After the described work up procedure the product was obtained.

Molecular formula: $C_{12}H_{20}O_3$ (yellow wax).

Yield: 2.01 g (9.01 mmol, 70%).

^{1}H-NMR (300 MHz, $CDCl_3$, 298 K): δ = 0.88 (s, 3H, 9-H), 1.05 (d, J = 9.8 Hz, 1H, 7-H), 1.13 (d, J = 7.4 Hz, 3H, 10-H), 1.20 (s, 3H, 8-H), 1.76-1.83 (m, 2H, 2,5-H), 1.91-1.97 (m, 1H, 6-H), 2.06-2.11 (m, 1H, 3-H), 2.29-2.47 (m, 2H, 5,7-H), 3.74-3.80 (m, 1H, 4-H), 4.10 (d, J = 16.7 Hz, 1H, 11-H), 4.10 (d, J = 16.7 Hz, 1H, 11-H`), 10.28 (bs, 1H, OH) ppm.

^{13}C-NMR (75 MHz, $CDCl_3$, 298 K): δ = 21.28 (CH_3, 10-C), 23.88 (CH_3, 9-C), 27.53 (CH_3, 8-C), 33.47 (CH_2, 7-C), 35.23 (CH_2, 5-C), 38.48 (C_q, 1-C), 41.37 (CH, 6-C), 44.35 (CH, 3-C), 47.58 (CH, 2-C), 65.80 (CH_2, 11-C), 80.31 (CH, 4-C), 175.25 (C_q, 12-C) ppm.

IR (neat) ν [cm^{-1}]: = 612 (w), 682 (s), 779 (m), 824 (w), 851 (w), 885 (w), 921 (s), 970 (m), 1027 (w), 1056 (w), 1097 (s), 1200 (s), 1269 (w), 1376 (w), 1432 (s), 1470 (m), 1648 (m), 1744 (s), 2914 (br).
HRMS-ESI (m/z): Calculated for $[C_{12}H_{19}O_3]^-$: 211.1329, found: 211.1356.

Specific rotation: $[\alpha]^{20}_{D}$ = - 60.6° (c = 1.05, $CHCl_3$).

2.13. (-)-Menthyl-oxyacetic acid (1c) [1]

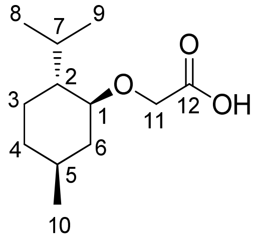

According to **GP1** (-)-menthol (2.00 g, 12.8 mmol, 1.0 eq.) was dissolved in 50 mL of dioxane and was treated with NaH (2.57 g, 64.0 mmol) and bromoacetic acid (3.56 g, 25.6 mmol, 2.0 eq.). After the described work up procedure the product was obtained.

Molecular formula: $C_{12}H_{22}O_3$ (brownish oil).

Yield: 1.57 g (7.34 mmol, 57%).

^{1}H-NMR (300 MHz, $CDCl_3$, 298 K): δ = 0.78 (d, J = 6.9 Hz, 3H, 9-H), 0.68-0.87 (m, 2H, 4,6-H), 0.90 (d, J = 6.7 Hz, 3H, 8-H), 0.92 (d, J = 6.4 Hz, 3H, 10-H), 0.94-1.04 (m, 1H, 3-H), 1.25-1.42 (m, 2H, 2,5-H), 1.62-1.67 (m, 2H, 3,4-H), 2.05-2.15 (m, J = 12.0 Hz, 1H, 6-H), 2.21 (dsep, J = 6.9 Hz, J = 2.4 Hz, 1H, 7-H), 3.21 (dt, J = 10.6 Hz, J = 4.1 Hz, 1H, 1-H), 4.07 (d, J = 16.7 Hz, 1H, 11-H), 4.20 (d, J = 16.7 Hz, 1H, 11-H), 9.44 (bs, 1H, O-H).

^{13}C-NMR (75 MHz, $CDCl_3$, 298 K): δ = 16.47 (CH_3, 9-C), 21.25 (CH_3, 8-C), 22.53 (CH_3, 10-C), 23.48 (CH_2, 3-C), 25.96 (CH, 7-C), 31.79 (CH, 5-C), 34.59 (CH_2, 4-C), 40.19 (CH_2, 6-C), 48.23 (CH, 2-C), 65.77 (CH_2, 11-C), 80.97 (CH, 1-H), 174.80 (C_q, 12-C).

IR (neat) ν [cm^{-1}]: = 688 (w), 730 (w), 801 (w), 844 (w), 911 (w), 956 (w), 984 (w), 1025 (w), 1041 (w), 1123 (s), 1369 (m), 1455 (m), 1734 (s), 2870 (m), 2922 (m), 2954 (m).

HRMS-ESI (m/z): Calculated for $[C_{12}H_{22}O_3]^-$: 213.1496, found: 213.1495.

Specific rotation: $[\alpha]^{20}_D$ = -84.1° (c = 0.99, $CHCl_3$).

2.14. (+)-Menthyl-oxyacetic acid (1d)

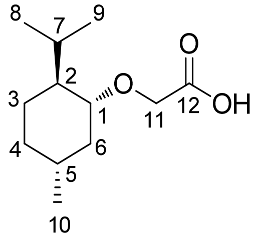

According to **GP1** (+)-menthol (2.00 g, 12.8 mmol, 1.0 eq.) was dissolved in 50 mL of dioxane and was treated with NaH (2.57 g, 64.0 mmol) and bromoacetic acid (3.56 g, 25.6 mmol, 2.0 eq.). After the described work up procedure the product was obtained.

Molecular formula: $C_{12}H_{22}O_3$ (brownish oil).

Yield: 1.95 g (8.74 mmol, 67 %).

^{1}H-NMR (300 MHz, $CDCl_3$, 298 K): δ = 0.79 (d, J = 6.9 Hz, 3H, 9-H), 0.68-0.87 (m, 2H, 4,6-H), 0.93 (d, J = 6.7 Hz, 3H, 8-H), 0.96 (d, J = 6.4 Hz, 3H, 10-H), 0.94-1.04 (m, 1H, 3-H), 1.27-1.41 (m, 2H, 2,5-H), 1.63-1.69 (m, 2H, 3,4-H), 2.00-2.09 (m, J = 12.0 Hz, 1H, 6-H), 2.16 (dsep, J = 6.9 Hz, J = 2.4 Hz, 1H, 7-H), 3.22 (dt, J = 10.6 Hz, J = 4.1 Hz, 1H, 1-H), 4.07 (d, J = 16.7 Hz, 1H, 11-H), 4.20 (d, J = 16.7 Hz, 1H, 11-H), 9.40 (bs, 1H, OH).

^{13}C-NMR (75 MHz, $CDCl_3$, 298 K): δ = 16.13 (CH_3, 9-C), 20.91 (CH_3, 8-C), 22.19 (CH_3, 10-C), 23.14 (CH_2, 3-C), 25.67 (CH, 7-C), 31.45 (CH, 5-C), 34.25 (CH_2, 4-C), 39.87 (CH_2, 6-C), 47.89 (CH, 2-C), 65.43 (CH_2, 11-C), 80.68 (CH, 1-H), 173.97 (C_q, 12-C).

IR (neat) ν [cm^{-1}]: = 672 (m), 845 (w), 880 (w), 911 (w), 958 (w), 972 (w), 1009 (w), 1122 (s), 1180 (m), 1236 (m), 1345 (w), 1386 (w), 1455 (m), 1646 (m), 1730 (s), 2870 (m), 2922 (m), 2954 (m).

HRMS-ESI (m/z): Calculated for $[C_{12}H_{22}O_3]^-$: 213.1485, found: 213.1488.

Specific rotation: $[\alpha]^{20}_{D}$ = + 85.2° (c = 1.01, $CHCl_3$).

2.15. (-) Borneyl-oxyacetic Acid (1e)

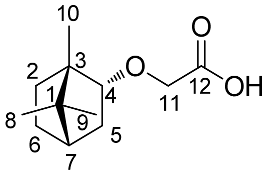

According to **GP1** (-)-borneol (1.00 g, 6.50 mmol, 1.0 eq.) was dissolved in 50 mL of dioxane and was teated with NaH (1.28 g, 32.0 mmol, 5.0 eq) and bromoacetic acid (1.78 g, 12.8 mmol, 2.0 eq.). After the described work up procedure the product was obtained.

Molecular formula: $C_{12}H_{20}O_3$ (brownish waxy solid).

Yield: 822 mg (3.68 mmol, 57 %).

^{1}H-NMR (300 MHz, $CDCl_3$, 298 K) δ = 0.84 (s, 3H, 9-H), 0.86 (s, 3H, 10-H), 0.92 (s, 3H, 8-H), 1.21-1.32 (m, 3H, 2,7-H), 1.66-1.76 (m, 2H, 6-H), 1.91-2.00 (m, 1H, 5-H), 2.12-2.21 (m, 1H, 5-H), 3.69-3.74 (m, 1H, 4-H), 4.06 (d, J = 16.7 Hz, 1H, 11-H), 4.14 (d, J = 16.7 Hz, 1H, 11-H), 9.09 (bs, 1H, OH).

^{13}C-NMR (75 MHz, $CDCl_3$, 298 K) δ = 13.92 (CH_3, 10-C), 18.78 (CH_3, 9-C), 19.70 (CH_3, 8-C), 26.50 (CH_2, 6-C), 28.10 (CH_2, 2-C), 35.76 (CH_2, 5-C), 44.80 (CH, 7-C), 48.03 (C_q, 3-C), 49.34 (C_q, 1-C), 86.59 (CH_2, 11-C), 174.29 (C_q,12-C).

IR (neat) ν [cm^{-1}]: = 692 (w), 748 (w), 821 (w), 882 (w), 918 (m), 986 (w), 1053 (w), 1131 (s), 1248 (s), 1302 (m), 1366 (w), 1388 (w), 1430 (m), 1453 (m), 1729 (s), 2876 (m), 2949 (s).

HRMS-ESI (m/z): Calculated for $[C_{12}H_{22}O_3]^-$: 213.1496, found: 213.1491.

Specific rotation: $[\alpha]^{20}_{D}$ = - 50.7° (c = 0.95, $CHCl_3$).

2.16. (+) Borneyl-oxyacetic acid (1f)

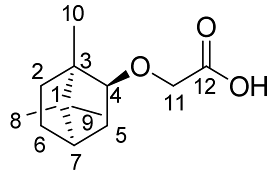

According to **GP1** (+)-borneol (1.00 g, 6.50 mmol, 1.0 eq.) was dissolved in 50 mL of dioxane and was teated with NaH (1.28 g, 32.0 mmol, 5.0 eq) and bromoacetic acid (1.78 g, 12.8 mmol, 2.0 eq.). After the described work up procedure the product was obtained.

Molecular formula: $C_{12}H_{20}O_3$ (brownish wax).

Yield: 1.11 g (5.42 mmol, 76 %).

^{1}H-NMR (300 MHz, CD_2Cl_2, 298 K): δ = 0.84 (s, 3H, 9-H), 0.86 (s, 3H, 10-H), 0.92 (s, 3H, 8-H), 1.21-1.32 (m, 3H, 2,7-H), 1.66-1.76 (m, 2H, 6-H), 1.91-2.00 (m, 1H, 5-H), 2.12-2.21 (m, 1H, 5-H), 3.69-3.74 (m, 1H, 4-H), 4.06 (d, J = 16.7 Hz, 1H, 11-H), 4.12 (d, J = 16.7 Hz, 1H, 11-H) ppm.

^{13}C-NMR (75 MHz, CD_2Cl_2, 298 K): δ = 14.09 (CH_3, 10-C), 18.96 (CH_3, 9-C), 19.89 (CH_3, 8-C), 26.96 (CH_2, 6-C), 28.50 (CH_2, 2-C), 36.15 (CH_2, 5-C), 45.39 (CH, 7-C), 48.41 (C_q, 3-C), 49.71 (C_q, 1-C), 86.97 (CH_2, 11-C), 172.98 (C_q,12-C) ppm.

IR (neat) ν [cm^{-1}]: = 693 (w), 699 (w), 746 (w), 821 (w), 882 (w), 918 (m), 986 (w), 1053 (w), 1131 (s), 1248 (s), 1312 (m), 1367 (w), 1388 (w), 1410 (m), 1431 (m), 1443 (m), 1730 (s), 2876 (m), 2949 (s).

HRMS-ESI (m/z): Calculated for $[C_{12}H_{22}O_3]^-$: 213.1496, found: 213.1491.

Specific rotation: $[\alpha]^{20}_D$ = + 50.8° (c = 1.00, $CHCl_3$).

2.17. (+)-Disopinocampheyl-oxy-acetoxyethylendiamine-alkyne (5a)

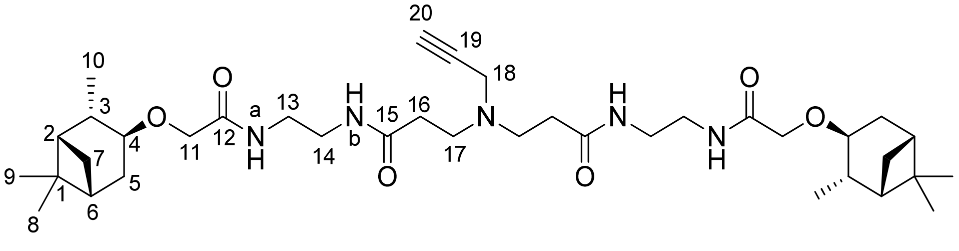

According to **GP2** the diamine **4** (200 mg, 0.71 mmol), compound **1a** (327 mg, 1.55 mmol), EDCI (297 mg, 1.55 mmol), HOBt (237 mg, 1.55 mmol) and NMM (0.17 mL, 157 mg, 1.55 mmol) were dissolved in 10 mL of DMF. After purification via column chromatography ($CHCl_3$/ MeOH/ NH_3OH 9:1:0.1, R_f = 0.50) the desired product was obtained.

Molecular formula: $C_{37}H_{61}N_5O_6$ (yellow oil).

Yield: 171 mg (0.26 mmol, 38%).

^{1}H-NMR (400 MHz, $CDCl_3$, 298 K) δ = 0.86 (s, 6H, 9-H), 1.01 (d, J = 9.7 Hz, 2H, 7-H), 1.10 (d, J = 7.4 Hz, 6H, 10-H), 1.19 (s, 6H, 8-H), 1.71-1.80 (m, 4H, 2,5-H), 1.90-1.93 (m, 2H, 6-H), 1.99-2.07

(m, 2H, 3-H), 2.19 (m, 1H, 20-H), 2.28-2.42 (m, 8H, 5,7-H`,16-H), 2.77 (t, *J* = 6.0 Hz, 4H, 17-H), 3.38-3.45 (m, 10H, 13,14,18-H), 3.62-3.68 (m, 2H, 4-H), 3.88 (d, *J* = 15.1 Hz, 2H, 11-H), 3.97 (d, *J* = 15.1 Hz, 2H, 11-H`), 7.08 (bs, 2H, a-H), 7.50 (bs, 2H, b-H) ppm.

^{13}C-NMR (100 MHz, $CDCl_3$, 298 K) δ = 21.68 (CH_3, 10-C), 24.04 (CH_3, 9-C), 27.67 (CH_3, 8-C), 33.91(CH_2, 16-C), 33.53 (CH_2, 7-C), 35.63 (CH_2, 5-C), 38.62 (C_q, 1-C), 39.93, 39.12 (CH_2, 13,14-C), 41.12 (CH_2, 18-C), 41.47 (CH, 6-C), 44.57 (CH, 3-C), 47.74 (CH, 2-C), 49.61 (CH_2, 17-C), 68.45 (CH_2, 11-C), 74.07 (CH, 20-C), 77.60 (C_q, 19-C), 80.44 (CH, 4-C), 171.72 (C_q, 12-C), 172.95 (C_q, 15-C) ppm.
IR (neat) ν [cm^{-1}]: = 622 (w), 682 (w), 921 (w), 1027 (m), 1117 (m), 1197 (w), 1264 (w), 1315 (w), 1367 (w), 1431 (w), 1455 (w), 1554 (s), 1642 (s), 2868 (s), 2921 (br), 3287 (br).

HRMS-ESI (m/z): Calculated for: $[C_{37}H_{61}N_5O_6Na]^+$: 694.4520, found: 694.4512.

Specific rotation: $[\alpha]^{20}_D$ = + 15.0 ° (c = 0.93, $CHCl_3$).

2.18. (-)-Disopinocampheyl-oxy-acetoxyethylendiamine-alkyne (5b)

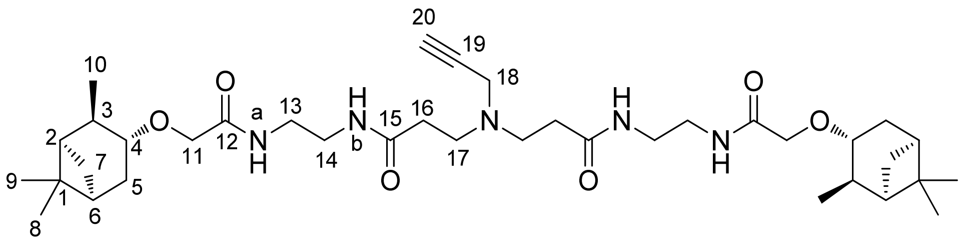

According to **GP2** the diamine **4** (200 mg, 0.71 mmol), compound **1b** (327 mg, 1.55 mmol), EDCI (297 mg, 1.55 mmol), HOBt (237 mg, 1.55 mmol) and NMM (0.17 mL, 157 mg, 1.55 mmol) were dissolved in 10 mL of DMF. After purification via column chromatography ($CHCl_3$/ MeOH/ NH_3OH 9:1:0.1, R_f = 0.50) the desired product was obtained.

Molecular formula: $C_{37}H_{61}N_5O_6$ (yellow oil).

Yield: 221 mg (0.33 mmol, 46 %).

^{1}H-NMR (300 MHz, $CDCl_3$, 298 K): δ = 0.86 (s, 6H, 9-H), 0.99 (d, *J* = 9.7 Hz, 2H, 7-H), 1.09 (d, *J* = 7.4 Hz, 6H, 10-H), 1.19 (s, 6H, 8-H), 1.71-1.79 (m, 4H, 2,5-H), 1.90-1.93 (m, 2H, 6-H), 1.99-2.07 (m, 2H, 3-H), 2.19 (m, 1H, 20-H), 2.28-2.42 (m, 8H, 5,7,16-H), 2.77 (t, *J* = 6.0 Hz, 4H, 17-H), 3.38-3.45 (m, 10H, 13,14,18-H), 3.62-3.68 (m, 2H, 4-H), 3.87 (d, *J* = 15.1 Hz, 2H, 11-H), 3.97 (d, *J* = 15.1 Hz, 2H, 11-H), 7.21 (bs, 2H, a-H), 7.51 (bs, 2H, b-H) ppm.

^{13}C-NMR (75 MHz, $CDCl_3$, 298 K): δ = 21.49 (CH_3, 10-C), 23.85 (CH_3, 9-C), 27.48 (CH_3, 8-C), 33.34 (CH_2, 16-C), 33.71 (CH_2, 7-C), 35.43 (CH_2, 5-C), 38.42 (C_q, 1-C), 38.92, 39.74 (CH_2, 13,14-C), 40.85 (CH_2, 18-C), 41.27 (CH, 6-C), 44.37 (CH, 3-C), 47.54 (CH, 2-C), 49.41 (CH_2, 17-C), 68.25 (CH_2, 11-C), 74.07 (CH, 20-C), 77.40 (C_q, 19-C), 80.24 (CH, 4-C), 171.47 (C_q, 12-C), 172.60 (C_q, 15-C) ppm.

IR (neat) ν [cm^{-1}]: = 620 (w), 709 (w), 785 (w), 921 (m), 970 (w), 1095 (s), 1200 (w), 1264 (w), 1313 (m), 1419 (m), 1453 (w), 1558 (s), 1642 (s), 2872 (m), 2909 (br), 3265 (br).

HRMS-ESI (m/z): Calculated for: $[C_{37}H_{61}N_5O_6Na]^+$: 694.4520, found: 694.4517

Specific rotation: $[\alpha]^{20}_D$ = -14.6 ° (c = 1.11, $CHCl_3$).

2.19. (-)-Dimenthyl-oxy-acetoxyethylenediamine-alkyne (5c)

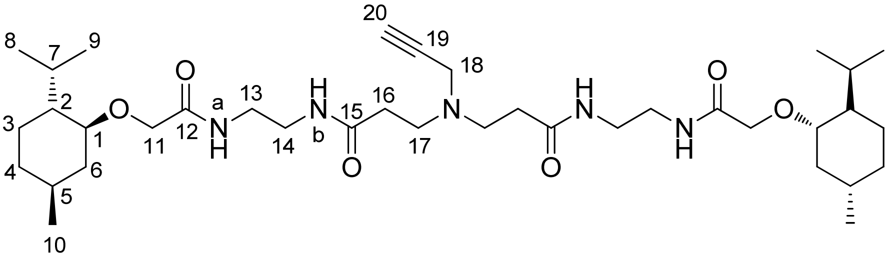

According to **GP2** the diamine **4** (200 mg, 0.71 mmol), compound **1c** (327 mg, 1.55 mmol), EDCI (297 mg, 1.55 mmol), HOBt (237 mg, 1.55 mmol) and NMM (0.17 mL, 157 mg, 1.55 mmol) were dissolved in 10 mL of DMF. After purification via column chromatography ($CHCl_3$/ MeOH/ NH_3OH 9:1:0.1, R_f = 0.51) the desired product was obtained.

Molecular formula: $C_{37}H_{65}N_5O_6$ (yellow oil).

Yield: 156 mg (0.23 mmol, 32%).

^{1}H-NMR (400 MHz, $CDCl_3$, 298 K): δ = 0.75 (d, J = 7.0 Hz, 6H, 9-H), 0.90 (d, J = 7.1 Hz, 6H, 8-H), 0.91 (d, J = 6.5 Hz, 6H, 10-H), 0.80-1.03 (m, 6H, 3,4,6-H), 1.25-1.43 (m, 4H, 2,5-H), 1.60-1.67 (m, 4H, 3,4-H`), 2.02 (m, $J$ = 12.0 Hz, 2H, 6-H`), 2.10 (dsep, J = 7.0 Hz, J = 2.5 Hz, 2H, 7-H), 2.33 (t, J = 6.0 Hz, 4H, 16-H), 2.20 (m, 1H, 20-H), 2.79 (t, J = 6.1 Hz, 4H, 17-H), 3.13 (td, J = 10.6 Hz, 4.1 Hz, 2H, 1-H), 3.38-3.48 (m, 8H, 13,14-H), 3.68 (d, J = 7.0 Hz, 1H, 18-H), 3.73 (d, J = 7.0 Hz, 1H, 18-H), 3.84 (d, J = 15.1 Hz, 2H, 11-H), 4.04 (d, J = 15.1 Hz, 2H, 11-H`), 7.10 (m, 2H, a-H), 7.47 (m, 2H, b-H) ppm.

^{13}C-NMR (100 MHz, $CDCl_3$, 298 K): δ = 16.42 (CH_3, 9-C), 21.30 (CH_3, 8-C), 22.56 (CH_3, 10-C), 23.42 (CH_2, 3-C), 26.16 (CH, 7-C), 31.75 (CH, 5-C), 33.93 (CH_2, 16-C), 34.63 (CH_2, 4-C), 38.95 (CH_2, 13-C), 40.05 (CH_2,14-C), 40.34 (CH_2, 6-C), 41.10 (CH_2, 18-C), 48.25 (CH, 2-C), 49.63 (CH_2, 17-C), 68.04 (CH_2, 11-C), 74.13 (CH, 20-C), 77.56 (C_q, 19-C), 80.65 (CH, 1-C), 172.03 (C_q, 12-C), 172.85 (C_q, 15-C) ppm.

IR (neat) ν [cm^{-1}]: = 545 (m), 624 (s), 653 (s), 803 (w), 918 (w), 1013 (w), 1109 (s), 1181 (w), 1313 (m), 1369 (w), 1415 (w), 1455 (w), 1555 (s), 1642 (s), 2868 (m), 2923 (m), 3269 (br).

HRMS-ESI (m/z): Calculated for $[C_{37}H_{65}N_5O_6H]^+$: 676.5008, found: 676.4985.

Specific rotation: $[\alpha]^{20}_D$ = -18.5° (c = 0.34, $CHCl_3$).

2.20. (+)-Dimenthyl-oxy-acetoxyethylenediamine-alkyne (5d)

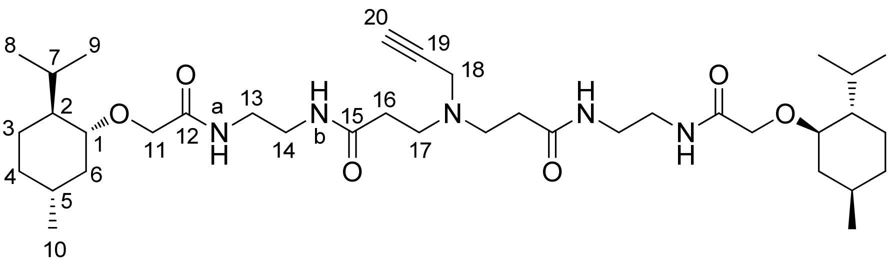

According to **GP2** the diamine **4** (190 mg, 0.67 mmol), compound **1d** (315 mg, 1.47 mmol), EDCI (280 mg, 1.47 mmol), HOBt (226 mg, 1.47 mmol) and NMM (0.15 mL, 148 mg, 1.47 mmol) were

dissolved in 10 mL of DMF. After purification via column chromatography ($CHCl_3$/ MeOH/ NH_3OH 9:1:0.1, R_f = 0.51) the desired product was obtained.

Molecular formula: $C_{37}H_{65}N_5O_6$ (yellow oil).

Yield: 147 mg (0.22 mmol, 33 %).

^{1}H-NMR (400 MHz, $CDCl_3$, 298 K): δ = 0.75 (d, J = 7.0 Hz, 6H, 9-H), 0.90 (d, J = 7.1 Hz, 6H, 8-H), 0.91 (d, J = 6.8 Hz, 6H, 10-H), 0.79-0.88 (m, 6H, 3,4,6-H), 1.25-1.43 (m, 4H, 2,5-H), 1.62 (dt, J = 15.8 Hz, 4.4 Hz, 4H, 3,4-H\`), 2.01 (d, $J$ = 13.4 Hz, 2H, 6-H\`), 2.10 (dsep, J = 7.0 Hz, J = 2.5 Hz, 2H, 7-H), 2.21 (t, J = 2.3 Hz, 1H, 20-H), 2.33 (dd, J = 6.0 Hz, 4H, 16-H), 2.79 (t, J = 6.2 Hz, 4H, 17-H), 3.13 (td, J = 10.6 Hz, 4.1 Hz, 2H, 1-H), 3.27-3.68 (m, 10H, 13,14,18-H), 3.73 (d, J = 7.0 Hz, 1H, 18-H\`), 3.84 (d, $J$ = 15.2 Hz, 2H, 11-H), 4.04 (d, $J$ = 15.2 Hz, 2H, 11-H\`), 7.05-7.18 (m, 2H, a-H), 7.55 (t, J = 4.6 Hz, 2H, b-H) ppm.

^{13}C-NMR (100 MHz, $CDCl_3$, 298 K): δ = 16.24 (CH_3, 9-C), 21.08 (CH_3, 8-C), 22.34 (CH_3, 10-C), 23.26 (CH_2, 3-C), 25.98 (CH, 7-C), 31.61 (CH, 5-C), 33.61 (CH_2, 16-C), 34.43 (CH_2, 4-C), 38.82 (CH_2, 13-C), 39.78 (CH_2,14-C), 40.14 (CH_2, 6-C), 40.90 (CH_2, 18-C), 48.04 (CH, 2-C), 49.40 (CH_2, 17-C), 67.77 (CH_2, 11-C), 74.06 (CH, 20-C), 77.36 (C_q, 19-C), 80.50 (CH, 1-C), 172.03 (C_q, 12-C), 172.88 (C_q, 15-C) ppm.

IR (neat) ν [cm^{-1}]: = 620 (m), 654 (m), 710 (m), 918 (w), 984 (w), 1011 (w), 1100 (s), 1180 (m), 1313 (m), 1415 (m), 1455 (m), 1558 (s), 1642 (s), 2868 (m), 2922 (m), 3277 (br).

HRMS-ESI (m/z): Calculated for $[C_{37}H_{65}N_5O_6H]^+$: 676.5008, found: 676.5004.

Specific rotation: $[\alpha]^{20}_D$ = + 17.2 ° (c = 0.61, $CHCl_3$).

2.21. (-)-Diborneyl-oxy-acetoxyethylenediamine-alkyne (5e)

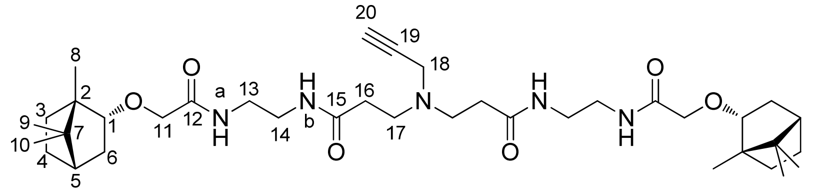

According to **GP2** the diamine **4** (212 mg, 0.75 mmol), compound **1e** (349 mg, 1.55 mmol), EDCI (297 mg, 1.65 mmol), HOBt (252 mg, 1.65 mmol) and NMM (0.2 mL, 166 mg, 1.65 mmol) were dissolved in 10 mL of DMF. After purification via column chromatography ($CHCl_3$/ MeOH/ NH_3OH 9:1:0.1, R_f = 0.51) the desired product was obtained.

Molecular formula: $C_{37}H_{61}N_5O_6$ (yellow oil).

Yield: 162 mg (0.23 mmol, 32%).

^{1}H-NMR (400 MHz, $CDCl_3$, 298 K): δ = 0.81 (s, 6H, 10-H), 0.83 (s, 6H, 9-H), 0.87 (s, 6H, 8-H), 1.00 (dd, J = 13.2 Hz, J = 3.3 Hz, 2H, 6-H), 1.17-1.28 (m, 4H, 3,4-H), 1.61-1.75 (m, 4H, 5-H,4-H\`), 1.86-1.93 (m, 2H, 3-H\`), 2.08-2.16 (m, 2H, 6-H\`), 2.19 (t, $J$ = 2.3 Hz, 1H, 20-H), 2.33 (t, $J$ = 6.1 Hz, 4H, 16-H), 2.78 (t, $J$ = 6.1 Hz, 4H, 17-H), 3.35-3.45 (m, 10H, 13,14,18-H), 3.59-3.63 (m, 2H, 1-H), 3.84 (d, $J$ = 15.2 Hz, 2H, 11-H), 3.93 (d, $J$ = 15.2 Hz, 2H, 11-H\`), 7.08 (t, J = 5.7 Hz, 2H, a-H), 7.50 (t, J = 4.6 Hz, 2H, b-H) ppm.

^{13}C-NMR (100 MHz, $CDCl_3$, 298 K): δ = 14.38 (CH_3, 8-C), 19.08 (CH_3, 10-C), 20.01 (CH_3, 9-C), 26.98 (CH_2, 3-C), 28.46 (CH_2, 4-C), 33.93 (CH_2, 16-C), 36.11 (CH_2, 6-C), 39.14 (CH_2 ,13-C), 39.94 (CH_2, 14-C), 41.15 (CH_2, 18-C), 45.10 (CH, 5-C), 48.32 (C_q, 7-C), 49.57 (C_q, 2-C), 49.61 (CH_2, 17-C), 69.66 (CH_2, 11-C), 74.09 (CH, 20-C), 77.61 (C_q, 19-C), 86.55 (CH, 1-C), 171.82 (C_q, 12-C), 172.92 (C_q, 15-C) ppm.

IR (neat) ν [cm^{-1}]: = 585 (w), 660 (w), 790 (w), 1024 (w), 1052 (w), 1097 (m), 1118 (m), 1258 (w), 1342 (w), 1452 (w), 1630 (m), 1656 (s), 2876 (w), 2949 (br), 3307 (br).

HRMS-ESI (m/z): Calculated for $[C_{37}H_{61}N_5O_6H]^+$: 672.4695, found: 672.4687.

Specific rotation: $[\alpha]^{20}_D$ = - 11.3 ° (c = 0.88, MeOH).

2.22. (+)-Diborneyl-oxy-acetoxyethylenediamine-alkyne (5f)

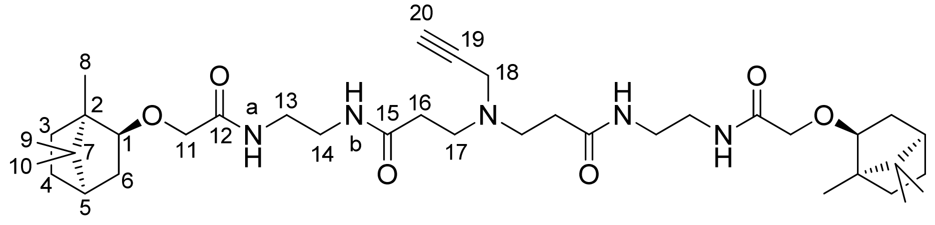

According to **GP2** the diamine **4** (200 mg, 0.71 mmol), compound **1f** (327 mg, 1.56 mmol), EDCI (298 mg, 1.56 mmol), HOBt (245 mg, 1.56 mmol) and NMM (0.17 mL, 157 mg, 1.56 mmol) were dissolved in 10 mL of DMF. After purification via column chromatography ($CHCl_3$/ MeOH/ NH_3OH 9:1:0.1, R_f = 0.51) the desired product was obtained.

Molecular formula: $C_{37}H_{61}N_5O_6$ (yellow oil).

Yield: 188 mg (0.28 mmol, 39 %).

^{1}H-NMR (400 MHz, $CDCl_3$, 298 K): δ = 0.82 (s, 6H, 10-H), 0.84 (s, 6H, 9-H), 0.88 (s, 6H, 8-H), 1.00 (dd, J = 13.2 Hz, J = 3.3 Hz, 2H, 6-H), 1.14-1.30 (m, 4H, 3,4-H), 1.61-1.75 (m, 4H, 5-H,4-H`), 1.85-1.95 (m, 2H, 3-H`), 2.07-2.18 (m, 2H, 6-H`), 2.19 (t, $J$ = 2.3 Hz, 1H, 20-H), 2.34 (t, $J$ = 6.1 Hz, 4H, 16-H), 2.79 (t, $J$ = 6.2 Hz, 4H, 17-H), 3.33-3.47 (m, 10H, 13,14,18-H), 3.58-3.65 (m, 2H, 1-H), 3.85 (d, $J$ = 15.2 Hz, 2H, 11-H), 3.94 (d, $J$ = 15.2 Hz, 2H, 11-H`), 7.07 (t, J = 5.5 Hz, 2H, a-H), 7.48 (t, J = 4.8 Hz, 2H, b-H) ppm.

^{13}C-NMR (100 MHz, $CDCl_3$, 298 K): δ = 14.21 (CH_3, 8-C), 18.91 (CH_3, 10-C), 19.84 (CH_3, 9-C), 26.81 (CH_2, 3-C), 28.29 (CH_2, 4-C), 33.76 (CH_2, 16-C), 36.60 (CH_2, 6-C), 38.96 (CH_2 ,13-C), 39.81 (CH_2, 14-C), 40.98 (CH_2, 18-C), 44.93 (CH, 5-C), 48.14 (C_q, 7-C), 49.40 (C_q, 2-C), 49.45 (CH_2, 17-C), 69.48 (CH_2, 11-C), 73.89 (CH, 20-C), 77.42 (C_q, 19-C), 86.37 (CH, 1-C), 171.63 (C_q, 12-C), 172.70 (C_q, 15-C) ppm.

IR (neat) ν [cm^{-1}]: = 552 (w), 694 (m), 822 (w), 918 (w), 986 (w), 1016 (w), 1124 (s), 1249 (m), 1315 (m), 1419 (w), 1453 (w), 1559 (m), 1643 (m), 1729 (m), 2874 (s), 2948 (m).

HRMS-ESI (m/z): Calculated for $[C_{37}H_{61}N_5O_6H]^+$: 672.4695, found: 672.4693.
Specific rotation**:** $[\alpha]^{20}_D$ = + 11.8 ° (c = 0.61, MeOH).

2.23. (+)-Disopinocampheyl-oxy-acetoxyethylendiamine-peracetylmaltose (6a)

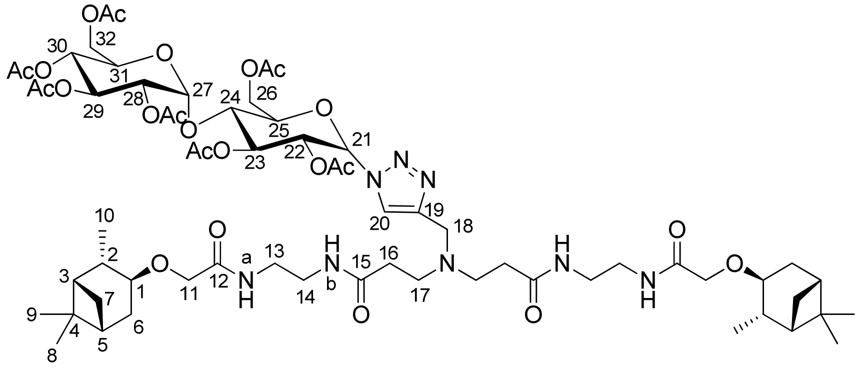

According to **GP3,** compound **5a** (171 mg, 0.26 mmol) was treated with **2** (253 mg, 0.38 mmol), $CuSO_4$ (40 mg, cat.) and sodium ascorbate (29 mg, cat.) in 5 mL of a *t*-BuOH-H_2O mixture (1:1). The resulting solution was stirred over night at room temperature. After workup according to **GP3** and column chromatography ($CHCl_3$ / MeOH 9:1, R_f = 0.23) the desired product was obtained.

Molecular formula: $C_{63}H_{96}N_8O_{23}$ (brown oil).

Yield: 162 mg (0.12 mmol, 46%).

^{1}H-NMR (400 MHz, $CDCl_3$, 298 K): δ = 0.83 (s, 6H, 9-H), 0.97 (d, J = 9.8 Hz, 2H, 7-H), 1.06 (d, J = 7.4 Hz, 6H, 10-H), 1.15 (s, 6H, 8-H), 1.68-1.74 (m, 4H, 2,5H), 1.78 (s, 3H, 22-OAc), 1.88-1.90 (m, 2H, 6-H), 1.95 (s, 3H, 29-OAc), 1.97 (m, 6H, 23,30-OAc), 1.99 (m, 2H, 3-H), 2.01 (s, 3H, 28-OAc), 2.06 (s, 3H, 32-OAc), 2.08 (s, 3H, 26-OAc), 2.24-2.37 (m, 8H, 5,7-H`,16-H), 2.66 (tr, J = 5.9 Hz, 4H, 17-H), 3.32-3.35 (m, 8H, 13,14-H), 3.60-3.64 (m, 2H, 1-H), 3.69 (d, J = 14.8 Hz, 1H, 18-H), 3.74 (d, J = 14.8 Hz, 1H, 18-H), 3.85 (d, J = 15.0 Hz, 2H, 11-H), 3.93 (d, J = 15.0 Hz, 2H, 11-H), 3.94-3.97 (m, 2H, 25,31-H), 4.01 (dd, J = 12.1 Hz, J = 1.9 Hz, 1H, 32-H), 4.12 (t, J = 9.3 Hz, 1H, 24-H), 4.18-4.23 (m, 2H, 26,32-H), 4.47 (dd, J = 12.3 Hz, J = 1.7 Hz, 1H, 26-H), 4.82 (dd, J = 4.0 Hz, J = 10.6 Hz, 1H, 28-H), 5.02 (t, J = 10.0 Hz, 1H, 30-H), 5.27 (t, J = 9.4 Hz, 1H, 22-H), 5.32 (t, J = 10.3 Hz, 1H, 29-H), 5.40 (d, J = 4.1 Hz, 1H, 27-H), 5.42 (t, J = 9.3 Hz, 1H, 23-H), 5.82 (d, J = 9.3 Hz, 1H, 21-H), 7.19 (t, J = 5.5 Hz, 2H, a-H), 7.57 (t, J = 5.0 Hz, 2H, b-H), 7.63 (s, 1H, 20-H) ppm.

^{13}C-NMR (101 MHz, $CDCl_3$, 298 K): δ = 20.34 (CH_3, 22-OAc), 21.02, 20.90, 20.81, 20.79 (6 CH_3, OAc), 21.60 (CH_3, 10-C), 23.96 (CH_3, 9-C), 27.60 (CH_3, 8-C), 33.44 (CH_2, 7-C), 33.99 (CH_2, 16-C), 35.53 (CH_2, 5-C), 38.53 (C_q, 1-C), 39.16 (CH_2, 13-C), 39.56 (CH_2, 14-C), 41.40 (CH, 6-C), 44.46 (CH, 3-C), 47.67 (CH, 2-C), 47.76 (CH_2, 18-C), 49.72 (CH_2, 17-C), 61.64 (CH_2, 32-C), 62.66 (CH_2, 26-C), 68.11 (CH, 30-C), 68.45 (CH_2, 11-C), 68.91 (CH, 31-C), 69.40 (CH, 29-C), 70.22 (CH, 28-C), 71.25 (CH, 22-C), 72.58 (CH, 24-C), 75.10 (CH, 23-C), 75.63 (CH, 25-C), 80.31 (CH, 4-C), 85.45 (CH, 21-C), 96.07 (CH, 27-C), 121.58 (CH, 20-H), 144.32 (C_q, 19-C), 169.45 (C_q, 22-OAc), 169.60 (C_q, 30-OAc), 170.08, 170.04 (2 C_q, 23,29-OAc), 170.53 (C_q, 26-OAc), 170.68 (C_q, 32-OAc), 170.76 (C_q, 28-OAc), 171.47 (C_q, 12-C), 172.87 (C_q, 15-C) ppm.

IR (neat) ν [cm^{-1}]: = 778 (w), 811 (w), 885 (w), 894 (w), 1008 (w), 1022 (m), 1040 (s), 1120 (m), 1113 (m), 1230 (s), 1417 (m), 1435 (w), 1522 (m), 1668 (m), 1757 (s), 2891 (w), 2936 (m), 2957 (m).
HRMS-ESI (m/z): Calculated for $[C_{63}H_{96}N_8O_{23}H]^+$: 1333.6661, found: 1333.6649.

Specific rotation: $[\alpha]^{20}_D$ = + 24.1 ° (c = 0.71, MeOH).

2.24. (-)-Disopinocampheyl-oxy-acetoxyethylendiamine-peracetylmaltose (6b)

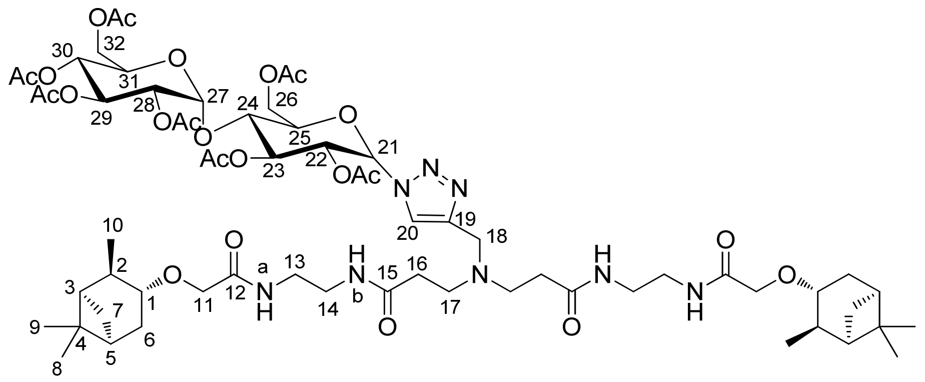

According to **GP3**, compound **5b** (181 mg, 0.28 mmol) was treated with **2** (279 mg, 0.42 mmol), $CuSO_4$ (45 mg, cat.) and sodium ascorbate (33 mg, cat.) in 5 mL of a *t*-BuOH-H_2O mixture (1:1). The resulting solution was stirred over night at room temperature. After workup according to **GP3** and column chromatography ($CHCl_3$/MeOH 9:1, R_f = 0.22) the desired product was obtained.

Molecular formula: $C_{63}H_{96}N_8O_{23}$ (brown oil).

Yield: 144 mg (0.11 mmol, 42%).

^{1}H-NMR (400 MHz, $CDCl_3$, 298 K): δ = 0.86 (s, 6H, 9-H), 1.00 (d, J = 9.8 Hz, 2H, 7-H), 1.09 (d, J = 7.4 Hz, 6H, 10-H), 1.19 (s, 6H, 8-H), 1.71-1.78 (m, 4H, 2,5H), 1.82 (s, 3H, 22-OAc), 1.88-1.93 (m, 2H, 6-H), 1.99 (s, 3H, 29-OAc), 2.00 (m, 6H, 23,30-OAc), 2.01 (m, 2H, 3-H), 2.05 (s, 3H, 28-OAc), 2.08 (s, 3H, 32-OAc), 2.11 (s, 3H, 26-OAc), 2.29-2.43 (m, 10H, 5,7,16-H), 2.69 (t, J = 5.9 Hz, 4H, 17-H), 3.34-3.42 (m, 8H, 13,14-H), 3.62-3.67 (m, 2H, 1-H), 3.76 (d, J = 14.8 Hz, 1H, 18-H), 3.74 (d, J = 14.8 Hz, 1H, 18-H), 3.87 (d, J = 15.0 Hz, 2H, 11-H), 3.95 (d, J = 15.0 Hz, 2H, 11-H), 3.94-3.97 (m, 2H, 25,31-H), 4.04 (dd, J = 12.1 Hz, J = 1.9 Hz, 1H, 32-H), 4.19 (t, J = 9.3 Hz, 1H, 24-H), 4.20-4.27 (m, 2H, 26,32-H), 4.51 (dd, J = 12.3 Hz, J = 1.7 Hz, 1H, 26-H), 4.86 (dd, J = 4.0 Hz, J = 10.6 Hz, 1H, 28-H), 5.06 (t, J = 10.0 Hz, 1H, 30-H), 5.30 (t, J = 9.4 Hz, 1H, 22-H), 5.33 (t, J = 10.3 Hz, 1H, 29-H), 5.42 (d, J = 4.1 Hz, 1H, 27-H), 5.48 (t, J = 9.3 Hz, 1H, 23-H), 5.87 (d, J = 9.3 Hz, 1H, 21-H), 7.20 (t, J = 5.5 Hz, 2H, a-H), 7.60 (t, J = 5.0 Hz, 2H, b-H), 7.67 (s, 1H, 20-H) ppm.

^{13}C-NMR (101 MHz, $CDCl_3$, 298 K): δ = 20.12 (CH_3, 22-OAc), 20.59, 20.68, 20.81, (6 CH_3, OAc), 21.38 (CH_3, 10-C), 23.74 (CH_3, 9-C), 27.38 (CH_3, 8-C), 33.23 (CH_2, 7-C), 33.81 (CH_2, 16-C), 35.33 (CH_2, 5-C), 38.32 (C_q, 1-C), 38.93 (CH_2, 13-C), 39.37 (CH_2, 14-C), 41.19 (CH, 6-C), 44.26 (CH, 3-C), 47.46 (CH, 2-C), 47.77 (CH_2, 18-C), 49.53 (CH_2, 17-C), 61.42 (CH_2, 32-C), 62.43 (CH_2, 26-C), 68.19 (CH, 30-C), 68.71 (CH_2, 11-C), 69.19 (CH, 31-C), 70.01 (CH, 29-C), 70.22 (CH, 28-C), 71.25 (CH, 22-C), 72.35 (CH, 24-C), 75.89 (CH, 23-C), 75.45 (CH, 25-C), 80.13 (CH, 4-C), 85.27 (CH, 21-C), 95.86 (CH, 27-C), 121.58 (CH, 20-H), 144.32 (C_q, 19-C), 169.28 (C_q, 22-OAc), 169.41 (C_q, 30-OAc), 169.85, 169.85 (2 C_q, 23,29-OAc), 170.35 (C_q, 26-OAc), 170.49 (C_q, 32-OAc), 170.58 (C_q, 28-OAc), 171.30 (C_q, 12-C), 172.67 (C_q, 15-C) ppm.

IR (neat) ν [cm^{-1}]: = 777 (w), 810 (w), 885 (w), 891 (w), 1006 (w), 1025 (m), 1044 (s), 1101 (m), 1116 (m), 1229 (s), 1401 (m), 1434 (w), 1522 (m), 1667 (m), 1759 (s), 2889 (w), 2936 (m), 2955 (m).
HRMS-ESI (m/z): Calculated for $[C_{63}H_{96}N_8O_{23}H]^+$: 1333.6661, found: 1333.6678.

Specific rotation: $[\alpha]^{20}_D$ = + 3.9 ° (c = 0.88, MeOH).

2.25. (-)-Dimenthyl-oxy-acetoxyethylendiamine-peracetylmaltose (6c)

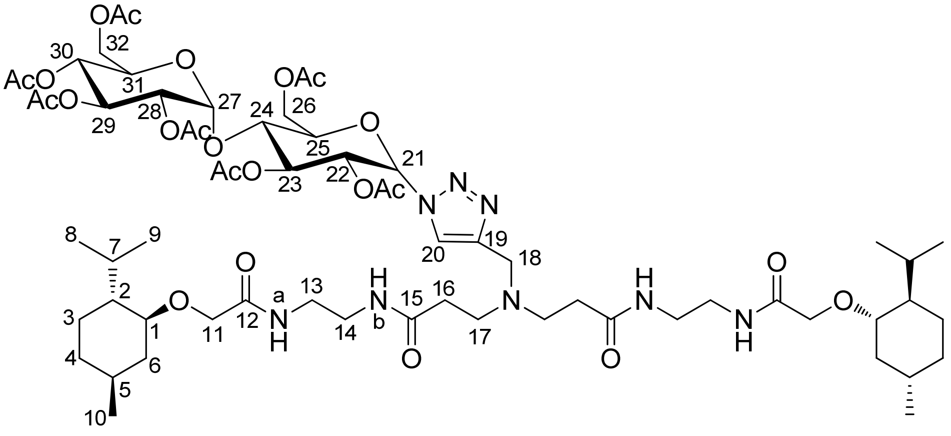

According to **GP3**, compound **5c** (122 mg, 0.18 mmol) was treated with **2** (150 mg, 0.23 mmol), $CuSO_4$ (34 mg, cat.) and sodium ascorbate (25mg, cat.) in 5 mL of a *t*-BuOH-H_2O mixture (1:1). The resulting solution was stirred over night at room temperature. After workup according to **GP3** and column chromatography ($CHCl_3$/ MeOH 9:1, R_f = 0.41) the desired product was obtained.

Molecular formula: $C_{63}H_{100}N_8O_{23}$ (brown viscous oil).

Yield: 143 mg (0.11 mmol, 61%).

^{1}H-NMR (400 MHz, $CDCl_3$, 298 K): δ = 0.75 (d, J = 7.0 Hz, 6H, 9-H), 0.81-0.88 (m, 4H, 4,6-H), 0.89 (d, J = 6.8 Hz, 6H, 8-H), 0.91 (d, J = 6.2 Hz, 6H, 10-H), 0.95 (qd, J = 13.0 Hz, J = 3.4 Hz, 2H, 3-H), 1.22-1.39 (m, 4H, 2,5-H), 1.60-1.66 (m, 4H, 3,4-H), 1.83 (s, 3H, 22-OAc), 2.00 (s, 3H, 29-OAc), 2.02 (m, 6H, 23,30-OAc), 2.06 (s, 3H, 28-OAc), 2.10 (s, 3H, 32-OAc), 2.13 (s, 3H, 26-OAc), 1.99-2.15 (m, 4H, 7-H, 6-H`), 2.32-2.43 (m, 4H, 16-H), 2.64-2.80 (m, 4H, 17-H), 3.13 (dt, J = 10.6 Hz, J = 4.1 Hz, 2H, 1-H), 3.35-3.48 (m, 8H, 13,14-H), 3.74-3.80 (m, 2H, 18-H), 3.84 (d, J = 15.1 Hz, 2H, 11-H), 4.04 (d, J = 15.2 Hz, 2H, 11-H), 3.96-4.00 (m, 2H, 25,31-H), 4.06 (dd, J = 12.4 Hz, J = 2.1 Hz, 1H, 32-H), 4.17 (t, J = 9.3 Hz, 1H, 24-H), 4.23-4.28 (m, 2H, 26,32-H), 4.52 (dd, J = 12.4 Hz, J = 2.1 Hz, 1H, 26-H), 4.87 (dd, J = 10.5 Hz, J = 4.0 Hz, 1H, 28-H), 5.07 (t, J = 9.9 Hz, 1H, 30-H), 5.31 (t, J = 9.4 Hz, 1H, 22-H), 5.37 (dd, J = 9.8 Hz, J = 9.7 Hz, 1H, 29-H), 5.44 (d, J = 3.2 Hz, 1H, 27-H), 5.46 (t, J = 9.3 Hz, 1H, 23-H), 5.86 (d, J = 9.3 Hz, 1H, 21-H), 7.07 (t, J = 5.5 Hz, 2H, a-H), 7.53 (t, J = 4.9 Hz, 2H, b-H), 7.68 (br, 1H, 20-H) ppm.

^{13}C-NMR (101 MHz, $CDCl_3$, 298 K): δ = 16.44 (CH_3, 9-C), 20.46 (CH_3, 22-OAc), 20.91, 20.93, 21.02, 21.14 (6 CH_3, OAc), 21.30 (CH_3, 8-C), 22.56 (CH_3, 10-C), 23.45 (CH_2, 3-C), 26.17 (CH, 7-C), 31.76 (CH, 5-C), 34.07 (CH_2, 16-C), 34.65 (CH_2, 4-C), 39.06 (CH_2 ,13-C), 39.83 (CH_2 ,14-C), 40.35 (CH_2, 6-C), 47.74 (CH_2, 18-C), 48.25 (CH, 2-C), 49.82 (CH_2, 17-C), 61.75 (CH_2, 32-C), 62.76 (CH_2, 26-C), 68.07 (CH_2, 11-C), 68.23 (CH, 30-C), 69.06 (CH, 31-C), 69.53 (CH, 29-C), 70.34 (CH, 28-C), 71.40 (CH, 22-C), 72.67 (CH, 24-C), 75.21 (CH, 23-C), 75.81 (CH, 25-C), 80.66 (CH, 1-C), 85.64 (CH, 21-C), 96.20 (CH, 27-C), 121.67 (CH, 20-H), 144.30 (C_q, 19-C), 169.59 (C_q, 22-OAc), 169.75 (C_q, 30-OAc), 170.22, 170.17 (2 C_q, 23,29-OAc), 170.67 (C_q, 26-OAc), 170.82 (C_q, 32-OAc), 170.91 (C_q, 28-OAc), 172.03 (C_q, 12-C), 172.81 (C_q, 15-C) ppm.

IR (neat) ν [cm^{-1}]: = 792 (w), 867 (w), 875 (w), 1000 (w), 1021 (m), 1038 (s), 1104 (m), 1228 (s), 1377 (m), 1455 (w), 1531 (m), 1650 (m), 1751 (s), 2875 (w), 2942 (m), 2961 (m).

HRMS-ESI (m/z): Calculated for $[C_{63}H_{100}N_8O_{23}H]^+$: 1337.6974, found: 1337.6966.

Specific rotation: $[\alpha]^{20}_D$ = + 10.1 ° (c = 0.31, MeOH).

2.26. (+)-Dimenthyl-oxy-acetoxyethylendiamine-peracetylmaltose (6d)

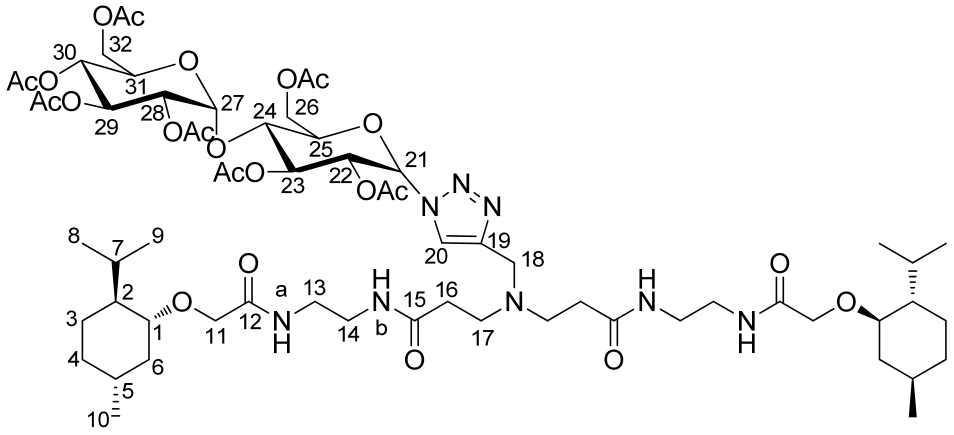

According to **GP3**, compound **5d** (144 mg, 0.22 mmol) was treated with **2** (219 mg, 0.33 mmol), $CuSO_4$ (38 mg, cat.) and sodium ascorbate (29 mg, cat.) in 5 mL of a *t*-BuOH-H_2O mixture (1:1). The resulting solution was stirred over night at room temperature. After workup according to **GP3** and column chromatography ($CHCl_3$/MeOH 9:1, R_f = 0.41) the desired product was obtained.

Molecular formula: $C_{63}H_{100}N_8O_{23}$ (brown viscous oil).

Yield: 143 mg, (0.11 mmol, 50%).

^{1}H-NMR (400 MHz, $CDCl_3$, 298 K): δ = 0.75 (d, J = 7.0 Hz, 6H, 9-H), 0.81-0.84 (m, 4H, 4,6-H), 0.86 (d, J = 6.8 Hz, 6H, 8-H), 0.87 (d, J = 6.2 Hz, 6H, 10-H), 0.87-0-95 (m, 2H, 3-H), 1.20-1.26 (m, 4H, 2,5-H), 1.57-1.62 (m, 4H, 3,4-H`), 1.79 (s, 3H, 22-OAc), 1.97 (s, 3H, 29-OAc), 1.99 (m, 6H, 23,30-OAc), 2.04 (s, 3H, 28-OAc), 2.07 (s, 3H, 32-OAc), 2.09 (s, 3H, 26-OAc), 1.99-2.15 (m, 4H, 7-H, 6-H), 2.38-2.43 (m, 4H, 16-H), 2.71-2.80 (m, 4H, 17-H), 3.10 (dt, J = 10.6 Hz, J = 4.1 Hz, 2H, 1-H), 3.33-3.39 (m, 8H, 13,14-H), 3.74-3.84 (m, 4H, 18,11-H), 3.95-4.01 (m, 2H, 25,31-H), 4.03-4.06 (m, 1H, 32-H), 4.04 (d, J = 15.2 Hz, 2H, 11-H) 4.15 (t, J = 9.3 Hz, 1H, 24-H), 4.20-4.24 (m, 2H, 26,32-H), 4.49 (dd, J = 12.4 Hz, J = 2.1 Hz, 1H, 26-H), 4.85 (dd, J = 10.5 Hz, J = 4.0 Hz, 1H, 28-H), 5.03 (t, J = 9.9 Hz, 1H, 30-H), 5.29 (t, J = 9.4 Hz, 1H, 22-H), 5.31 (t, J = 9.8 Hz, J = 9.7 Hz, 1H, 29-H), 5.40 (d, J = 3.2 Hz, 1H, 27-H), 5.44 (tr, J = 9.3 Hz, 1H, 23-H), 5.84 (d, J = 9.3 Hz, 1H, 21-H), 7.11 (t, J = 5.5 Hz, 2H, a-H), 7.67 (t, J = 4.9 Hz, 2H, b-H), 7.78 (br, 1H, 20-H) ppm.

^{13}C-NMR (101 MHz,$CDCl_3$, 298 K): δ = 16.15 (CH_3, 9-C), 20.16 (CH_3, 22-OAc), 20.61 (CH_3, 8-C), 20.63, 20.72, 20.83, 20.99 (6 CH_3, OAc), 22.26 (CH_3, 10-C), 23.16 (CH_2, 3-C), 25.87 (CH, 7-C), 31.45 (CH, 5-C), 33.33 (CH_2, 16-C), 34.35 (CH_2, 4-C), 38.74 (CH_2 ,13-C), 39.48 (CH_2 ,14-C), 40.03 (CH_2, 6-C), 47.34 (CH_2, 18-C), 47.94 (CH, 2-C), 49.41 (CH_2, 17-C), 61.50 (CH_2, 32-C), 62.90 (CH_2, 26-C), 67.69 (CH_2, 11-C), 67.98 (CH, 30-C), 68.74 (CH, 31-C), 69.26 (CH, 29-C), 70.08 (CH, 28-C), 71.15 (CH, 22-C), 72.45 (CH, 24-C), 74.87 (CH, 23-C), 75.50 (CH, 25-C), 80.41 (CH, 1-C), 85.34 (CH, 21-C), 95.91 (CH, 27-C), 122.20 (CH, 20-H), 143.10 (C_q, 19-C), 169.29 (C_q, 22-OAc), 169.47 (C_q, 30-OAc), 169.91, 170.17 (2 C_q, 23,29-OAc), 170.43 (C_q, 26-OAc), 170.56 (C_q, 32-OAc), 170.61 (C_q, 28-OAc), 171.87 (C_q, 12-C), 172.76 (C_q, 15-C) ppm.

IR (neat) ν [cm^{-1}]: = 791 (w), 866 (w), 879 (w), 999 (w), 1022 (m), 1041 (s), 1104 (m), 1228 (s), 1377 (m), 1455 (w), 1531 (m), 1649 (m), 1712 (w), 1751 (s), 2874 (w), 2942 (m), 2960 (m).

HRMS-ESI (m/z): Calculated for $[C_{63}H_{100}N_8O_{23}H]^+$: 1337.6974, found: 1337.6991.

Specific rotation: $[\alpha]^{20}_D$ = + 22.7 ° (c = 0.61, MeOH).

2.27. (-)-Diborneyl-oxy-acetoxyethylendiamine-peracetylmaltose (6e)

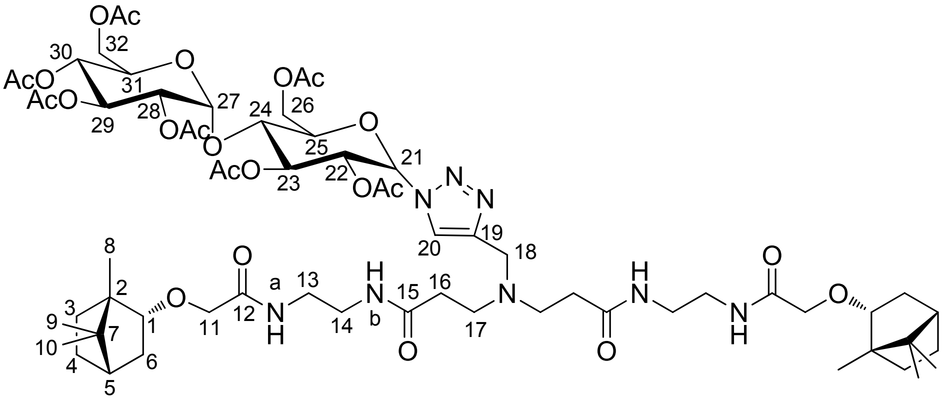

According to **GP3**, compound **5e** (154 mg, 0.23 mmol) was treated with **2** (229 mg, 0.35 mmol), $CuSO_4$ (36 mg, cat.) and sodium ascorbate (29mg, cat.) in 5 mL of a *t*-BuOH-H_2O mixture (1:1). The resulting solution was stirred over night at room temperature. After workup according to **GP3** and column chromatography ($CHCl_3$/MeOH 9:1, R_f = 0.39) the desired product was obtained.

Molecular formula: $C_{63}H_{96}N_8O_{23}$ (light brown viscous oil).

Yield: 108 mg (0.08 mmol, 35%).

^{1}H-NMR (400 MHz, $CDCl_3$, 298 K): δ = 0.82 (s, 6H, 10-H), 0.84 (s, 6H, 9-H), 0.88 (s, 6H, 8-H), 1.01 (dd, J = 13.1 Hz, J = 3.2 Hz, 2H, 6-H), 1.17-1.27 (m, 6H, 3,5-H), 1.64-1.72 (m, 4H, 4-H), 1.83 (s, 3H, 22-OAc), 1.88-1.95 (m, 2H, 3-H), 2.00 (s, 3H, 29-OAc), 2.02 (m, 6H, 23,30-OAc), 2.06 (s, 3H, 28-OAc), 2.08-2.16 (m, 4H, 6-H), 2.09 (s, 3H, 32-OAc), 2.13 (s, 3H, 26-OAc), 2.37 (m, 4H, 16-H), 2.71 (m, 4H, 17-H), 3.36-3.45 (m, 8H, 13,14-H), 3.61-3.64 (m, 2H, 1-H), 3.77 (m, 2H, 18-H), 3.86 (d, J = 15.2 Hz, 2H, 11-H), 3.95 (d, J = 15.2 Hz, 2H, 11-H), 3.94-4.00 (m, 2H, 25,31-H), 4.07 (dd, J = 12.4 Hz, J = 2.2 Hz, 1H, 32-H), 4.16 (tr, J = 9.2 Hz, 1H, 24-H), 4.23-4.28 (m, 2H, 26,32-H), 4.52 (dd, J = 12.5 Hz, J = 2.2 Hz, 1H, 26-H), 4.87 (dd, J = 4.0 Hz, J = 10.6 Hz, 1H, 28-H), 5.07 (t, J = 9.9 Hz, 1H, 30-H), 5.30 (t, J = 9.4 Hz, 1H, 22-H), 5.37 (dd, J = 10.5 Hz, J = 10.4 Hz, 1H, 29-H), 5.44 (d, J = 3.8 Hz, 1H, 27-H), 5.46 (t, J = 9.2 Hz, 1H, 23-H), 5.86 (d, J = 9.3 Hz, 1H, 21-H), 7.06 (t, J = 5.4 Hz, 2H, a-H), 7.54 (t, J = 4.8 Hz, 2H, b-H), 7.67 (s, 1H, 20-H) ppm.

^{13}C-NMR (101 MHz, $CDCl_3$, 298 K): δ = 14.41 (CH_3, 8-C), 19.11 (CH_3, 10-C), 20.04 (CH_3, 9-C), 20.46 (CH_3, 22-OAc), 20.91, 20.92, 21.02, 21.14 (6 CH_3, OAc), 27.02 (CH_2, 3-C), 28.48 (CH_2, 4-C), 34.13 (CH_2, 16-C), 36.13 (CH_2, 6-C), 39.25 (CH_2 ,13-C), 39.74 (CH_2, 14-C), 45.13 (CH, 5-C), 47.86 (CH_2, 18-C), 48.34 (C_q, 7-C), 49.60 (C_q, 2-C), 49.89 (CH_2, 17-C), 61.74 (CH_2, 32-C), 62.75 (CH_2, 26-C), 68.22 (CH, 30-C), 69.05 (CH, 31), 69.52 (CH, 29), 69.70 (CH_2, 11-C), 70.33 (CH, 28), 71.39 (CH, 22), 72.65 (CH, 24), 75.20 (CH, 23), 75.81 (CH, 25), 85.64 (CH, 1-C), 86.53 (CH, 21-C), 96.19 (CH, 27-C), 121.60 (CH, 20-H), 144.48 (C_q, 19-C), 169.60 (C_q, 22-OAc), 169.73 (C_q, 30-OAc), 170.16 (C_q,

23-OAc), 170.21 (C_q, 29-OAc), 170.66 (C_q, 26-OAc), 170.81 (C_q, 32-OAc), 170.89 (C_q, 28-OAc), 171.71 (C_q, 12-C), 172.92 (C_q, 15-C) ppm.

IR (neat) ν [cm^{-1}]: = 785 (w), 886 (w), 894 (w), 989 (w), 1035 (s), 1121 (m), 1224 (s), 1368 (m), 1441 (w), 1553 (m), 1649 (m), 1711 (w), 1747 (s), 2877 (w), 2940 (m), 2952 (m).

HRMS-ESI (m/z): Calculated for $[C_{63}H_{96}N_8O_{23}H]^+$: 1333.6661, found: 1333.6637.

Specific rotation: $[\alpha]^{20}_{D}$ = + 6.3 ° (c = 0.55, MeOH).

2.28. (+)-Diborneyl-oxy-acetoxyethylendiamine-peracetylmaltose (6f)

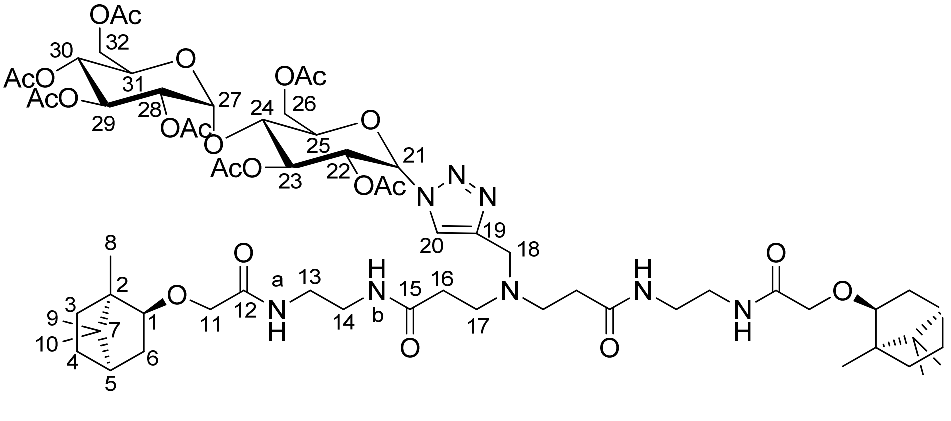

According to **GP3,** compound **5f** (153 mg, 0.24 mmol) was treated with **2** (236 mg, 0.36 mmol), $CuSO_4$ (40 mg, cat.) and sodium ascorbate (30 mg, cat.) in 5 mL of a *t*-BuOH-H_2O mixture (1:1). The resulting solution was stirred over night at room temperature. After workup according to **GP3** and column chromatography ($CHCl_3$/MeOH 9:1, R_f = 0.41) the desired product was obtained.

Molecular formula: $C_{63}H_{96}N_8O_{23}$ (light brown viscous oil).

Yield: 123 mg (0.09 mmol, 38%).

^{1}H-NMR (300 MHz, $CDCl_3$, 298 K): δ = 0.81 (s, 6H, 10-H), 0.82 (s, 6H, 9-H), 0.87 (s, 6H, 8-H), 1.00 (dd, J = 13.1 Hz, 3.2 Hz, 2H, 6-H), 1.23 (dd, J = 9.7 Hz, 7.8 Hz, 6H, 3,5-H), 1.62-1.65 (m, 4H, 4-H), 1.82 (s, 3H, 22-OAc), 1.89-1.90 (m, 2H, 3-H), 1.99 (s, 3H, 29-OAc), 2.01-2.03 (m, 6H, 23,30-OAc), 2.08 (s, 3H, 28-OAc), 2.07-2.13 (m, 4H, 6-H), 2.12 (s, 3H, 32-OAc), 2.13 (s, 3H, 26-OAc), 2.35 (t, J = 6.0 Hz, 4H,16-H), 2.70 (t, J = 5.9 Hz, 4H, 17-H), 3.27-3.49 (m, 8H, 13,14-H), 3.57-3.67 (m, 2H, 1-H), 3.76 (d, J = 3.7 Hz, 2H, 18-H), 3.84 (d, J = 15.2 Hz, 2H, 11-H), 3.97 (d, J = 15.2 Hz, 2H,11-H), 4.01-4.09 (m, 3H, 25,31,32-H), 4.15 (dd, J = 17.5 Hz, 8.4 Hz, 1H, 24-H), 4.24 (dd, J = 12.6 Hz, 3.3 Hz, 2H, 26,32-H), 4.50 (dd, J = 12.3 Hz, 2.1 Hz, 1H, 26-H), 4.86 (dd, J = 4.0 Hz, 10.5 Hz, 1H, 28-H), 5.05 (t, J = 9.8 Hz, 1H, 30-H), 5.24-5.37 (m, 2H, 22,29-H), 5.44-5.46 (m, 2H, 23,27-H), 5.85 (d, J = 9.2 Hz, 1H, 21-H), 7.06 (t, J = 4.9 Hz, 2H, a-H), 7.54 (t, J = 4.9 Hz, 2H, b-H), 7.66 (s, 1H, 20-H) ppm.

^{13}C-NMR (75 MHz, $CDCl_3$, 298 K): δ = 14.19 (CH_3, 8-C), 18.89 (CH_3, 10-C), 19.82 (CH_3, 9-C), 20.24 (CH_3, 22-OAc), 20.70, 20.80, 20.92, (6 CH_3, OAc), 26.79 (CH_2, 3-C), 28.26 (CH_2, 4-C), 33.92 (CH_2, 16-C), 35.91 (CH_2, 6-C), 39.04 (CH_2 ,13-C), 39.50 (CH_2, 14-C), 44.90 (CH, 5-C), 47.63 (CH_2, 18-C), 48.11 (C_q, 7-C), 49.37 (C_q, 2-C), 49.63 (CH_2, 17-C), 61.52 (CH_2, 32-C), 62.57 (CH_2, 26-C), 68.00 (CH, 30-C), 68.82 (CH, 31), 69.30 (CH, 29), 69.47 (CH_2, 11-C), 70.11 (CH, 28), 71.15 (CH, 22), 72.45 (CH, 24), 75.00 (CH, 23), 75.56 (CH, 25), 85.39 (CH, 1-C), 86.30 (CH, 21-C), 95.96 (CH, 27-

C), 121.36 (CH, 20-H), 144.22 (C_q, 19-C), 169.36 (C_q, 22-OAc), 169.50 (C_q, 30-OAc), 169.93 (C_q, 23-OAc), 169.98 (C_q, 29-OAc), 170.42 (C_q, 26-OAc), 170.57 (C_q, 32-OAc), 170.66 (C_q, 28-OAc), 171.46 (C_q, 12-C), 172.70 (C_q, 15-C) ppm.

IR (neat) ν [cm^{-1}]: = 784 (w), 885 (w), 991 (w), 1035 (s), 1121 (m), 1224 (s), 1368 (m), 1441 (w), 1554 (m), 1651 (m), 1710 (w), 1749 (s), 2877 (w), 2937 (m), 2952 (m).

HRMS-ESI (m/z): Calculated for $[C_{63}H_{96}N_8O_{23}H]^+$: 1333.6661, found: 1333.6654.

Specific rotation: $[\alpha]^{20}_D$ = + 17.3 ° (c = 0.40, MeOH).

2.29. (+)-Diisopinocampheyl-oxy-acetoxyethylenediamine-maltose (7a)

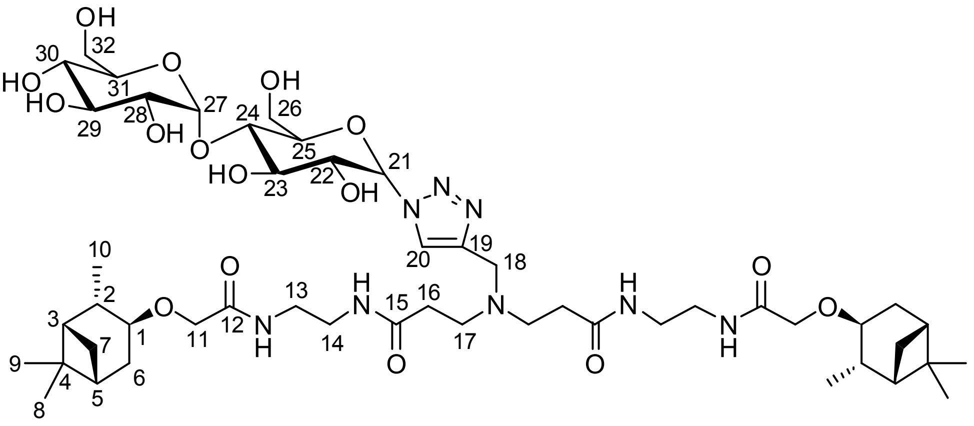

According to **GP4** compound **6a** (162 mg, 0.12 mmol) was dissolved in methanol and a catalytical amount of NaOMe was added (10 mg). After 2 h the resulting mixture was neutralized, filtered and evaporated according to **GP4**.

Yield: 69 mg (0.07 mmol, 58%).

Molecular formula: $C_{49}H_{82}N_8O_{16}$ (brownish solid).

^{1}H-NMR (400 MHz, Methanol-d$_4$, 298 K): δ = 0.92 (s, 6H, 9-H), 1.08 (d, J = 9.6 Hz, 2H, 7-H), 1.14 (d, J = 7.4 Hz, 6H, 10-H), 1.23 (s, 6H, 8-H), 1.78-1.84 (m, 4H, 2,5-H), 1.92-1.94 (m, 2H, 6-H), 2.07-2.14 (m, 2H, 3-H), 2.33-2.38 (m, 2H, 7-H\`), 2.42-2.55 (m, 5H, 5-H\`,16-H), 2.91 (br., 4H, 17-H), 3.26-3.40 (m, 10H, 13,14,30-H), 3.46-3.50 (m, 2H, 28-H), 3.61-4.02 (m, 13H, 4,18,22,23,24,25,26,29,31,32-H) 3.92 (d, J = 14.9 Hz, 2H, 11-H), 4.00 (d, J = 14.9 Hz, 2H, 11-H\`), 5.25 (m, 1H, 27-H), 5.67 (br., 1H, 21-H), 8.23 (br., 1H, 20-H) ppm.

^{13}C-NMR (101 MHz, Methanol-d$_4$, 298 K): δ = 22.71 (CH_3, 10-C), 25.07 (CH_3, 9-C), 28.80 (CH_3, 8-C), 34.37 (CH_2, 16-C), 34.96 (CH_2, 7-C), 37.14 (CH_2, 5-C), 40.32 (C_q, 1-C), 40.57 (CH_2, 13-C), 40.90 (CH_2, br., 14-C), 43.49 (CH, 6-C), 46.34 (CH, 3-C), 49.73 (CH, 2-C), 51.43 (CH_2, 17-C), 62.65 (CH_2, 32-C), 63.57 (CH_2, 26-C), 70.00 (CH_2, 11-C), 72.33 (CH, 30-C), 74.60 (CH, 22-C), 74.99 (CH, 28-C), 75.72, 75.90 (2 CH, 29,31), 78.68 (CH, 4-C), 78.98 (br. CH, 23-C), 80.50 (br. CH, 25-C), 81.06 (CH, 24-C), 82.12 (CH, 4-C), 90.32 (br. CH, 21-C), 103.75 (27-C), 126.10 (CH, 20-C), 144.47 (C_q, 19-C), 174.21 (C_q, 12-C), 175.47 (C_q, 15-C) ppm.

HRMS-ESI (m/z): Calculated for $[C_{49}H_{82}N_8O_{16}HNa]^{2+}$: 531.2913, found: 531.2921.

IR (neat) ν [cm^{-1}]: = 777 (w), 833 (w), 860 (w), 900 (w), 919 (w), 1035 (s), 1098 (s), 1195 (w), 1236 (w), 1330 (w), 1351 (w), 1367 (w), 1449 (m), 1539 (m), 1649 (s), 2922 (m), 3309 (br).

Specific rotation: $[\alpha]^{20}_{D}$ = + 22.3° (c = 0.38, MeOH).

Melting point: 105 °C (MeOH).

2.30. (-)-Diisopinocampheyl-oxy-acetoxyethylenediamine-maltose (7b)

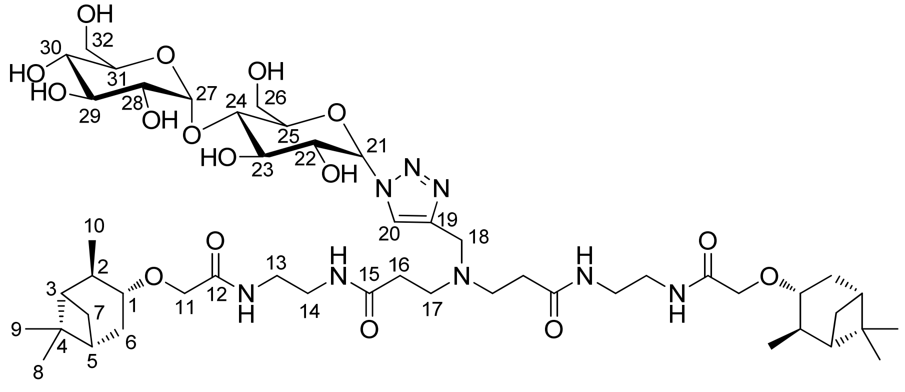

According to **GP4** compound **6b** (155 mg, 0.12 mmol) was dissolved in methanol and a catalytical amount of NaOMe was added (10 mg). After 2 h the resulting mixture was neutralized, filtered and evaporated according to **GP4**.

Yield: 71 mg (0.07 mmol, 59 %).

Molecular formula: $C_{49}H_{82}N_8O_{16}$ (brownish solid).

^{1}H-NMR (400 MHz, Methanol-d$_4$, 298 K): δ = 0.92 (s, 6H, 9-H), 1.08 (d, J = 9.6 Hz, 2H, 7-H), 1.14 (d, J = 7.4 Hz, 6H, 10-H), 1.23 (s, 6H, 8-H), 1.78-1.84 (m, 4H, 2,5-H), 1.92-1.94 (m, 2H, 6-H), 2.07-2.14 (m, 2H, 3-H), 2.33-2.38 (m, 2H, 7-H`), 2.42-2.55 (m, 5H, 5-H`,16-H), 2.91 (br., 4H, 17-H), 3.26-3.40 (m, 10H, 13,14,30-H), 3.46-3.50 (m, 2H, 28-H), 3.61-4.02 (m, 13H, 4,18,22,23,24,25,26,29,31,32-H) 3.92 (d, J = 14.9 Hz, 2H, 11-H), 4.00 (d, J = 14.9 Hz, 2H, 11-H`), 5.25 (m, 1H, 27-H), 5.67 (bs, 1H, 21-H), 8.23 (bs, 1H, 20-H) ppm.

^{13}C-NMR (101 MHz, Methanol-d$_4$, 298 K): δ = 22.73 (CH_3, 10-C), 25.05 (CH_3, 9-C), 28.78 (CH_3, 8-C), 34.35 (CH_2, 16-C), 34.91 (CH_2, 7-C), 37.08 (CH_2, 5-C), 40.41 (C_q, 1-C), 40.61 (CH_2, 13-C), 40.92 (CH_2, br., 14-C), 43.50 (CH, 6-C), 46.37 (CH, 3-C), 49.71 (CH, 2-C), 51.39 (CH_2, 17-C), 62.68 (CH_2, 32-C), 63.71 (CH_2, 26-C), 70.02 (CH_2, 11-C), 72.35 (CH, 30-C), 74.61 (CH, 22-C), 74.92 (CH, 28-C), 75.68, 75.91 (2 CH, 29,31), 78.71 (CH, 4-C), 79.00 (br. CH, 23-C), 80.51 (br. CH, 25-C), 81.03 (CH, 24-C), 82.18 (CH, 4-C), 90.40 (br. CH, 21-C), 103.80 (27-C), 126.12 (CH, 20-C), 144.40 (C_q, 19-C), 174.27 (C_q, 12-C), 175.50 (C_q, 15-C) ppm.

IR (neat) ν [cm^{-1}]: = 668 (w), 776 (w), 833 (w), 919 (w), 1034 (s), 1973 (s), 1097 (s), 1237 (w), 1328 (w), 1367 (w), 1450 (w), 1538 (m), 1650 (s), 2921 (m), 3306 (br).

HRMS-ESI (m/z): Calculated for $[C_{49}H_{82}N_8O_{16}Na]^+$:1061.5741, found: 1061.5752.

Specific rotation: $[\alpha]^{20}_{D}$ = - 8.0° (c = 0.63, MeOH).

Melting point: 104 °C (MeOH).

2.31. (-)-Dimenthyl-oxy-acetoxyethylenediamine-maltose (7c)

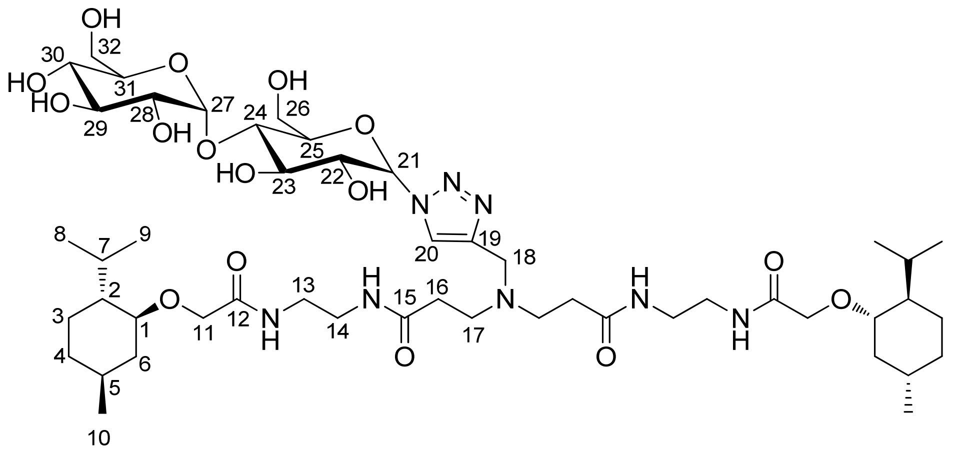

According to **GP4** compound **6c** (143 mg, 0.12 mmol) was dissolved in methanol and a catalytical amount of NaOMe was added (10 mg). After 2 h the resulting mixture was neutralized, filtered and evaporated according to **GP4**.

Yield: 69 mg (0.07 mmol, 58 %).

Molecular formula: $C_{49}H_{86}N_8O_{16}$ (brownish solid).

^{1}H-NMR (400 MHz, Methanol-d$_4$, 298 K): δ = 0.79 (d, J = 7.0 Hz, 6H, 9-H), 0.84-0.92 (m, 4H, 4,6-H), 0.91 (d, J = 7.0 Hz, 6H, 8-H), 0.93 (d, J = 6.5 Hz, 6H, 10-H), 1.02 (dq, J = 14.1 Hz, J = 3.3 Hz, 2H, 3-H), 1.24-1.42 (m, 4H, 2,5-H), 1.63-1.69 (m, 4H, 3,4-H`), 2.08 (dm, 2H, $J$ = 11.6 Hz, 6-H`), 2.21 (dsep, J = 7.0 Hz, J = 2.5 Hz, 2H, 7-H), 2.51 (bs, 2H, 16-H), 2.94 (bs, 2H, 17-H), 3.21 (td, J = 10.6 Hz, J = 4.1 Hz, 2H, 1-H), 3.28-3.32 (m, 5H, 14,30-H), 3.36 (m, 4H, 13-H), 3.47 (dd, J = 9.7 Hz, J = 3.7 Hz, 1H, 28-H), 3.61-3.73 (m, 4H, 25,29,31,32-H), 3.77 (t, J = 9.0 Hz, 1H, 24-H), 3.83-3.88 (m, 3H, 23,26-H,32-H`), 3.88 (d, $J$ = 15.1 Hz, 2H, 11-H), 3.96 (t, $J$ = 8.9 Hz, 1H, 22-H), 4.05 (d, $J$ = 15.2 Hz, 2H, 11-H`), 4.02-4.06 (m, 2H, 18-H), 5.25 (d, J = 3.7 Hz, 1H, 27-H), 5.66 (d, J = 8.9 Hz, 1H, 21-H), 8.23 (s, 1H, 20-H) ppm.

^{13}C-NMR (101 MHz, Methanol-d$_4$, 298 K): δ = 17.41 (CH_3, 9-C), 17.41 (CH_3, 9-C), 22.32 (CH_3, 8-C), 23.58 (CH_3, 10-C), 27.67 (CH, 7-C), 25.11 (CH_2, 3-C), 33.57 (CH, 5-C), 34.22 (CH_2, 16-C), 36.47 (CH_2, 4-C), 40.41 (CH_2, 13-C), 40.94 (CH_2,14-C), 42.02 (CH_2, 6-C), 49.16 (CH_2, 18-C), 50.11 (CH, 2-C), 51.47 (CH_2, 17-C), 62.66 (CH_2, 26-C), 63.60 (CH_2, 32-C), 69.60 (CH_2, 11-C), 72.35 (CH, 30-C), 74.60 (CH, 22-C), 75.02 (CH, 28-C), 75.74, 75.92 (2 CH, 29,31), 78.97 (CH, 23-C), 80.49 (CH, 25-C), 81.12 (CH, 24-C), 82.45 (CH, 1-H), 90.32 (CH, 21-C), 103.79 (CH, 27-C), 126.05 (CH, 20-H), 143.98 (C_q, 19-C), 174.52 (C_q, 12-C), 175.25 (C_q, 15-C) ppm.

HRMS-ESI (m/z): Calculated for $[C_{49}H_{86}N_8O_{16}HNa]^{2+}$: 533.3069, found: 533.3078.

IR (neat) ν [cm^{-1}]: = 777 (w), 844 (w), 918 (w), 1039 (s), 1074 (s), 1100 (s), 1237 (w), 1342 (w), 1368 (w), 1450 (m), 1538 (m), 1651 (s), 2870 (w), 2922 (m), 3301 (br).

Specific rotation: $[\alpha]^{20}_D$ = + 6.9 ° (c = 0.38, MeOH)

Melting point: 102 °C (MeOH).

2.32. (+)-Dimenthyl-oxy-acetoxyethylenediamine-maltose (7d)

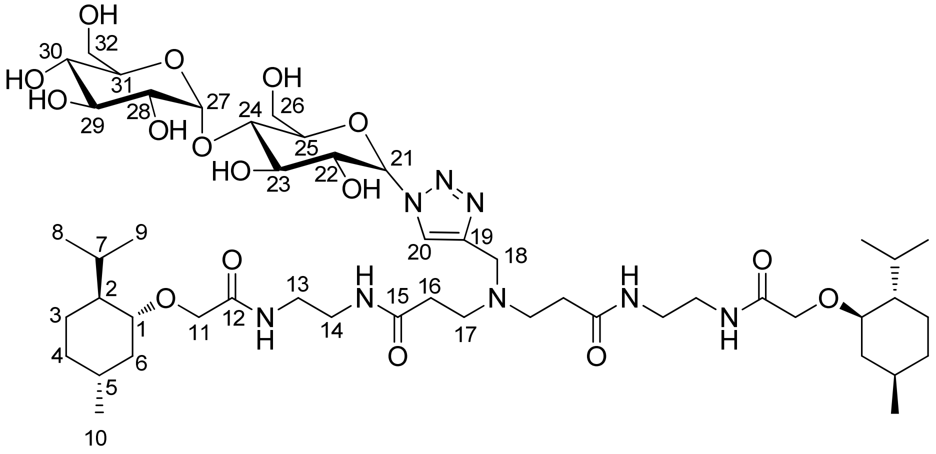

According to **GP4** compound **6d** (158 mg, 0.12 mmol) was dissolved in methanol and a catalytical amount of NaOMe was added (10 mg). After 2 h the resulting mixture was neutralized, filtered and evaporated according to **GP4**.

Yield: 88 mg (0.08 mmol, 69 %).

Molecular formula: $C_{49}H_{86}N_8O_{16}$ (brownish solid).

^{1}H-NMR (300 MHz, Methanol-d$_4$, 298 K): δ = 0.76 (d, J = 7.0 Hz, 6H, 9-H), 0.85-0.87 (m, 4H, 4,6-H), 0.87-1.01 (m, 12H, 8,10-H), 1.02 (dq, J = 14.1 Hz, J = 3.3 Hz, 2H, 3-H), 1.25-1.43 (m, 4H, 2,5-H), 1.60-1.64 (m, 4H, 3,4-H), 2.02-2.06 (m, 2H, 6-H), 2.18 (m, 2H, , 7-H), 2.53 (bs, 2H, 16-H), 3.00 (bs, 2H, 17-H), 3.10-3.35 (m, 11H, 1,13,14,30-H), 3.46 (dd, J = 9.7 Hz, J = 3.7 Hz, 1H, 28-H), 3.55-3.71 (m, 4H, 25,29,31,32-H), 3.76-3.90 (m, 6H 11,23,26,32-H), 3.99 (m, 1H, 22-H), 4.05 (d, J = 15.2 Hz, 2H, 11-H), 4.02-4.06 (m, 2H, 18-H), 5.20 (d, J = 3.7 Hz, 1H, 27-H), 5.64 (d, J = 8.9 Hz, 1H, 21-H), 8.27 (s, 1H, 20-H) ppm.

^{13}C-NMR (75.5 MHz, Methanol-d$_4$, 298 K): δ = 16.65 (CH_3, 9-C), 21.57 (CH_3, 8-C), 22.83 (CH_3, 10-C), 24.35 (CH_2, 3-C), 26.91 (CH, 7-C), 32.83 (CH, 5-C), 34.22 (CH_2, 16-C), 35.73 (CH_2, 4-C), 39.61 (CH_2, 13-C), 40.27 (CH_2,14-C), 41.27 (CH_2, 6-C), 48.93 (CH_2, 18-C), 50.77 (CH, 2-C), 54.00 (CH_2, 17-C), 62.85 (CH_2, 26-C), 65.05 (CH_2, 32-C), 68.78 (CH_2, 11-C), 71.58 (CH, 30-C), 73.85 (CH, 22-C), 74.29 (CH, 28-C), 75.01, 75.26 (2 CH, 29,31), 78.18 (CH, 23-C), 80.38 (CH, 25-C), 81.70 (CH, 24-C), 82.45 (CH, 1-H), 89.61 (CH, 21-C), 103.03 (CH, 27-C), 126.02 (CH, 20-H), 141.12 (C_q, 19-C), 173.82 (C_q, 12-C), 174.11 (C_q, 15-C) ppm.

HRMS-ESI (m/z): Calculated for $[C_{49}H_{86}N_8O_{16}HNa]^{2+}$: 533.3071, found: 533.3078.

IR (neat) ν [cm-1]: = 665 (w), 765 (w), 811 (w), 845 (w), 915 (w), 1054 (s), 1106 (s), 1239 (w), 1336 (w), 1368 (w), 1448 (m), 1538 (m), 1650 (s), 1752 (w), 2922 (m), 2953 (m), 3324 (br).

Melting point: 106 °C (MeOH).

Specific rotation: $[\alpha]^{20}_D$ = + 13.6 (c = 0.13, MeOH).

2.33. (-)-Diborneyl-oxy-acetoxyethylenediamine-maltose (7e)

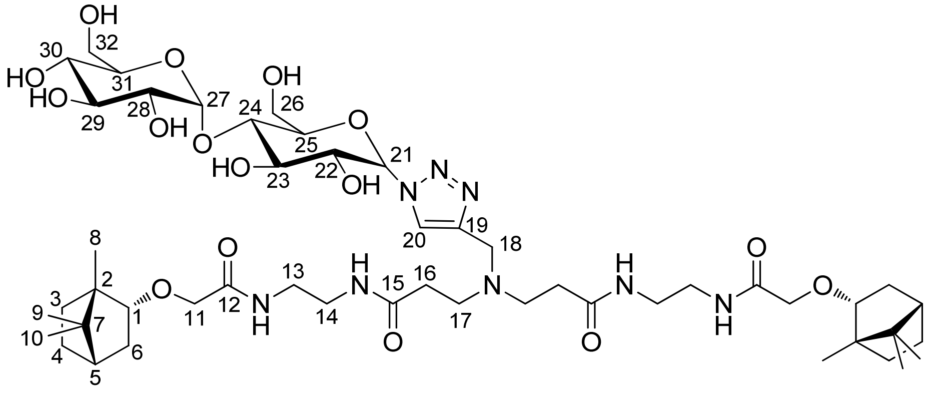

According to **GP4** compound **6e** (108 mg, 0.08 mmol) was dissolved in methanol and a catalytical amount of NaOMe was added (10 mg). After 2 h the resulting mixture was neutralized, filtered and evaporated according to **GP4**.

Yield: 44 mg (0.03 mmol, 38 %).

Molecular formula: $C_{49}H_{82}N_8O_{16}$ (brownish solid).

^{1}H-NMR (400 MHz, Methanol-d$_4$, 298 K): δ = 0.87 (s, 12H, 9,10-H), 0.93 (s, 6H, 8-H), 1.06-1.10 (dm, 2H, 6-H) 1.20-1.31 (m, 4H, 3,4-H), 1.64 (t, 2H, 5-H), 1.71-1.75 (m, 2H, 4-H`), 2.02-2.08 (m, 2H, 3-H`), 2.16-2.21 (m, 2H, 6-H), 2.55 (bs, 4H, 16-H), 3.02 (bs, 4H, 17-H), 3.26-3.38 (m, 10H-9, 13,14,30-H), 3.48 (dd, J = 9.9 Hz, J = 3.4 Hz, 1H, 28-H), 3.61-3.76 (m, 9H, 1,18,24,25,29,31,26-H), 3.83-3.99 (m, 9H, 11,22,23,32-H, 26-H), 5.25 (bs, 1H, 27-H), 5.67 (bs, 1H, 21-H), 8.30 (s, 1H, 20-H) ppm.

^{13}C-NMR (101 MHz, Methanol-d$_4$, 298 K): δ = 14,41 (CH_3, 8-C), 19.17, 20.12 (2 CH_3, 9, 10-C), 27.61 (CH_2, 3-C), 29.06 (CH_2, 4-C), 36.71 (CH_2, 6-C), 40.51 (CH_2, 13-C), 40.84 (C_q, 7-C), 41.03 (br., CH_2, 14-C), 46.19 (CH, 5-C), 50.42 (CH_2, 17-C), 62.65 (CH_2, 32-C), 63.59 (CH_2, 26-C), 70.34 (CH_2, 11-C), 71.43 (CH, 30-C), 74.09 (CH, 22-C), 74.82 (CH, 28-C), 74.99, 75.01 (2 CH, 29,31-C), 79.11 (br., CH, 23-C), 80.18 (br., CH, 25), 81.09 (CH, 24-C), 87.43 (CH, 1-C), 90.41 (CH, 21-C), 102.68 (CH, 27-C), 111.31 (CH, 20-C), 138.52 (C_q, 19-C), 173.30 (C_q, 12-C), 173.37 (C_q, 15-C) ppm.

HRMS-ESI (m/z): Calculated for $[C_{49}H_{82}N_8O_{16}HNa]^{2+}$: 531.2913, found: 531.2918.

IR (neat) ν [cm^{-1}]: = 690 (w), 777 (w), 833 (w), 918 (w), 1037 (s), 1098 (s), 1202 (w), 1236 (w), 1269 (w), 1348 (w), 1368 (w), 1450 (m), 1539 (m), 1650 (s), 2924 (m), 3309 (s).

Specific rotation: $[\alpha]^{20}_D$ = + 15.5 ° (c = 0.23, MeOH).

Melting point: 108 °C (MeOH)

2.34. (+)-Diborneyl-oxy-acetoxyethylenediamine-maltose (7f)

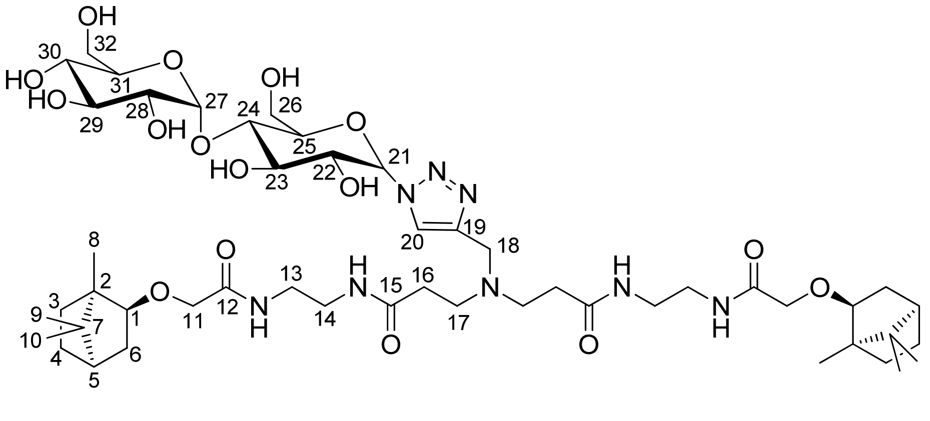

According to **GP4** compound **6f** (155 mg, 0.12 mmol) was dissolved in methanol and a catalytical amount of NaOMe was added (10 mg). After 2 h the resulting mixture was neutralized, filtered and evaporated according to **GP4**.

Yield: 71 mg (0.07 mmol, 55 %).

Molecular formula: $C_{49}H_{82}N_8O_{16}$ (brownish solid).

^{1}H-NMR (300 MHz, Methanol-d$_4$, 298 K): δ = 0.89 (s, 12H, 9,10-H), 0.95 (s, 6H, 8-H), 1.09-1.12 (m, 2H, 6-H) 1.25-1.31 (m, 4H, 3,4-H), 1.61-1.66 (m, 2H, 5-H), 1.71 -1.79 (m, 2H, 4-H), 2.03-2.11 (m, 2H, 3-H), 2.17-2.24 (m, 2H, 6-H), 2.72 (bs, 4H, 16-H), 3.40 (bs, 4H, 17-H), 3.26-3.36 (m, 10H, 13,14,30-H), 3.40 (bs., 4H, 17-H), 3.48 (dd, J = 9.8 Hz, J = 3.5 Hz, 1H, 28-H), 3.63-3.76 (m, 9H, 1,18,24,25,29,31,26-H), 3.85-4.01 (m, 9H, 11,22,23,32-H, 26-H), 5.26 (d, J = 2.8 Hz, 1H, 27-H), 5.72 (bs, 1H, 21-H), 8.46 (s, 1H, 20-H) ppm.

^{13}C-NMR (75.5 MHz, Methanol-d$_4$, 298 K): δ = 14.59 (CH_3, 8-C), 19.34, 20.28 (2 CH_3, 9, 10-C), 27.78 (CH_2, 3-C), 29.23 (CH_2, 4-C), 36.71 (CH_2, 6-C), 40.51 (CH_2, 13-C), 40.84 (C_q, 7-C), 42.63 (br., CH_2, 14-C), 46.35 (CH, 5-C), 50.26 (CH_2, 17-C), 62.65 (CH_2, 26-C), 63.59 (CH_2, 32-C), 70.53 (CH_2, 11-C), 71.20 (CH, 30-C), 73.89 (CH, 22-C), 74.24 (CH, 28-C), 74.98, 75.15 (2 CH, 29,31-C), 79.11 (CH, 23-C), 80.18 (CH, 25-C), 81.09 (CH, 24-C), 87.43 (CH, 1-C), 90.41 (CH, 21-C), 102.68 (CH, 27-C), 111.31 (CH, 20-C), 138.52 (C_q, 19-C), 173.30 (C_q, 12-C), 173.37 (C_q, 15-C) ppm.

HRMS-ESI (m/z): Calculated for $[C_{49}H_{82}N_8O_{16}H]^+$: 1061.5741, found: 1061.5722.

IR (neat) ν [cm^{-1}]: = 661 (w), 773 (w), 838 (w), 916 (w), 1034 (s), 1099 (s), 1120 (s), 1232 (s), 1365 (m), 1452 (w), 1539 (m), 1650 (s), 1747 (w), 2875 (m), 2931 (m), 3325 (br).

Specific rotation: $[\alpha]^{20}_D$ = + 32.2°(c = 0.45, MeOH).

Melting point: 109 °C (MeOH).

3. ITC Measurements

<table>
<tr><th>3.1</th><th>Table 1, Entry A</th></tr>
<tr><td>[7a]: 5 mM, [β-CD] : 1 mM</td><td>T = 23 °C, Rpm = 300, 20 × 10 μL injections</td></tr>
<tr><td>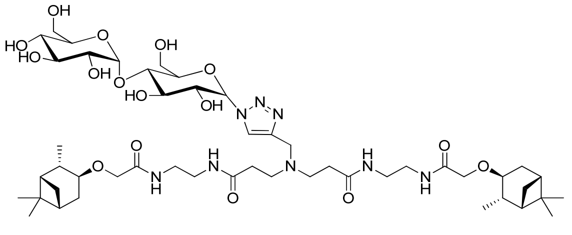</td><td>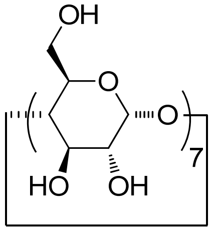</td></tr>
<tr><td>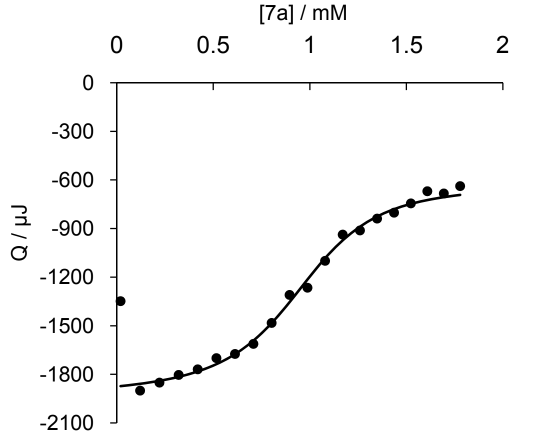
</td><td>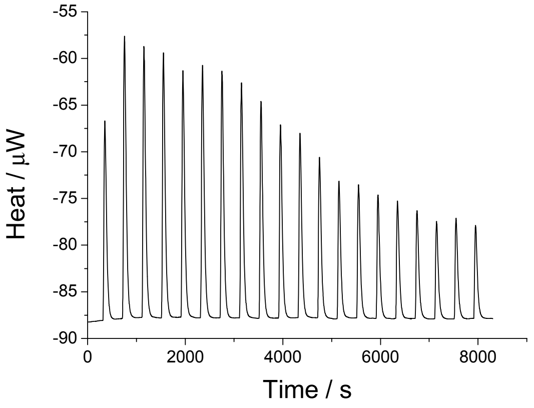
</td></tr>
<tr><td colspan="2">Figure 3.1. Integrated injection peaks of the ITC experiment of 7a with β-CD (left) and raw heat effects (right).</td></tr>
<tr><th>3.2</th><th>Table 1, Entry B</th></tr>
<tr><td>[7a]: 5 mM, [14] : 0.5 mM</td><td>T = 23 °C, Rpm = 300, 20 x 10 μL injections</td></tr>
<tr><td>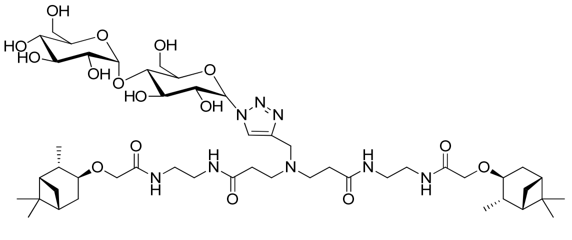</td><td>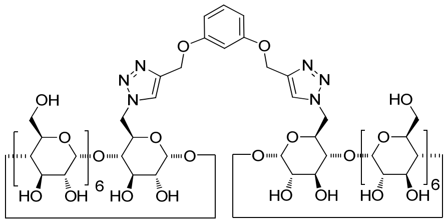</td></tr>
<tr><td>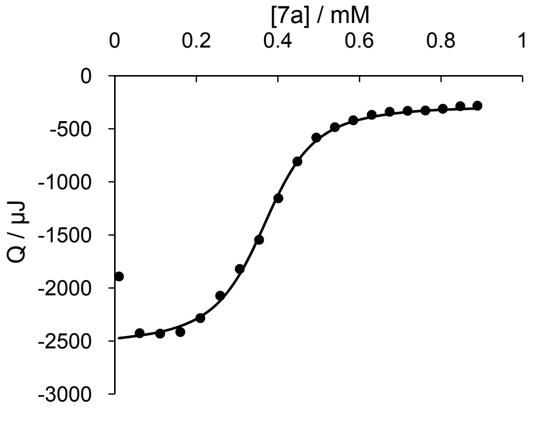
</td><td>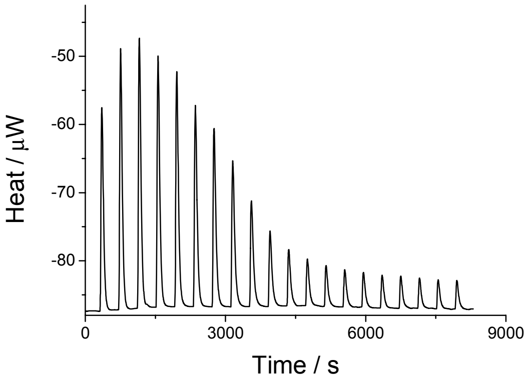
</td></tr>
<tr><td colspan="2">Figure 3.2. Integrated injection peaks of the ITC experiment of 7a with 14 (left) and raw heat. effects (right).</td></tr>
</table>

3.3	Table 1, Entry C
[7b]: 5 mM, [β-CD] : 1 mM	T = 23 °C, Rpm = 300, 20 x 10 μL injections
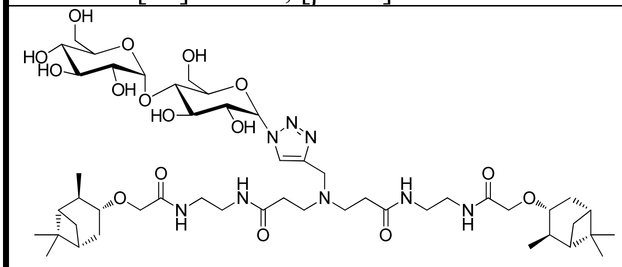	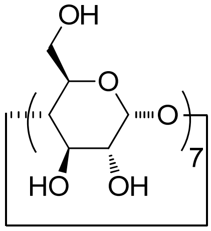
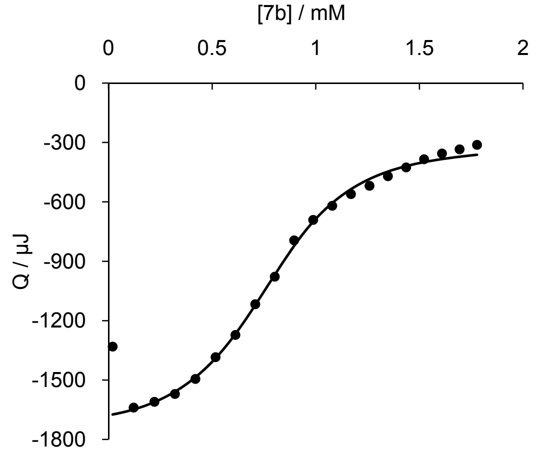	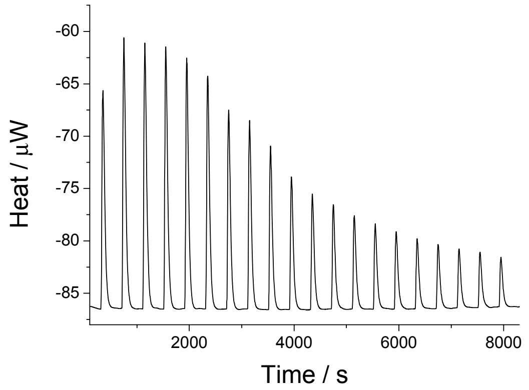

Figure 3.3. Integrated injection peaks of the ITC experiment of **7b** with β-CD (left) and raw heat effects (right).

3.4	Table 1, Entry D
[7b]: 5 mM, [14] : 0.5 mM	T = 23 °C, Rpm = 300, 20 x 10 μL injections
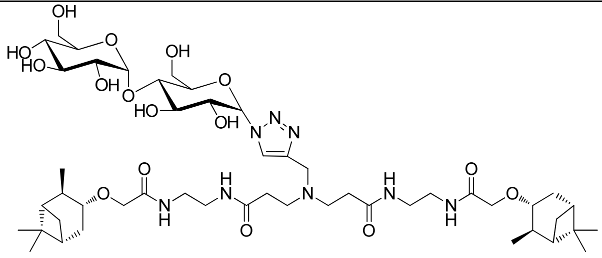	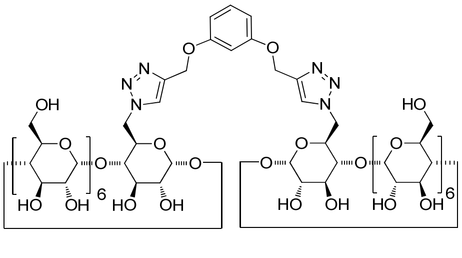
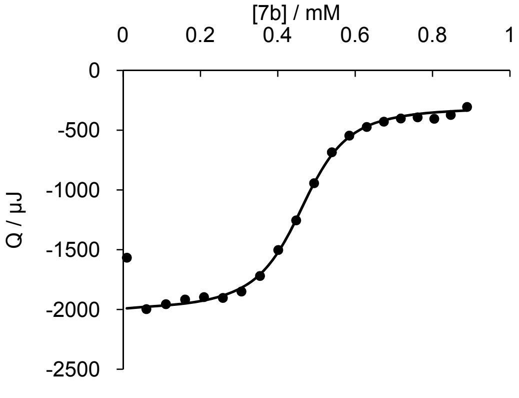	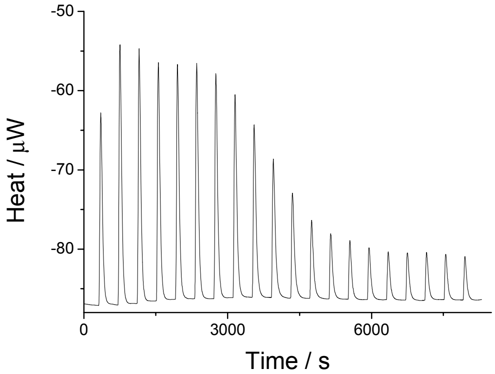

Figure 3.4. Integrated injection peaks of the ITC experiment of **7b** with **14** (left) and raw heat effects (right).

3.5	Table 1, Entry E
[7c]: 5 mM, [β-CD] : 1 mM	T = 23 °C, Rpm = 300, 20 x 10 μL injections
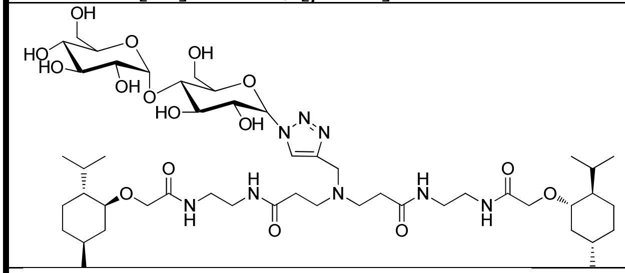	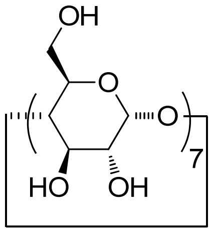
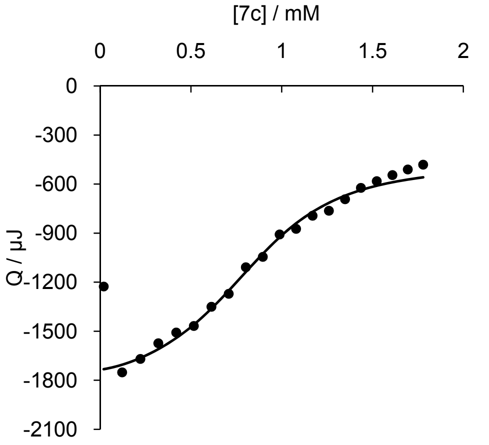	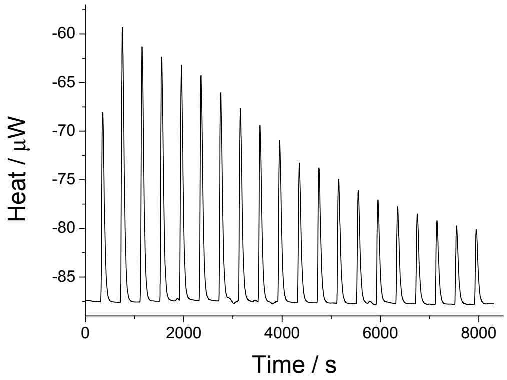

Figure 3.5. Integrated injection peaks of the ITC experiment of **7c** with β-CD (left) and raw heat effects (right).

3.6	Table 1, Entry F
[7c]: 5 mM, [14] : 0.5 mM	T = 23 °C, Rpm = 300, 20 x 10 μL injections
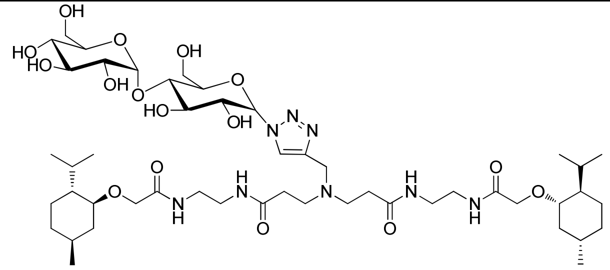	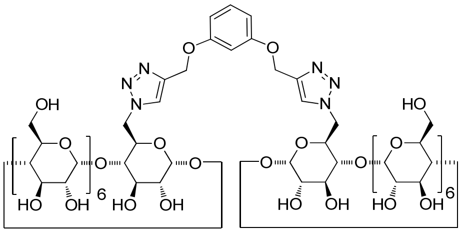
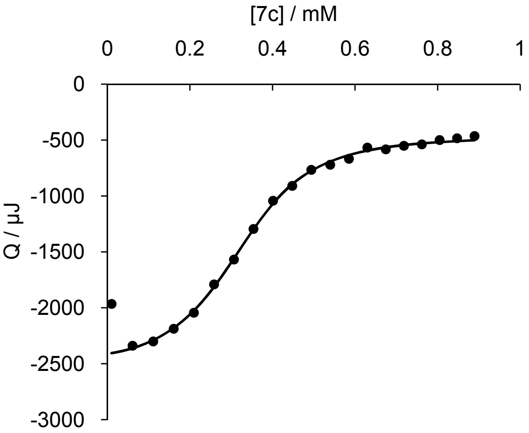	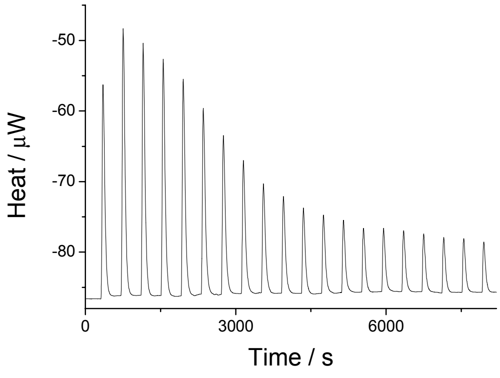

Figure 3.6. Integrated injection peaks of the ITC experiment of **7c** with **14** (left) and raw heat effects (right).

3.7	Table 1, Entry G
[7d]: 5 mM, [β-CD] : 1 mM	T = 23 °C, Rpm = 300, 20 x 10 μL injections
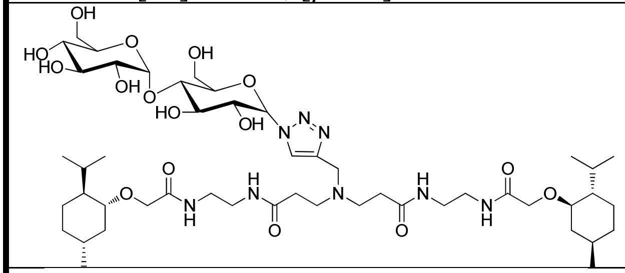	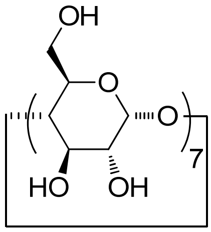
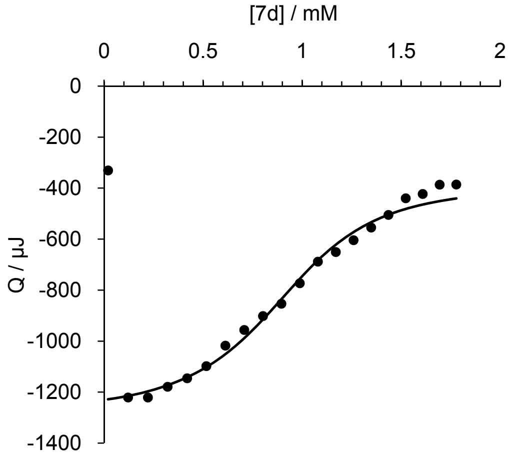	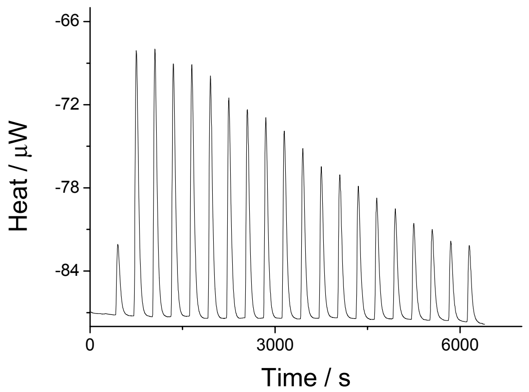

Figure 3.7. Integrated injection peaks of the ITC experiment of **7d** with β-CD (left) and raw heat effects (right).

3.8	Table 1, Entry H
[7d]: 5 mM, [14] : 0.5 mM	T = 23 °C, Rpm = 300, 20 x 10 μL injections
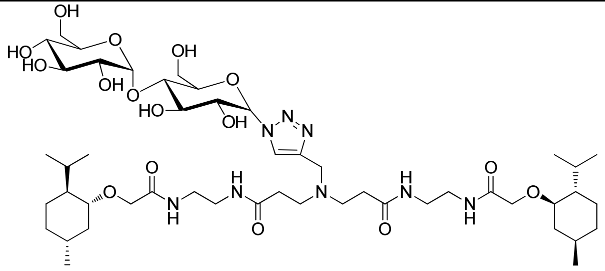	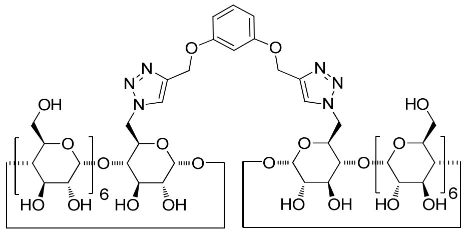
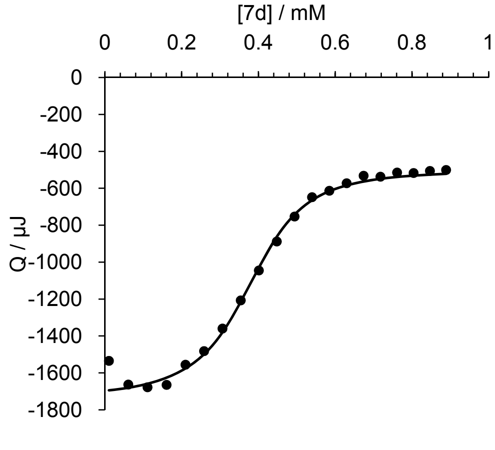	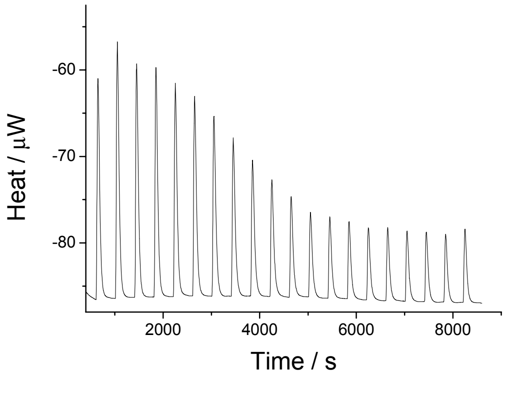

Figure 3.8. Integrated injection peaks of the ITC experiment of **7d** with **14** (left) and raw heat effects (right).

<table>
<tr><th>3.9</th><th>Table 1, Entry I</th></tr>
<tr><td>[7e]: 5 mM, [β-CD] : 1 mM</td><td>T = 23 °C, Rpm = 300, 20 x 10 μL injections</td></tr>
<tr><td>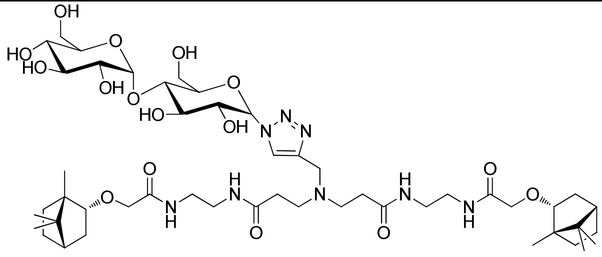</td><td>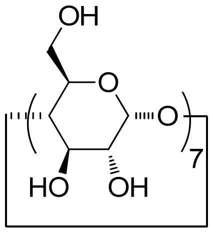</td></tr>
<tr><td>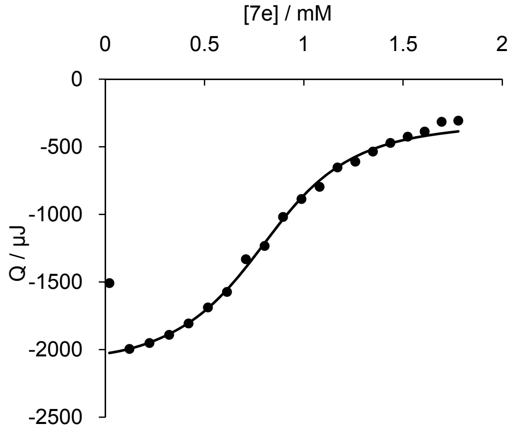
</td><td>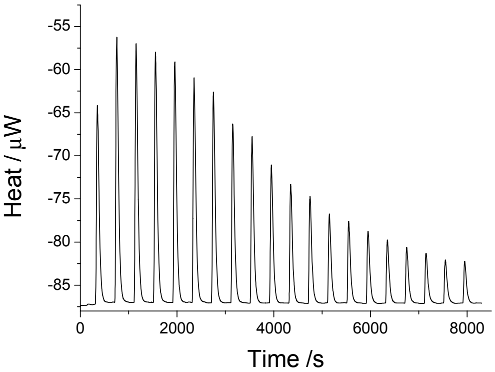
</td></tr>
<tr><td colspan="2">Figure 3.9. Integrated injection peaks of the ITC experiment of 7e with β-CD (left) and raw heat effects (right).</td></tr>
<tr><th>3.10</th><th>Table 1, Entry J</th></tr>
<tr><td>[7e]: 5 mM, [14] : 0.5 mM</td><td>T = 23 °C, Rpm = 300, 20 x 10 μL injections</td></tr>
<tr><td>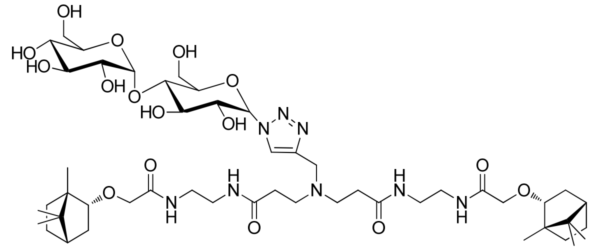</td><td>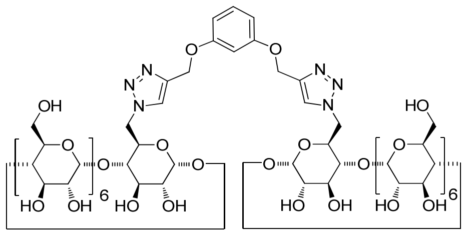</td></tr>
<tr><td>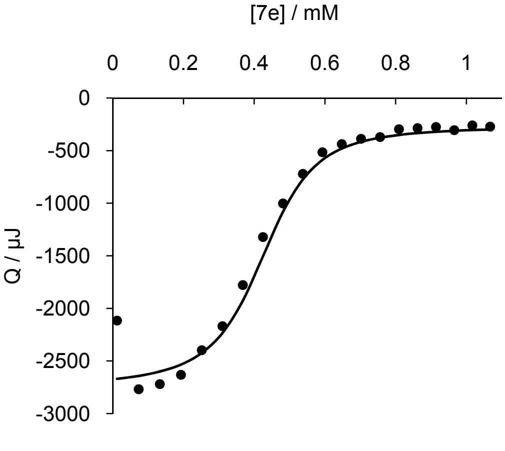
</td><td>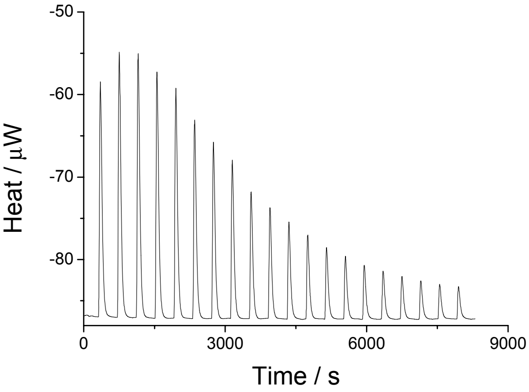
</td></tr>
<tr><td colspan="2">Figure 3.10. Integrated injection peaks of the ITC experiment of 7e with 14 (left) and raw heat effects (right).</td></tr>
</table>

3.11	Table 1, Entry K
[7f]: 5 mM, [β-CD] : 1 mM	T = 23 °C, Rpm = 300, 20 x 10 μL injections

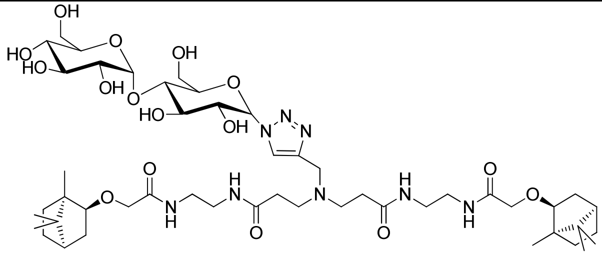

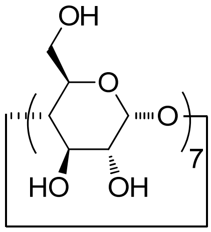

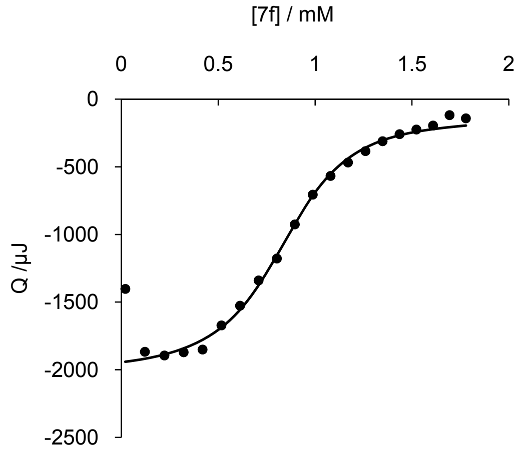

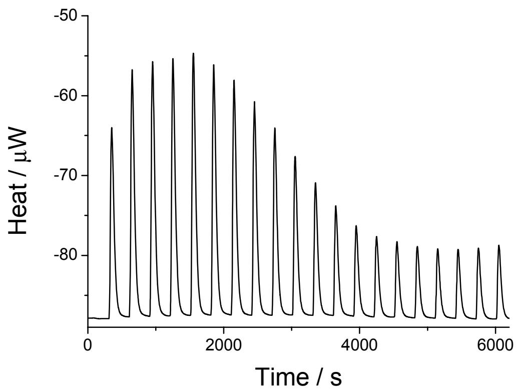

Figure 3.11. Integrated injection peaks of the ITC experiment of **7f** with β-CD (left) and raw heat effects (right).

3.12	Table 1, Entry L
[7f]: 5 mM, [14] : 0.5 mM	T = 23 °C, Rpm = 300, 20 x 10 μL injections

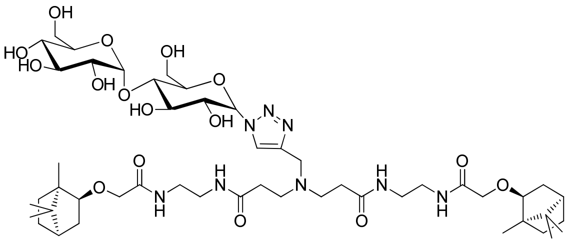

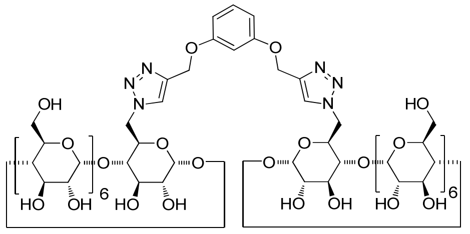

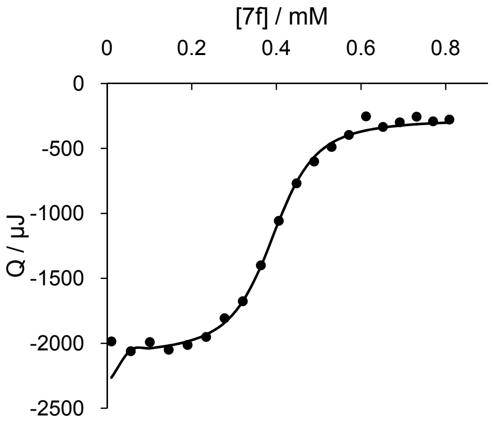

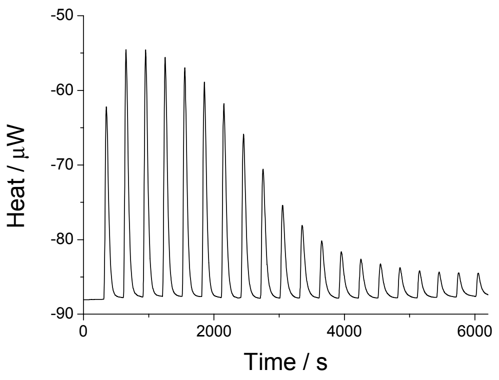

Figure 3.12. Integrated injection peaks of the ITC experiment of **7f** with **14** (left) and raw heat effects (right).

4. Selected NMR Spectra

4.1. ^{1}H-NMR Spectrum of 6a (400 MHz, $CDCl_3$, 298 K)

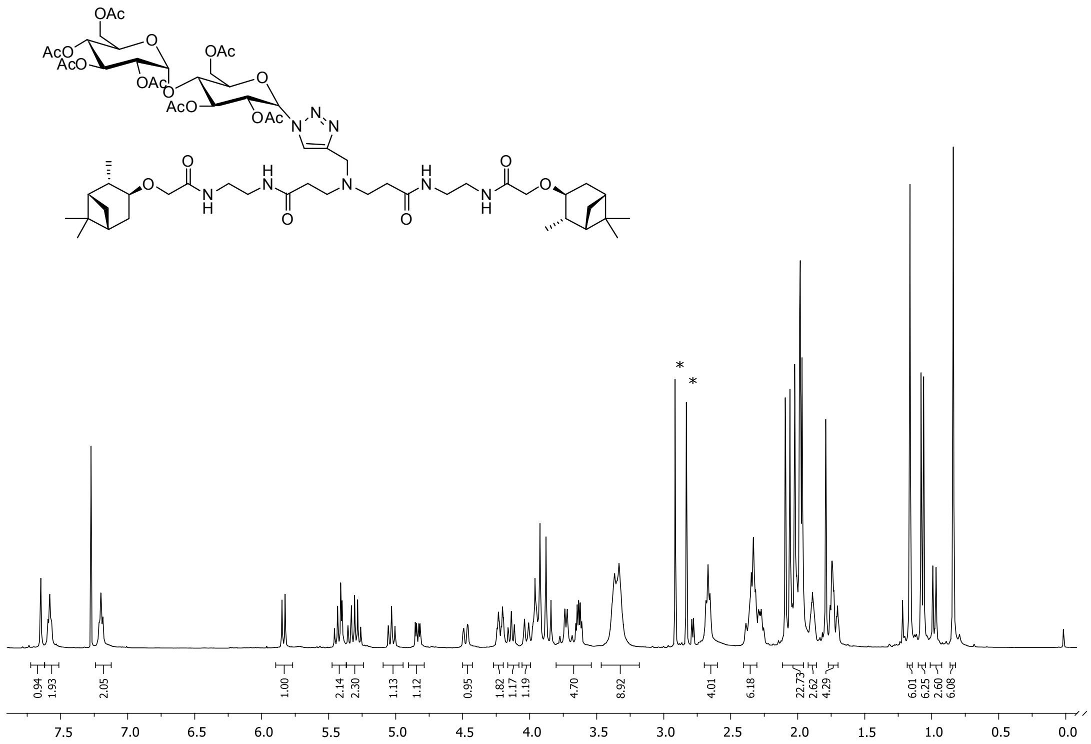

4.2. ^{13}C-NMR spectrum of 6a (100 MHz, $CDCl_3$, 298 K)

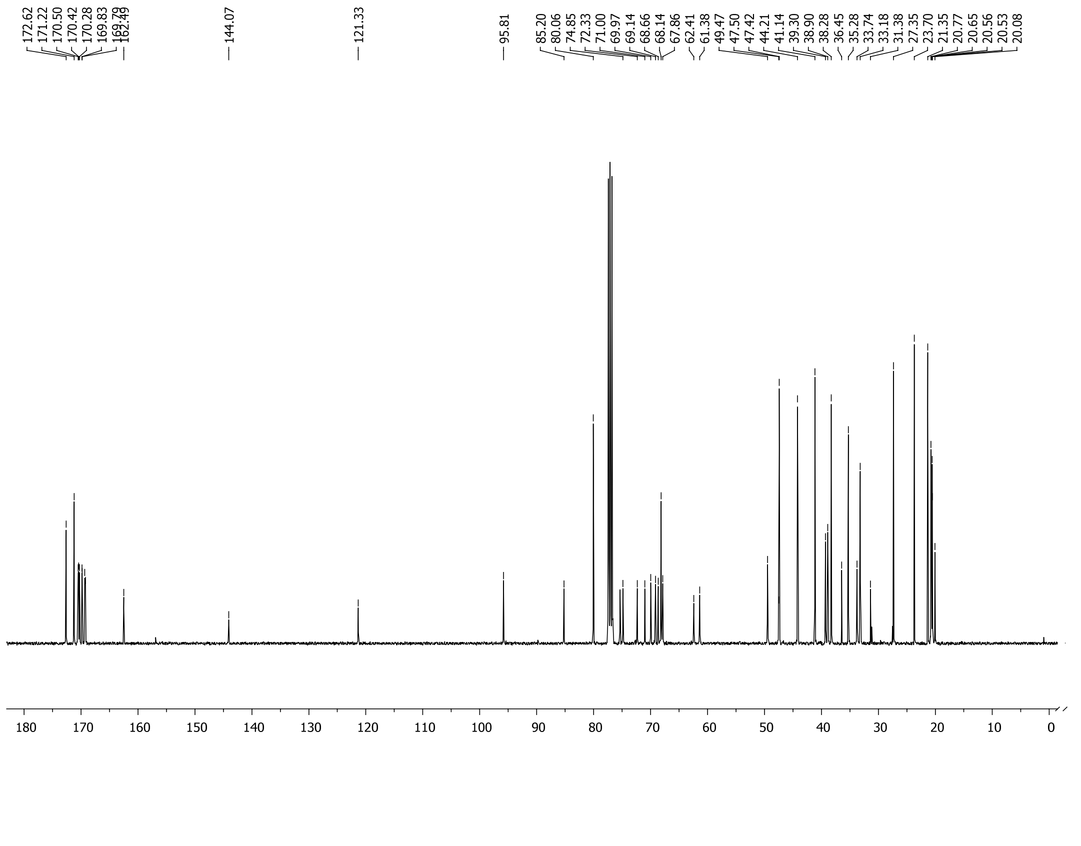

4.3. ^{1}H-NMR Spectrum of 6c (400 MHz, $CDCl_3$, 298 K)

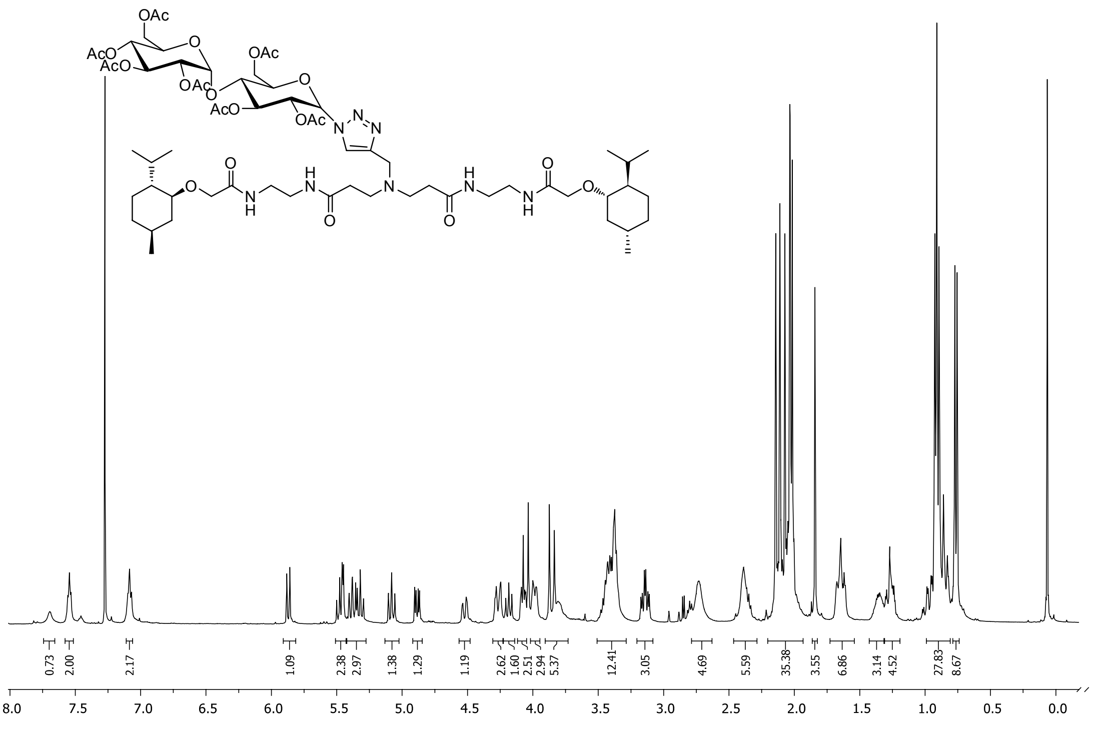

4.4. ^{13}C-NMR Spectrum of 6c (100 MHz, $CDCl_3$, 298 K)

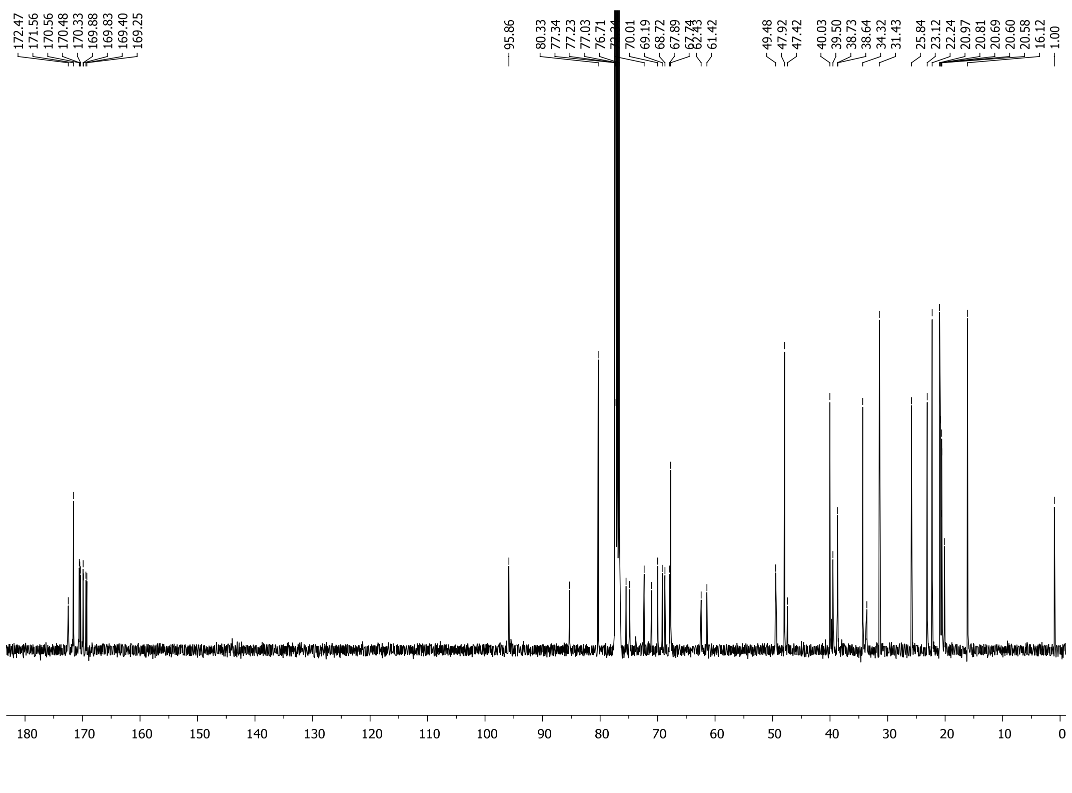

4.5. ^{1}H-NMR Spectrum of 6e (400 MHz, $CDCl_3$, 298 K)

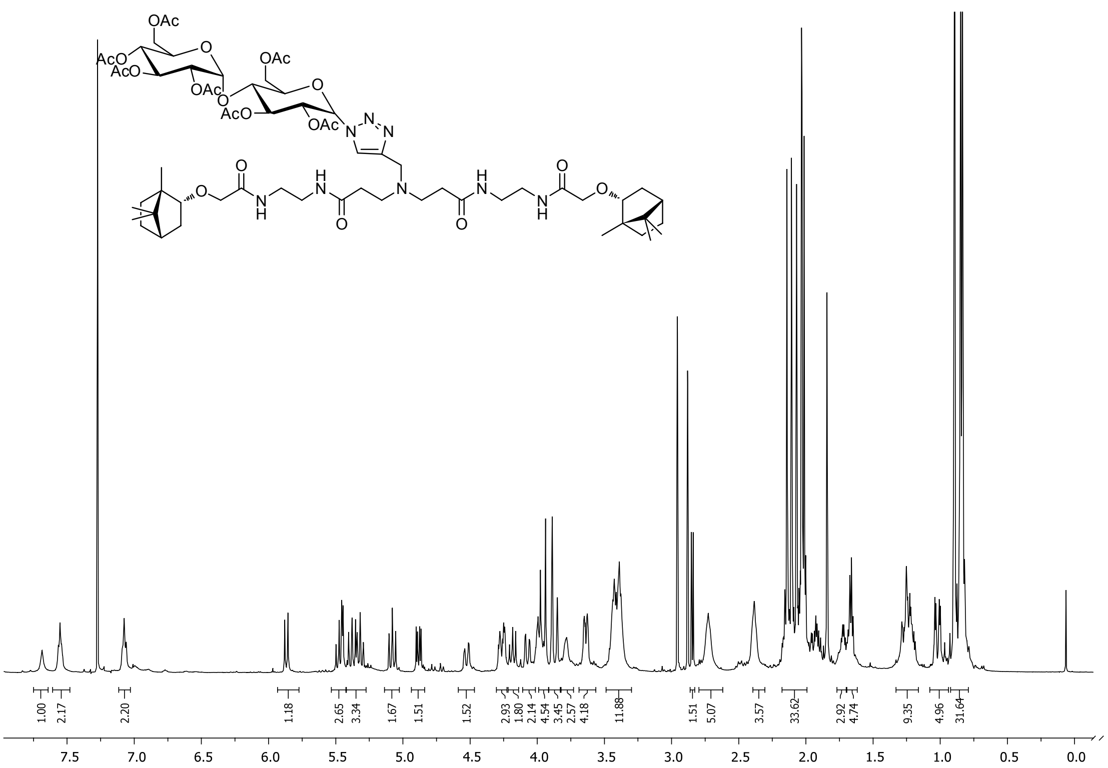

4.6. ^{13}C-NMR Spectrum of 6e (100 MHz, $CDCl_3$, 298 K)

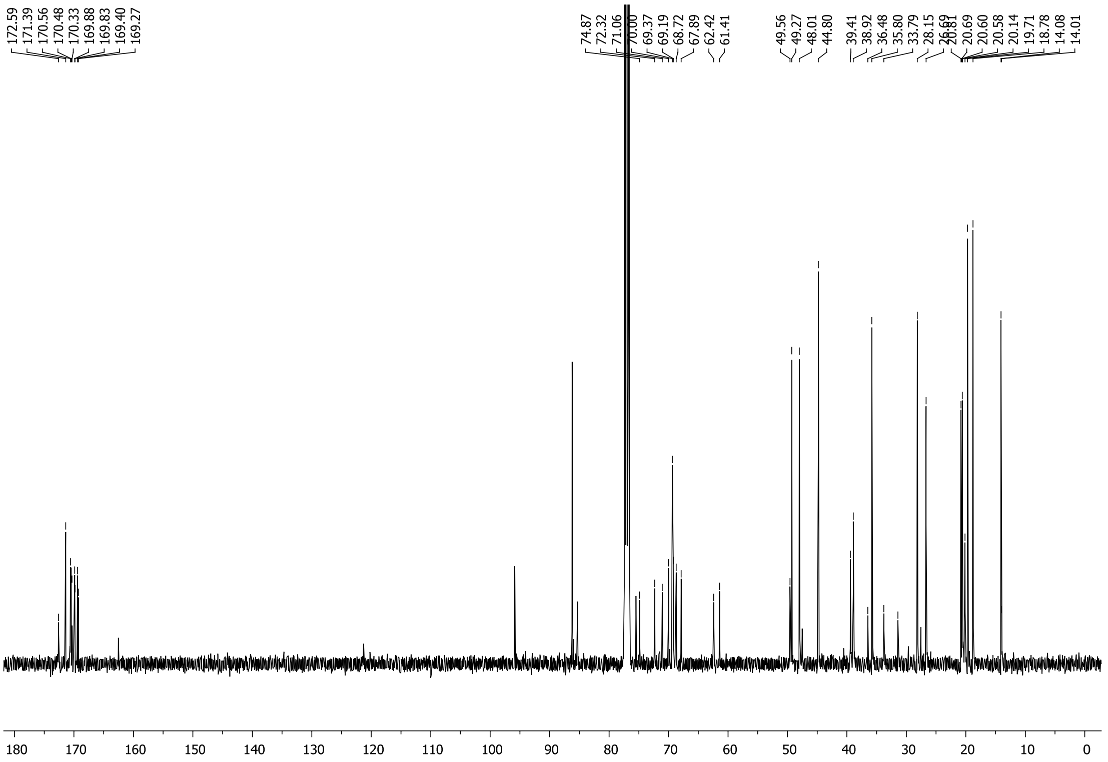

4.7. ^{1}H-NMR Spectrum of 7c (400 MHz, CD_3OD, 298 K)

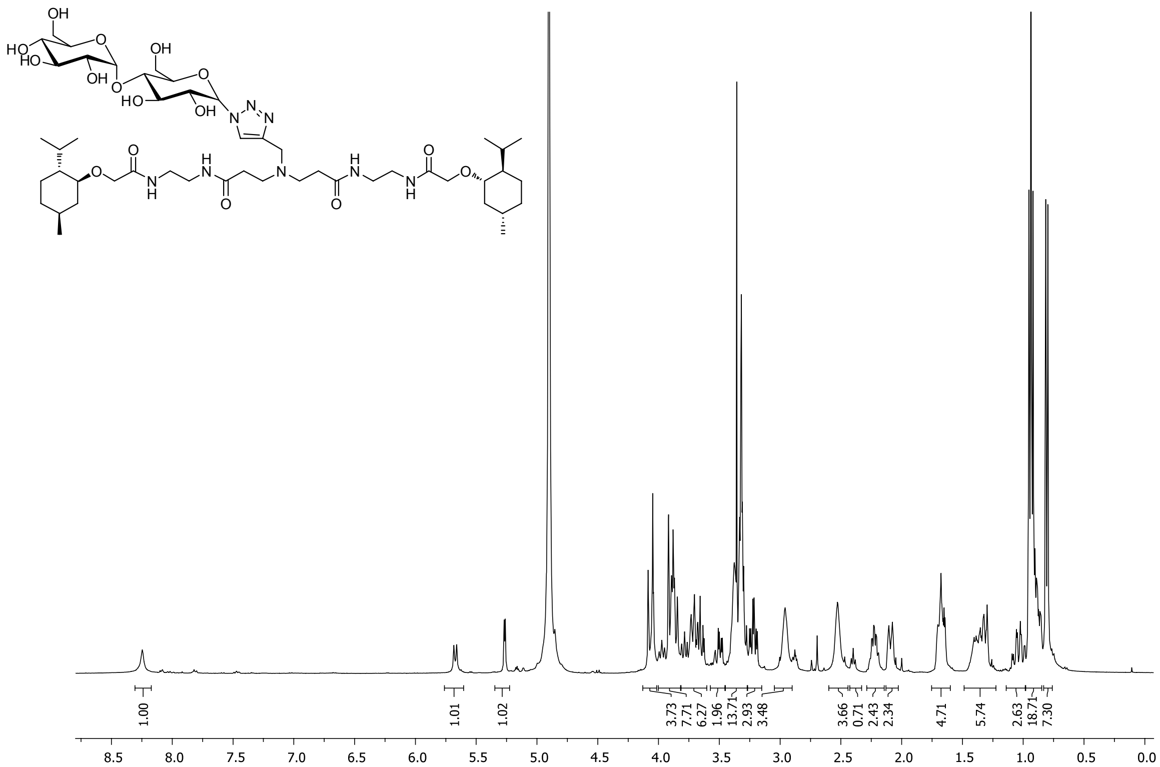

4.8. ^{13}C-NMR Spectrum of 7c (100 MHz, CD_3OD, 298 K)

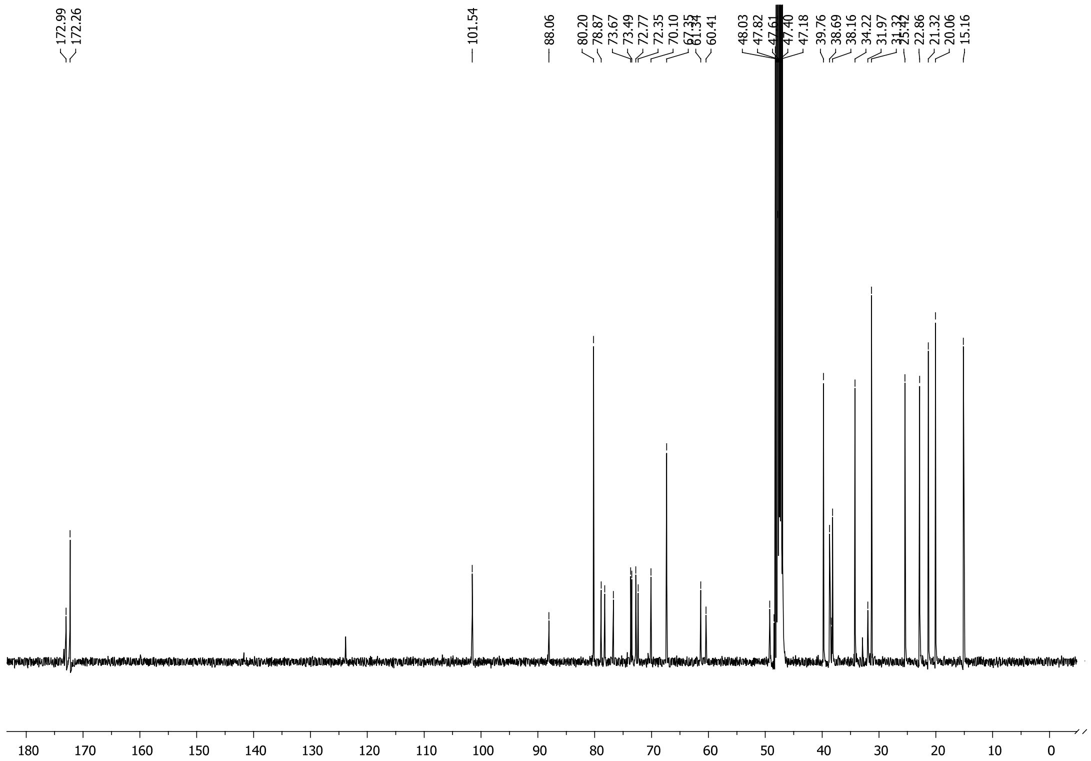

4.9. ^{1}H-NMR Spectrum of 7a (400 MHz, CD_3OD, 298 K)

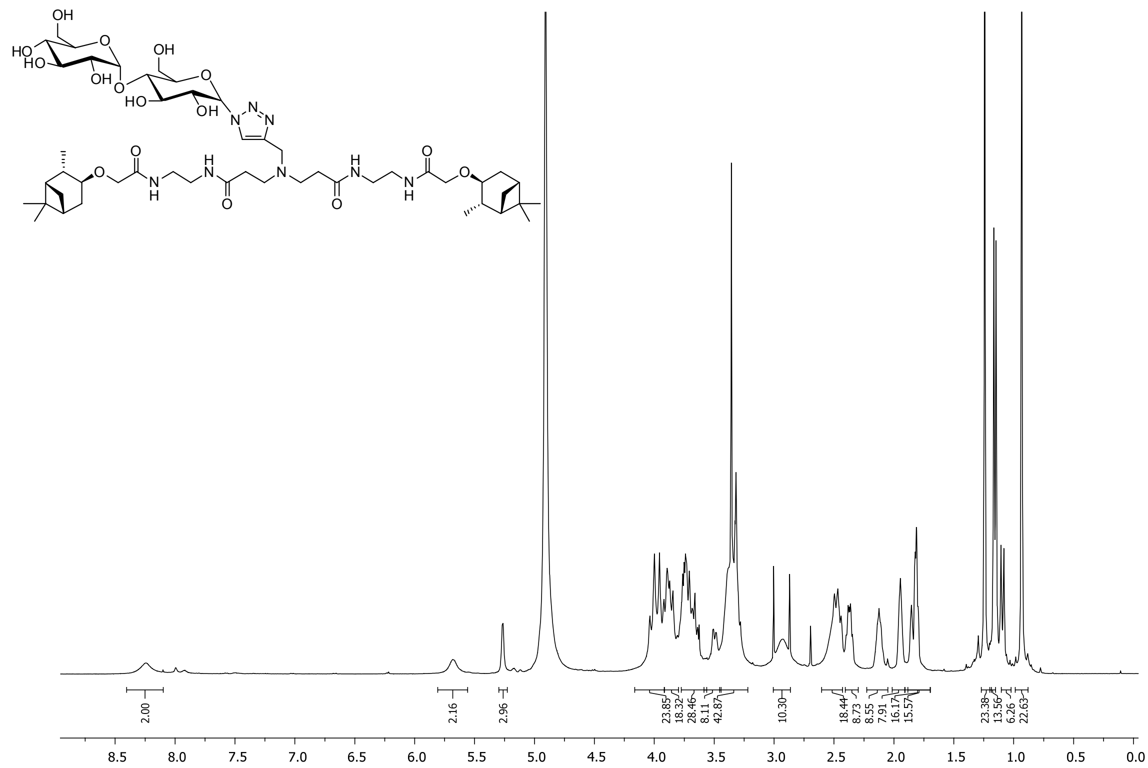

4.10. ^{13}C-NMR Spectrum of 7a (100 MHz, CD_3OD, 298 K)

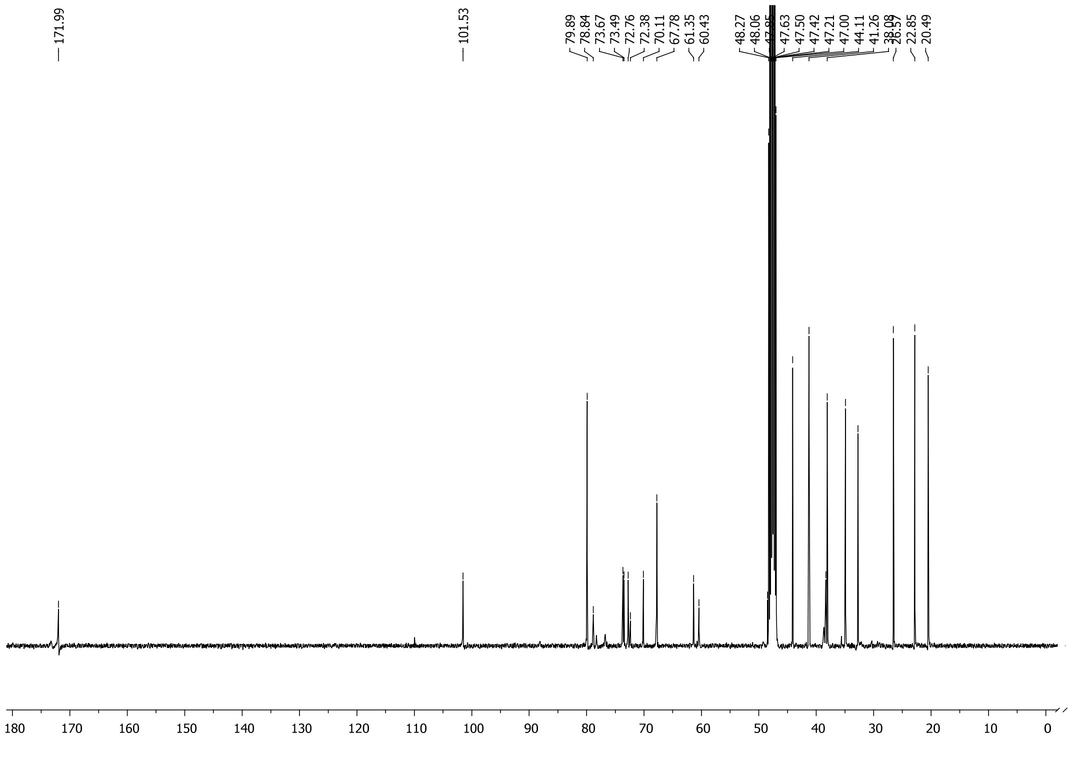

4.11. ^{1}H-NMR Spectrum of 7f (400 MHz, CD_3OD, 298 K)

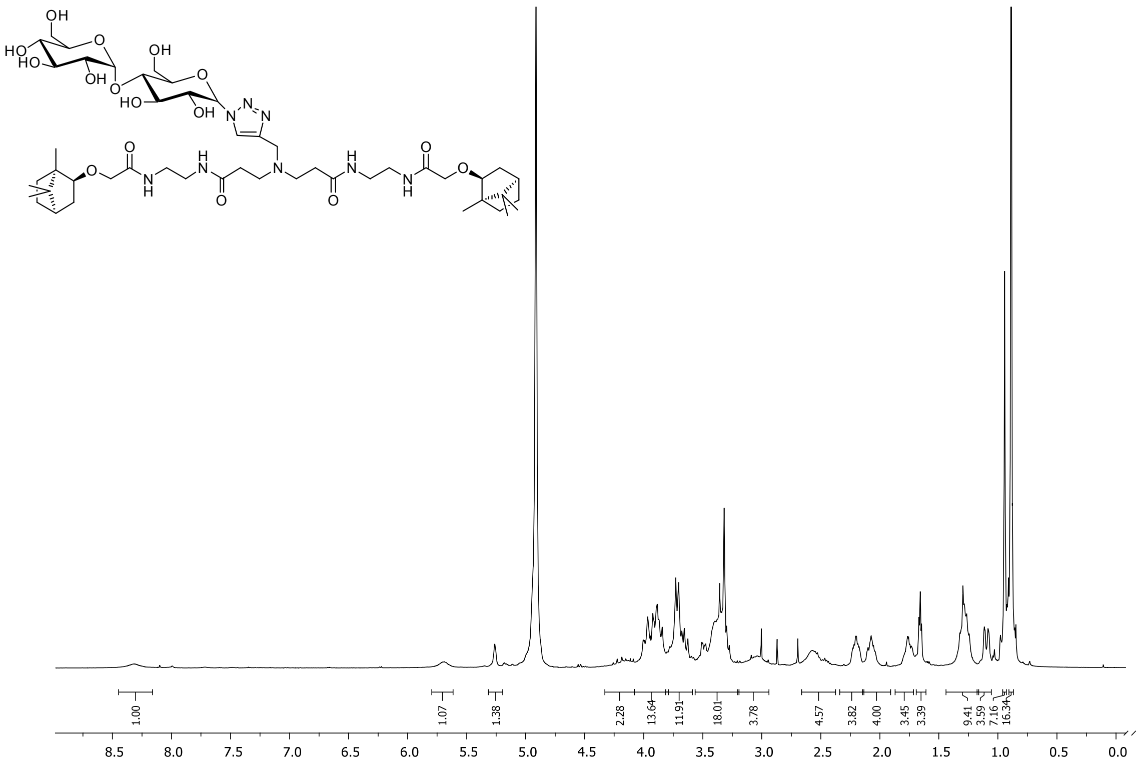

***4.12** ^{13}C-NMR spectrum of compound **7f** (100 MHz, CD_3OD, 298 K)*

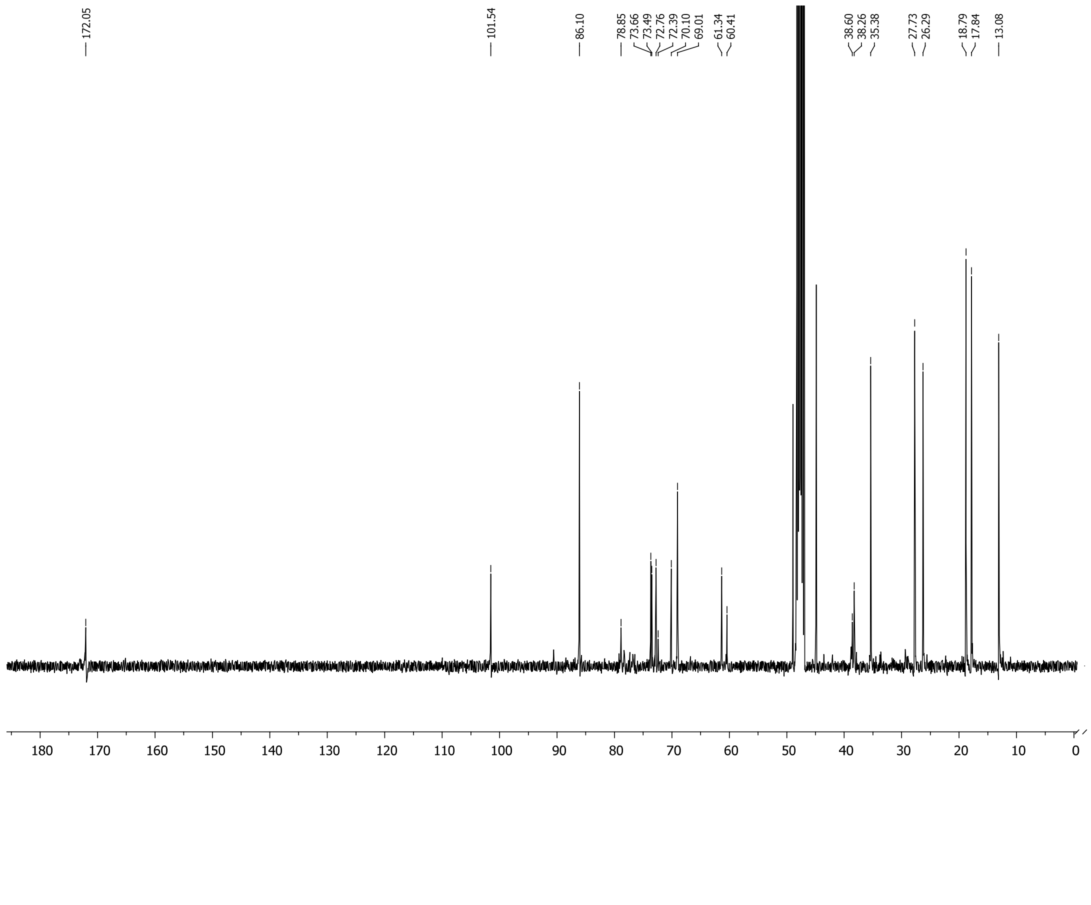

References and Notes

1. Ma, T.; Gao, Q.; Chen, Z.; Wang, L.; Liu, G. Chemical Resolutions of (±)-Calanolide A, (±)-Cordatolide A and Their 11-Demethyl Derivatives. *Bioorg. Med. Chem. Lett.* **2008**, *18*, 1079–1083.
2. Lee, J.W.; Kim, J.H.; Kim, B.K.; Kim, J.H.; Shin, W.S.; Jin, S.H. Synthesis of Symmetrical and Unsymmetrical PAMAM Dendrimers by Fusion between Azide- and Alkyne-Functionalized PAMAM Dendrons. *Bioconjugate Chem*. 2007, *18*, 579–584.
3. Ravidranathan, K.P.; Field, R.A. Iodine: A versatile reagent in carbohydrate chemistry IV. Per-O-acetylation, regioselective acylation and acetolysis. *Tetahedron* **1997**, *53*, 11753–11766.
4. Peto, C.; Batta, G.; Gyorgydeak, Z.; Sztaricskai, F. Synthesis of the anomers of hepta-O-acetylcellobiosyl azide, hepta-O-lactosyl azide, hepta-O-maltosyl azide, and hepta-O-melibiosyl azide. *Liebigs Ann. Chem*. **1991**, 505–507.
5. Simon, D.Z.; Brookman, S.; Beliveau, J.; Salvador, R.L. Synthetic acetylenic antifungal agents. *J. Pharm. Sci.* 1977, *66*, 431–432.
6. Brady, B.; Lynam, N.; Darcy, R. 6^A-O-p-toluenesulfonyl-betacyclodextrin. *Org. Synth.* **2000**, *77*, 220.
7. Petter, R.C.; Salek, J.S.; Sikorski, C.T.; Kumaravel, G. Lin, F.-T. Cooperative binding by aggregated mono-6-alkylamino-beta-cyclodextrins. *J. Am. Chem. Soc.* **1990**, *112*, 3860–3868.
8. Sallas, F.; Marsura, A.; Petot, V.; Pinter, I.; Kovacs, J.; Jicsinszki, L.; Synthesis and study of new beta-cyclodextrin dimers having a metal coordination center and carboxamide or urea linkers. *Helv. Chim. Act*. **1998**, *81*, 632–645.
9. The ^{1}H-NMR spectrum shows doubled signals indicating a second species in aqueous media. Due to this fact, only extremely broadened signals in the ^{13}C spectrum were obtained. For a complete discussion of the structure see the explanation in the paper.

© 2011 by the authors; licensee MDPI, Basel, Switzerland. This article is an open access article distributed under the terms and conditions of the Creative Commons Attribution license (http://creativecommons.org/licenses/by/3.0/).
